# Associations for Sense of Purpose with Smoking and Health Outcomes Among Adults with Diabetes

**DOI:** 10.1007/s12529-023-10191-0

**Published:** 2023-07-06

**Authors:** Sara J. Weston, Patrick L. Hill, Daniel K. Mroczek

**Affiliations:** 1 Department of Psychology, University of Oregon, 1451 Onyx St, Eugene, OR 97403 USA; 2 Department of Psychological and Brain Sciences, Washington University in St. Louis, St. Louis, USA; 3 Department of Medical Social Sciences, Northwestern University, Chicago, USA; 4 Department of Psychology, Northwestern University, Evanston, USA

**Keywords:** Purpose, Diabetes, Coordinated analysis, Health behavior, Heart disease, Self-rated health

## Abstract

**Background:**

Health complications from diabetes place major strain on individuals, financially and emotionally. The onset and severity of these complications are largely driven by patients’ behaviors, making psychosocial factors that influence behaviors key targets for interventions. One promising factor is sense of purpose or the degree to which a person believes their life has direction.

**Method:**

The current study investigated whether sense of purpose predicts self-rated health, cardiovascular disease, and smoking status among adults with diabetes concurrently and prospectively. Moreover, it tested whether these associations held across multiple samples and cultures. Coordinated analysis using 12 datasets cross-sectionally and eight longitudinally (total *N* = 7277) estimated the degree to which sense of purpose is associated with subjective health, smoking status, and cardiovascular disease among adults with diabetes. Coordinated analysis allows for greater generalizability of results across cultures, time periods, and measurement instruments. Datasets were included if they concurrently included a measure of sense of purpose and diabetes status and at least one health measure: self-rated health, current smoking status, or heart condition status.

**Results:**

Sense of purpose was associated with higher self-rated health, smoking status, and cardiovascular disease cross-sectionally and self-rated health prospectively. Purpose was unassociated with changes in health over time.

**Conclusion:**

These results highlight the relationship of a key individual difference, sense of purpose, to the behaviors and outcomes of adults with diabetes. While more research is needed to determine the boundaries of this relationship, it seems sense of purpose may be considered in the future as a potential target for intervention.

**Supplementary Information:**

The online version contains supplementary material available at 10.1007/s12529-023-10191-0.

## Introduction

Diabetes is among the more prominent chronic conditions, at least in the USA, and is one that requires consistent treatment via lifestyle behaviors [1]. As such, significant resources are spent on reminders and interventions to encourage adults with diabetes to maintain consistent self-care, and thus, it would be valuable to identify psychosocial variables predict health outcomes among those with diabetes [2]. The current study addressed this call with respect to sense of purpose in life, given its linkages to glucoregulation [3, 4] and mention within efforts to improve diabetes care through patient psychosocial resources [5–7]. Specifically, this work employed coordinated data analysis to examine whether sense of purpose predicted greater self-rated health, reduced risk of cardiovascular disease, and lower smoking status across 12 datasets worldwide. Although sense of purpose appears a consistent factor predicting positive health outcomes [8–10], work is needed to understand whether this psychosocial factor still promotes positive health outcomes for individuals currently dealing with a given health ailment.

### Sense of Purpose and Health Indicators

Sense of purpose is assessed as the extent to which one perceives the presence of a direction and life goals [11]. Recent years have demonstrated consistent evidence that adults with a stronger sense of purpose experience better health outcomes. Multiple studies have demonstrated that sense of purpose is positively associated with self-reported health and well-being [12, 13]. Sense of purpose also prospectively predicts later risk for cardiovascular disease [8, 9, 14] and even reduced all-cause mortality risk [8]. One explanation for these findings is that individuals may take better care of themselves when they are focused on long-term goal pursuits. Support for this claim comes from studies showing that purposeful adults tend also to report greater engagement in health behaviors, such as activity engagement and positive dietary practices [12]. Associations also have been found between sense of purpose and objective markers of physical fitness [15, 16]. Moreover, sense of purpose prospectively predicts health behavior engagement [17].

Research also considered purpose–health associations within the context of diabetes, spurred in part by the possibility that diabetes onset could provide a mechanism explaining linkages between sense of purpose and cardiovascular health [9]. For instance, work has shown that sense of purpose predicts better glucose control, measured as lower HbA1c level, among older adults with diabetes [3]. In addition, adults with a higher sense of purpose were generally less likely to develop diabetes or prediabetes over the 4-year time span. Of interest, that study found that the association held even when accounting for mental health and physical activity. Work also suggests that sense of purpose is related to better glucoregulation in non-American samples [4], although more work is needed to consider sense of purpose outside of the USA. One possibility is that sense of purpose has been linked to better healthcare use [18], another important aspect of treatment for diabetes. Initial evidence comes from one study demonstrated that sense of purpose was related to greater medication compliance among older adults with health conditions, which included diabetes among other common conditions [19].

### Coordinated Analyses in the Study of Purpose and Health

In sum, this literature provides reasons to believe that sense of purpose may be valuable for adults with diabetes. However, an important precondition for integrating sense of purpose into personalized diabetes care is to demonstrate that the construct still predicts outcomes like self-rated health and risk for cardiovascular disease among adults with diabetes. One central reason is the difficulty with acquiring large enough longitudinal samples that have a specific chronic condition, as well as the psychosocial measures of interest. Aiding this endeavor is the ability to utilize publicly available data sets. Such data sets reduce the burden on individual researchers to collect sufficiently powered samples, and many are longitudinal, allowing for more robust and conservative tests of associations. Indeed, much of the published literature on purpose and health utilizes such data sets. However, caution must be exercised when conducting research with pre-existing data. Those data sets often contain a substantial number of participants and variables, resulting in a larger potential number of researcher degrees of freedom [20, 21].

The potential for publishing spurious results from pre-existing data can be reduced through the use of coordinated analysis [22, 23]. Coordinated analyses are performed on multiple studies, with models and variables harmonized to improve comparability between studies. Such analyses guard against overindulgence in researcher degrees of freedom, as this method limits potential model complexity to only those analyses that are feasible in many studies. Moreover, spurious results have small to no impact; that is, if a null effect is deemed significant (or substantial) in one study because of random variation, it has a very low chance of being replicated in the other studies in the coordinated analysis and will stand out as an outlier. In this way, the generalizability of an effect across measurement tools, cultures, or years (to name only a few potential moderators) may be assessed.

### Current Study

The current study sought to provide a foundation for understanding both the value of considering sense of purpose among adults with diabetes. Past work has frequently suggested including targeting purpose in healthcare programs to promote diabetes care, and as such, work is needed first to demonstrate that sense of purpose is consistently linked to better health among those with this chronic condition. A coordinated data analysis across 12 samples investigated the associations between sense of purpose and three outcomes of interest — self-rated health, cardiovascular disease, and smoking status — among adults with diabetes. For all three variables, we examined whether sense of purpose was concurrently associated with the variable, as well as longitudinally predictive of the variable. Self-rated health [12, 13] and cardiovascular disease [8, 9] have been consistently linked to sense of purpose. As a comparison, we also chose smoking status to provide a health behavior that has shown inconsistent associations with sense of purpose [17], to examine whether sense of purpose may exhibit wider lifestyle benefits when considering a sample with a chronic condition. That said, the choice of smoking status also was one of convenience, as it was one of the few health behaviors assessed across multiple datasets.

The current study considered whether there was significant heterogeneity in the estimates for the tested associations across the worldwide samples. Of particular interest is whether the associations are similar among American and non-American samples, given that the USA leads the sampled nations in prevalence of diabetes [24]. Therefore, we examined whether associations were similar in American versus non-American studies, as well as other moderators, such as measurement properties and study length.

## Methods

### Data Sets

Data sets were identified through the Inter-university Consortium for Political and Social Research (icpsr.umich.edu). Data sets were included in these analyses if they met the following criteria: (A) included a measure of sense of purpose; (B) a measure of diabetes status; (C) included at least a measure of self-rated health, current smoking status, or heart condition status; and (D) measures had to have been assessed concurrently. A total of 12 studies were identified for inclusion in the current study (see Supplemental Table 1 and Supplementary File 1). This analysis was deemed exempt from human subjects review due to the secondary data analysis.

#### Americans’ Changing Lives (ACL)

The ACL Survey is a nationally representative longitudinal study focusing on the differences between Black and White Americans in middle and late life [25]. Data collection began in 1986 with adults over the age of 25; Black Americans and adults age 60 years or older were over-sampled at this time. Wave 4 was the only wave of data collection (which took place in 2001 and 2002) that included a measure of sense of purpose. At this time, 228 participants with diabetes (65% female, 36% non-white) had data on sense of purpose and health outcomes. The average age of these participants was 64.55 (SD = 12.27). Participants had, on average, 12.22 years of education (SD = 2.40).

#### Australian Longitudinal Study of Aging (ALSA)

The ALSA sought to examine the health and well-being of adults aged 70 and over living in Australia [26]. Data collection began in 1992, and sense of purpose was first assessed in 1994. At this time, 145 participants with diabetes (44% female) had data on sense of purpose and health outcomes. Race was not included in this study, as race was not assessed and 98% of participants identified their ethnicity as Australian. The average age of participants was 79.16 (SD = 6.16). Participants had, on average, 9.60 years of education (SD = 3.25).

#### Changing Lives of Older Couples (CLOC)

The Changing Lives of Older Couples study is a multi-wave prospective study of spousal bereavement [27]. Initial data collection began in 1987 and targeted older, married couples in the Detroit metropolitan area. Participants were interviewed as a couple, and follow-up times were based on when the first spouse died. Only the third wave of data collection (48 months after spouse’s death) contained a sense of purpose assessment. At this time, 27 participants with diabetes (85% female, 26% non-white) had data on sense of purpose and health outcomes. The average age of participants was 77.33 (SD = 5.90). Participants had, on average, 11.78 years of education (SD = 3.24).

#### English Longitudinal Study of Ageing (ELSA)

The ELSA sought to understand aging and quality of life in the UK for people over the age of 50 [28]. It was patterned after the Health and Retirement Study in the USA. Sense of purpose was first assessed in Wave 2 (2004) of data collection. At this time, 27 participants with diabetes (48% female, 0% non-white) had data on sense of purpose and health outcomes. The average age of participants was 69.63 (SD = 9.99). Education was scored as 1 (less than high school), 2 (high school graduate), 3 (some college), and 4 (college graduate and above; M = 2.04, SD = 1.13).

#### Health and Retirement Study (HRS)

The HRS is a national panel study of older adults in America [29]. Sense of purpose was first administered to a subset of participants in 2006; an additional subset received the scale in 2008. At this time, 4218 participants with diabetes (54% female, 33% non-white) had data on sense of purpose and health outcomes. The average age of participants was 67.05 (SD = 10.21). Participants had, on average, 11.92 years of education (SD = 3.43).

#### Korean General Social Survey (KGSS)

The KGSS is the South Korean version of the General Social Survey [30]. Sense of purpose was first assessed in 2009. At this time, 72 participants with diabetes (57% female) had data on sense of purpose and health outcomes. Race was not assessed by the KGSS and was not included in these analyses. The average age of participants was 61.67 (SD = 12.45). Education was measured on a scale from 1 (no formal schooling) to 7 (graduate school/Ph.D.; M = 3.35, SD = 1.81).

#### Survey of Midlife in Japan (MIDJA)

The MIDJA follows a sample of Japanese adults in the Tokyo metropolitan area [31]. Measures parallel those of the Midlife in the United States Study. Sense of purpose was first assessed in 2008. At this time, 67 participants with diabetes (33% female) had data on sense of purpose and health outcomes. Race was not assessed by the MIDJA and was not included in these analyses. The average age of participants was 63.43 (SD = 10.12). Education was measured on a scale from 1 (8th grade/junior high graduate) to 8 (graduate school; M = 4.43, SD = 2.30).

#### Midlife in the United States (MIDUS)

The MIDUS is a national panel study patterns, predictors, and consequences of midlife development in the USA [32]. The sense of purpose scale was included in the first wave of data collection in 1992. At this time, 308 participants with diabetes (44% female, 16% non-white) had data on sense of purpose and health outcomes. The average age of participants was 55.35 (SD = 12.04). Education was measured on a scale from 1 (no school) to 12 (Ph.D. or professional degree; M = 6.27, SD = 2.68).

#### Project STRIDE: Stress, Identity, and Mental Health (STRIDE)

Project STRIDE examined the effect of stress and minority identity on mental health [33]. Participants were residents of the New York City area. Sense of purpose was assessed in 2004. At this time, 17 participants with diabetes (35% female, 65% non-white) had data on sense of purpose and health outcomes. The average age of participants was 35.94 (SD = 9.85). For education, participants reported whether they had less than or equivalent to a high school diploma (18%), whether they had completed some college but not received a degree (28%) and whether they had a received a bachelor’s degree (53%). Responses were converted into numeric values representing approximately the amount of time participants spend in formal schooling (5 years for less than a high school diploma, 12 years for a high school degree, 14 for more than a high school diploma but less than a bachelor’s degree, and 16 years for a bachelors). Participants had, on average, 11.53 years of education (SD = 5.04).

#### Study of Women’s Health Across the Nation (SWAN)

SWAN was designed to examine how mid-life experiences affect the health and well-being of women [34]. A sense of purpose scale was included in the screener. At this time, 1014 participants with diabetes (65% non-white) had data on sense of purpose and health outcomes. The average age of participants was 48.23 (SD = 4.41). Education was measured on a scale from 1 (less than high school) to 5 (post(college) graduate education; M = 2.69, SD = 1.15).

#### United States National Health Measurement Survey (USNHMS)

USNHMS aimed to cross-calibrate several measures of health-related quality of life among older US adults [35]. This was a cross-sectional survey, with data collected in 2005 and 2006. At this time, 720 participants with diabetes (58% female, 47% non-white) had data on sense of purpose and health outcomes. The average age of participants was 65.00 (SD = 12.35). Education was measured on a scale from 1 to 18, with 1 through 12 corresponding to grade graduated through secondary school, 13 through 16 corresponding to the number of years in college, 17 corresponding to a master’s degree, and 18 corresponding to a Ph.D. or professional degree (M = 12.79, SD = 2.88).

#### Wisconsin Longitudinal Study (WLS)

WLS is a long-term study following a random sample of people who graduated from Wisconsin high schools in 1957 and some of their siblings [36]. Sense of purpose was first assessed in 1992. At this time, 440 participants with diabetes (47% female) had data on sense of purpose and health outcomes. Racial identity was not included in the publicly available data set. The average age of participants was 54.51 (SD = 4.84). Participants had, on average, 13.32 years of education (SD = 2.17).

Data sets were included in longitudinal analyses if they had additional measurement occasions after the initial measurement of sense of purpose. Studies included in these analyses were the ACL study (*N*_participants_ = 132, *N*_observations_ = 264), with a total of two waves of data collection across 11 years; the ALSA study (*N*_p_ = 145, *N*_obs_ = 435), with three waves of data collection across 2 years; the ELSA study (*N*_p_ = 27, *N*_obs_ = 122), with five waves of data collection across 8 years; the HRS study (*N*_p_ = 4531, *N*_obs_ = 15,964), with six waves of data collection across 10 years; the KGSS study (*N*_p_ = 72, *N*_obs_ = 205), with four waves of data collection across 3 years; the MIDJA study (*N*_p_ = 67, *N*_obs_ = 107), with two waves of data collection across 4 years; the MIDUS study (*N*_p_ = 308, *N*_obs_ = 582), with three waves of data collection across 18 years; the SWAN study (*N*_p_ = 1014, *N*_ob_s = 2150), with 11 waves of data collection across 10 years; and the WLS study (*N*_p_ = 440, *N*_obs_ = 986), with three waves of data collection across 19 years.

### Measures

#### Sense of Purpose

Nearly all studies measured purpose by adapting the Purpose in Life sub-scale of the Psychological Well-Being scale [37, 38]. This measure included items like “I have a sense of direction and purpose in my life” and “I sometimes feel as if I’ve done all there is to do in life” (reverse scored). This scale has a total of 10 items; individual studies administered as few as one item and as many as seven to their participants. Two studies used alternate measures of purpose: the KGSS administered four items, including “I believe I can find the purpose of life, i.e., a reason to live for” and “I still have many things left to do”; SWAN administered three items including “I have a mission or purpose in life” and “My faith sustains me.” Exact items for each study and their relationship to the Ryff scales are presented in Supplementary File 2, Table 7. Descriptive statistics for each study are presented in Supplemental Table 1. Response scales varied for each study; prior to analysis, purpose scores were scaled such that 1 represented the lowest possible level of purpose, and 5 represented the highest. By rescaling continuous measures, regression coefficients are harmonized and comparable across studies.

#### Diabetes

All studies gathered data on whether or not participants had been diagnosed with diabetes at the same time as purpose in life. Most studies used some form of the question, “Has a doctor [or other medical professional] ever told you that you have diabetes?” In the MIDUS and the MIDJA, participants were asked whether they had experience or been treated for diabetes in the last 12 months. In the ALSA, participants reported whether they were currently receiving treatment for diabetes. Responses for all studies were coded as 0 (not diagnosed/treated) and 1 (diagnosed/treated).

#### Self-Rated Health

All studies assessed health using a single-item measure general health. For nearly every study, this item was a variation of the question “In general, how would you rate your health?” with response options “excellent,” “very good,” “good,” “fair,” and “poor.” The WLS included “very poor” and omitted “very good.” The MIDJA used a response scale from 1 to 10 (worst to best possible health); this was rescaled to match the 1–5 scale in the other studies. Responses were coded such that larger values indicate better health.

#### Smoking Status

Most studies included measurement of smoking, although the question wording and response choices varied widely between studies. We recoded all variables such that 0 indicated a participant did not currently smoke and 1 indicated that they did currently smoke.

#### Heart Condition

Most studies gathered data on whether or not participants had been diagnosed with a heart condition of some kind. Most studies used some form of the question, “Has a doctor ever told you that you have heart disease?” Some studies included in this question specific forms of heart disease, such as heart attack, angina, coronary heart disease, and congestive heart failure. The MIDUS asked nine different questions assessing various types of heart disease. Responses for all studies were coded as 0 (not diagnosed/treated) and 1 (diagnosed/treated).

#### Covariates

For all regression models, we control for age, sex, education, and race, when appropriate. For example, SWAN was a study of all women, so sex was not included. Sex was coded as male (0) and female (1). Age was centered at 50 and divided by 10, to improve convergence. Race was coded as white (0) and non-white (1). Education was often assessed as the number of years in school, although some studies employed a shorter Likert scale.

### Analyses

#### Cross-sectional

First, for each dataset, we calculated the bivariate relationships between purpose and health outcomes (self-rated health, smoking status, and heart condition status) among adults with diabetes. Second, we used regression — least squares when the outcome was self-rated health and binary logistic when the outcome was smoking status or heart condition — to calculate the relationship between purpose and the health outcomes controlling for participant age, sex, education, and race. We then used meta-analysis to examine the average purpose coefficient across all datasets and to estimate the heterogeneity of that coefficient across samples. In the meta-analysis, coefficients are weighted by the number of participants used in the calculation of the estimate. Cross-sectional analyses were preregistered on OSF (osf.io/dr862). We deviated from the preregistration by adding several datasets — ALSA, KGSS, MIDJA, and SWAN — that were not identified during the original search. We additionally deviated by dropping either sex or race as a covariate when a study was homogeneous on that variable. In addition, we preregistered bivariate analyses (available in Supplemental File 1, Tables 3–5), which correspond with our overall findings.

#### Longitudinal

To examine the relationship between purpose and change in health among adults with diabetes, we first estimated change in each health outcome (self-rated health, smoking status, and heart condition status) using multilevel growth models. Time was measured in years, centered at the individual’s last wave of participation in a study. We chose to center at the end of the study, because centering at baseline was redundant with the cross-sectional analyses. By examining the last time point, we estimate the long-term differences in health as a function of purpose and diabetes status. We included purpose, age, sex, race, and education as predictors of intercept and slope in these growth models. As with the cross-sectional analyses, we then used meta-analysis to estimate the overall relationship between sense of purpose and the intercept (i.e., final measurement of health) and slope (change in health) for each outcome of interest. We modeled the outcome as a continuous variable when it was self-rated health and dichotomous — using a binary logistic multilevel model — when the outcome was smoking or heart condition status. Again, we also examined the heterogeneity of these coefficients across sample. Coefficients are weighted by the number of participants, not the total number of measurements, used in the calculation of the estimate. Longitudinal analyses were preregistered after completion of cross-sectional analyses (https://osf.io/t6phe). We deviated from the preregistration and omitted the KGSS study from the heart condition analyses, as the base rates of heart conditions were too low in this data set and the analytic models could not converge. As exploratory analyses (not preregistered), we also tested quadratic growth curves, in the event that participants initially increased and then decreased in self-rated health (or vice versa) or quit then resumed smoking.

## Results

### Cross-Sectional Analyses

First, we examined the bivariate relationships between purpose and health outcomes among adults with diabetes. Sense of purpose was positively correlated with self-rated health in all studies except the CLOC and the STRIDE. Sense of purpose was higher for non-smokers than for smokers only in the HRS and the USNHMS, and purpose was higher for individuals without heart conditions than for individuals with heart conditions only in the HRS, the USNHMS, and the WLS. All bivariate relationships among adults with diabetes are shown in Table 1.

**Table 1:** Bivariate associations and mean comparisons for purpose and health outcomes among diabetic adults

	**Self-rated health**		**Smoking**						**Heart condition**					
			**Yes**		**No**		**Difference**		**Yes**		**No**		**Difference**	
**Study**	***r***	***p***	***N***	**M (SD)**	***N***	**M (SD)**	***d***	***p***	***N***	**M (SD)**	***N***	**M (SD)**	***d***	***p***
ACL	0.15	.020	24	4.22 (1.03)	204	4.22 (1.17)	0.00	.500	26	4.23 (1.14)	201	4.22 (1.16)	-0.01	.478
ALSA	0.21	.012	9	3.19 (0.94)	136	3.34 (0.90)	0.16	.319						
CLOC	− 0.03	.892	1	4.73 (0.00)	26	3.66 (0.82)			4	3.27 (1.16)	23	3.78 (0.76)		
ELSA	0.39	.043	5	3.88 (0.26)	22	3.72 (0.65)	− 0.25	.307	7	3.37 (0.67)	20	3.89 (0.52)	0.93	.026
HRS	0.27	< .001	547	3.63 (0.82)	3586	3.73 (0.78)	0.12	.004	1375	3.60 (0.79)	2838	3.77 (0.78)	0.22	< .001
KGSS	0.35	.003							18	3.44 (1.65)	54	3.76 (1.33)	0.22	.209
MIDJA	0.35	.004	23	3.13 (0.58)	35	3.37 (0.65)	0.38	.086	17	3.43 (0.53)	46	3.30 (0.64)	-0.22	.219
MIDUS	0.20	< .001	49	3.65 (0.82)	130	3.76 (0.86)	0.13	.227	29	3.54 (0.97)	279	3.76 (0.86)	0.25	.103
STRIDE	0.32	.213												
SWAN	0.10	.002	228	4.66 (0.67)	784	4.71 (0.68)	0.07	.184	115	4.68 (0.63)	897	4.70 (0.68)	0.03	.385
USNHMS	0.27	< .001	116	3.65 (0.87)	265	3.94 (0.70)	0.40	< .001	180	3.70 (0.75)	535	3.91 (0.77)	0.27	.001
WLS	0.30	< .001	66	3.81 (0.92)	370	3.87 (0.75)	0.08	.270	95	3.57 (0.83)	342	3.95 (0.75)	0.49	< .001

**Fig. 1:**
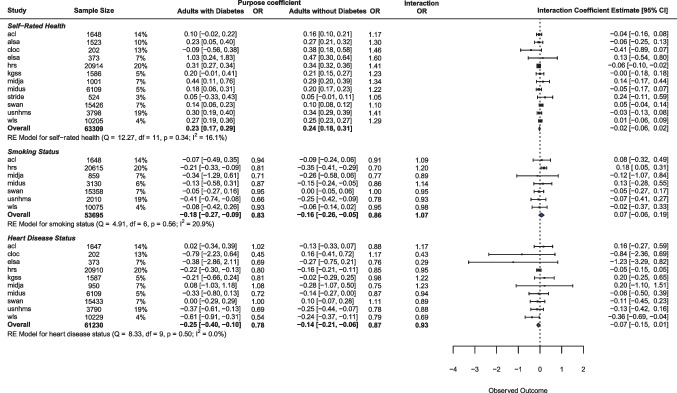
Forest plot of cross-sectional regression models. Columns for purpose coefficient are from models without interactions. All models control for age, education, sex, and race

We examined the relationship between purpose and health outcomes among adults with diabetes, controlling for age, sex, education, and race. The estimates of the relationship between purpose and outcomes from the sub-sample regressions can be seen in Fig. 1.

After testing these relationships within each study, we examined the overall association using meta-analytic tools. That is, we calculated the weighted average effect, with standard errors accounting for the combined sample sizes. We also estimate between-study heterogeneity, as differences between studies may reflect moderators of the effects. For each meta-analysis, we report the overall weighted effect and the heterogeneity of the effects. If the heterogeneity is significant, we also report which, if any, of the following variables explained this heterogeneity: mean age of participants, the earliest year of data collection, the number of items used to measure sense of purpose, and whether the study was run in the USA.

#### Self-Rated Health

Among adults with diabetes, purpose was positively related to self-rated health overall (*b* = 0.23, *p* < .001) and in a majority of the studies: ALSA (*b* = 0.23, *p* = .011), ELSA (*b* = 1.03, *p* = .019), HRS (*b* = 0.31, *p* < .001), MIDJA (*b* = 0.44, *p* = .011), MIDUS (*b* = 0.18, *p* = .005), SWAN (*b* = 0.14, *p* = .001), and USNHMS (*b* = 0.30, *p* < .001). The heterogeneity between effect sizes was significant (*I*^2^ = 58.29%, *Q* = 31.62, *df* = 11, *p* = .001). This heterogeneity was partially related to the number of purpose items used; for each additional item, the effect size increased by 0.03 (*p* < .001).

#### Smoking Status

Among adults with diabetes, sense of purpose was associated with a lower likelihood of being a smoker only in the HRS (*b* =  − 0.21, OR = 0.81, *p* < .001) and the USNHMS (*b* =  − 0.41, OR = 0.66, *p* = .015). Overall, purpose was associated with lower rates of smoking among adults with diabetes (*b* =  − 0.18, OR = 0.83, *p* < .001). The heterogeneity between effect sizes was nonsignificant (*I*^2^ = 0.00%, *Q* = 4.31, *df* = 6, *p* = .635). Given this lack of heterogeneity, combined with the lack of significance in the majority of the studies, we expect that testing this hypothesis in one study alone would likely yield a non-significant result. By using a coordinated analysis approach, we are able to detect this effect and find little evidence that differences between the studies contribute to any variation in effect size.

#### Heart Condition

Among adults with diabetes, purpose in life was associated with lower likelihood of having a heart condition overall (*b* =  − 0.25, OR = 0.78, *p* = .001) and in the HRS (*b* =  − 0.22, OR = 0.80, *p* =  < .001), the USNHMS (*b* =  − 0.37, OR = 0.69, *p* = .003), and the WLS (*b* =  − 0.61, OR = 0.54, *p* < .001). The heterogeneity between effect sizes was nonsignificant (*I*^2^= 45.72%, *Q* = 12.84, *df* = 9, *p* = .170).

### Longitudinal Analyses

Growth models were conducted with time, in years, centered on each participant’s final wave of health data. Intercepts are thus interpreted as the predicted value of the health outcome at their final wave, and the purpose coefficients estimate the prospective relationship between the first measurement of purpose and the final assessment of health. The weighted average estimate of the intercepts and the relationship of purpose can be found in Table 2. Across all three outcomes, sense of purpose was prospectively associated with the outcome in the HRS, although rarely in other studies, likely due to the greater power in the HRS study. The meta-analytic estimate of this relationship was always significant.

**Table 2:** Summary of prospective meta-analysis models

					**95% confidence interval**	
**Coefficient**	***b***	**SE**_**b**_	***Z***	***p***	**Lower**	**Upper**
**Self-rated health**						
Average intercept	2.20	0.15	14.37	< .001	1.90	2.50
Effect of purpose on intercept	0.17	0.04	4.34	< .001	0.10	0.25
Average slope	− 0.02	0.00	− 3.75	< .001	− 0.02	− 0.01
Effect of purpose on slope	0.00	0.00	− 1.80	.072	− 0.01	0.00
**Smoking**						
Average intercept	− 3.72	3.14	− 1.19	.235	− 9.87	2.42
Effect of purpose on intercept	− 0.21	0.09	− 2.32	.020	− 0.38	− 0.03
Average slope	− 0.18	0.43	− 0.41	.679	− 1.02	0.67
Effect of purpose on slope	− 0.01	0.04	− 0.13	.900	− 0.09	0.08
**Heart disease**						
Average intercept	− 1.99	1.33	− 1.49	.135	− 4.61	0.62
Effect of purpose on intercept	− 0.23	0.09	− 2.50	.012	− 0.41	− 0.05
Average slope	0.18	0.24	0.77	.442	− 0.28	0.65
Effect of purpose on slope	0.02	0.01	1.46	.144	− 0.01	0.05

Purpose was significantly prospectively associated with self-rated health in the ALSA (*b* = 0.27, *p* = .028) and the HRS (*b* = 0.27, *p* < .001), but not in any additional datasets. The meta-analytic estimate of the purpose coefficient was significant (*b* = 0.17, *p* < .001), and there was significant heterogeneity between studies (*I*^2^ = 42.64%, *Q* = 17.11, *df* = 8, *p* = .029). None of the examined study moderators — age at baseline, year of baseline, length of longitudinal study, number of purpose items, or US versus non-US — was associated with this variability.

Purpose was significantly prospectively associated with smoking status only in the HRS (*b* =  − .19, *p* = .034); the meta-analytic estimate of the purpose coefficient was significant (*b* =  − 0.21, *p* = .020). There was not significant heterogeneity in the effect sizes between studies (*I*^2^ = 0.00%, *Q* = 0.46, *df* = 6, *p* = .998).

Purpose was significantly prospectively associated with heart condition status in the HRS ($b$= − 0.29, *p* = .007); the meta-analytic estimate of the purpose coefficient was significant (*b* =  − 0.23, *p* = .012). Again, the variability in effect sizes between studies was nonsignificant (*b* =  − 0.23, 95%CI [− 0.41, − 0.05]), and there was no significant heterogeneity between studies (*I*^2^ = 0.00%, *Q* = 6.34, *df* = 6, *p* = .386). It should be noted that when the HRS was removed from analyses, the meta-analytic effect was still statistically significant and in the same direction for self-rated health (*b* = 0.13, *p* = .001), but not for smoking status (*b* =  − 0.38, *p* = .304) or heart condition status (*b* =  − 0.07, *p* = .687).

#### Change in Health

Sense of purpose was associated with less rapid increases in self-rated health in the ELSA (*b* =  − 0.17, *p* = .001) and the KGSS (*b* =  − 0.15, *p* = .041); however, the overall effect of purpose on changes in self-rated health was non-significant (*b* = 0.00, p = .072), although there was significant heterogeneity between studies (*I*^2^ = 7.31%, *Q* = 19.69, *df* = 8, *p* = .012). Examination of the between-study moderators suggested that American studies estimated null relationships between purpose and change in self-rated health, while non-American studies showed a negative relationship (*difference*_b_ = 0.11, *difference*_p_ < .001). No other moderators were significantly associated with variability between studies.

Purpose was not associated with changes in smoking status in any study, nor was the overall effect statistically significant (*b* =  − 0.01, *p* = .900). Similarly, purpose was unassociated with changes in heart condition status over time in any study, and the meta-analytic effect was nonsignificant (*b* = 0.02, *p* = .145). There was nonsignificant heterogeneity in effect sizes in both the smoking (*I*^2^ = 0.00%, *Q* = 0.73, *df* = 6, *p* = .994) and heart disease meta-analyses (*I*^2^ = 0.00%, *Q* = 4.13, *df* = 6, *p* = .659).

For no study or outcome was the quadratic term in the quadratic growth model statistically significant — that is, we have no evidence that self-rated health decreases and then increases, nor is there evidence that participants quit and then resume smoking (or develop and then recover from a heart condition).

## Discussion

The current study found that sense of purpose is concurrently associated with self-rated health, smoking status, and heart conditions among adults with diabetes. Moreover, sense of purpose was prospectively related to future self-rated health. Some of these associations replicated across multiple data sets. For example, the relationship between sense of purpose and self-rated health was significant in 8 of 12 data sets. For smoking and cardiovascular outcomes, these were less consistently significant across data sets. However, even when results failed to reach statistical significance in single studies, effect sizes were nearly always in the same direction and of similar magnitude, suggesting that lack of power drove “non-replications.” While purpose in life is associated with the levels of self-rated health and other health indicators, purpose appears to be unassociated with changes in health status over time.

These findings point generally to the promise of integrating sense of purpose into care for adults with diabetes. The current study replicates past work with general adult samples, which have linked sense of purpose to self-rated health [12, 13] and cardiovascular disease risk [8] and extends these findings to demonstrate they largely hold within this patient subsample. Moreover, the current findings suggest that, at least cross-sectionally, sense of purpose may predict smoking status among adults with diabetes, an association that has received mixed evidence in other general samples [17]. Given sense of purpose has been linked to adherence behaviors associated with diabetes prevention and management [19], and reduced blood glucose levels [3, 4], physicians would benefit from assessing patients’ sense of purpose to identify which individuals are at greater risk for later health consequences, possibly as a result of poor diabetes self-care. Such assessments can be administered using only a few items, perhaps in intake inventories. One clear direction for future research is to examine whether the associations between sense of purpose and health are explained through unique mediators in this sample, such as getting regular checkups and care for diabetes.

However, it is worth noting the heterogeneity found with respect to the magnitude of associations across datasets. For instance, the relationship between purpose and self-rated health in adults with diabetes varied across studies to a noteworthy degree. There was no consistent explanation for this variation: in the cross-sectional analyses, this variation was associated with length of purpose scales, while in the longitudinal analyses, variation was associated with country of origin. One possible interpretation comes with respect to the role of cultural norms, both with respect to the prevalence and discussion of diabetes and the interpretation of the purpose items. It may be that more information and opportunities are available for diabetes maintenance in the USA given it is a more common phenomenon, which may lead to more routes by which purposeful adults are able to lead healthier lifestyles. Future research should not only consider study-level demographics as moderators, but other methods of quantifying cultural norms.

Sense of purpose was unassociated with changes in health over time. This may be due to a methodological issue. Changes in behavior and health perceptions among adults with diabetes may occur most dramatically or with the greatest likelihood just following a diagnosis. If this is true, then greater declines in self-rated health and smoking should be seen in the months following a diagnosis. Moreover, if the role of sense of purpose operates through self-care and condition management, these effects may be expected to occur around diagnosis. Few of the data sets used in the current study provided date of diagnosis, so we were unable to track time since diagnosis as a timescale in the longitudinal analyses. Had we been able to select for participants newly diagnosed or better incorporate time since diagnosis into our analyses, we might have seen relationships between purpose and self-rated health or smoking. However, it would also be expected that the development of heart conditions would occur farther out, perhaps years or decades post-diagnosis, and so might be just as easily captured by our models. We cannot ignore the likely possibility that sense of purpose is simply unassociated with changes in health.

The limitations of this study naturally limit the generalizability of our conclusions. First, all studies used self-report questionnaires for all variables in this study. The use of self-report is common in these panel studies and often allows for larger and more representative samples (a clear advantage for these analyses). However, especially in the context of health, associations between psychological constructs and real-world behavior or objective outcomes should be documented before changing policy. This issue is exemplified in the decades-old discussion regarding what self-rated health actually captures, given it is both a subjective measure, wherein participant ratings may be based on different reference groups and yet provides valuable prediction of later outcomes, including mortality [39–41]. Second, the use of coordinated analysis requires some harmonization of measures, if studies are to be compared. To this end, we often dichotomized outcomes (smoking or heart condition) or covariates to facilitate comparisons. However, this necessarily means that in some studies, information and specificity were lost. Third, given the lack of available information across studies, we were unable to compare type I and type II diabetes, which is a critical prior to inclusion in personalized medicine.

## Conclusions

These caveats aside, the current work shows that sense of purpose is concurrently and prospectively associated with health outcomes and behaviors among adults with diabetes. Sense of purpose is likely an important psychological factor in the health of adults with diabetes and may have utility in designing interventions to combat the negative outcomes of diabetes. Future research should consider the role of purpose in identifying which adults with diabetes are in most of need of intervention or alternatively who may expect to maintain positive health outcomes in the years ahead.

### Supplementary Information

Below is the link to the electronic supplementary material.

Supplementary file1 (PDF 334 KB)

Supplementary file2 (PDF 336 KB)

## Data Availability

For information on how to access the employed datasets, interested readers can visit the websites dedicated to each individual study.
