# Supplementary material for: Associations for Sense of Purpose with Smoking and Health Outcomes Among Adults with Diabetes
Source: Int J Behav Med. 2023 Jul 6;31(4):538–48. doi: 10.1007/s12529-023-10191-0 (PMC11269333; doi:10.1007/s12529-023-10191-0)
Supplement: Supplementary file 1 — Supplementary file1 (PDF 334 KB) [file 12529_2023_10191_MOESM1_ESM.pdf]

Supplementary Material: Code and Analysis for Study 1

This document contains all code used to clean and analyze data for Study 1. Results of analyses are saved as R objects and imported into the manuscript.

Contents

1 Data cleaning **2**
- 1.1 ACL . . . 2
- 1.2 ALSA . . . 3
- 1.3 CLOC . . . 3
- 1.4 ELSA . . . 4
- 1.5 HRS . . . 5
- 1.6 KGSS . . . 8
- 1.7 MIDJA . . . 9
- 1.8 MIDUS . . . 10
- 1.9 STRIDE . . . 11
- 1.10 SWAN . . . 12
- 1.11 USNHMS . . . 13
- 1.12 WLS . . . 14

2 Analyze individual studies **15**
- 2.1 Code for all studies . . . 15
- 2.2 Source script on all studies . . . 20

3 Descriptive Statistics **22**
- 3.1 Mean, standard deviation, min, max, α . . . 22
- 3.2 Cross-tabs . . . 28

4 Bivariate effect sizes **31**
- 4.1 Correlation between purpose and self-rated health . . . 31
- 4.2 Standardized difference in purpose between smokers and non-smokers . . . 32
- 4.3 Standardized difference in purpose between individuals with heart disease and individuals without . . . 33

5 Meta-analysis: Interaction **35**
- 5.1 Self-rated health . . . 35
- 5.2 Smoking status . . . 38
- 5.3 Heart Disease . . . 40
- 5.4 Summarize meta analyses . . . 43
- 5.5 Plot . . . 49

6 Moderators of interaction **53**

7 Measures of purpose **57**

8 Session Information **60**

1 Data cleaning

1.1 ACL

```
load(here("Study 1/raw data/ACL/04690-0001-Data.rda"))
acl <- da04690.0001

acl <- acl %>%
  dplyr::select(V1 = V1,
         purpose = V12561,
         diabetes = V12286,
         srh = V12236,
         smoker = V13003,
         heart = V12297,
         gender = V1801,
         white = V2059,
         edu1 = V2046,
         edu2 = V2047,
         edu3 = V2048,
         edu4 = V2049,
         edu5 = V2050,
         birthyr = V1645,
         interviewyr = V12005)

#make variables numeric
acl$purpose <- as.numeric(acl$purpose)
acl$diabetes <- as.numeric(acl$diabetes)
acl$srh <- as.numeric(acl$srh)
acl$smoker <- as.numeric(acl$smoker)
acl$heart <- as.numeric(acl$heart)
acl$gender <- as.numeric(acl$gender)

# reverse code purpose and self-rated health
acl$purpose <- acl$purpose*-1 + 5
acl$srh <- acl$srh*-1 + 6

# change 1 (yes) to 0 and 2 (no) to 1
acl <- scrub(acl,
             where = c("diabetes","smoker","heart"),
             isvalue = 2, newvalue = 0)

# make gender 0 = male 1 = female
acl$gender <- acl$gender - 1

#reverse race variable
acl$race = ifelse(acl$white == 1, 0, 1)
#create single education variable
acl$edu = ifelse(acl$edu1 == 1, 8, NA)
acl$edu = ifelse(acl$edu2 == 1, 10, acl$edu)
acl$edu = ifelse(acl$edu3 == 1, 12, acl$edu)
acl$edu = ifelse(acl$edu4 == 1, 14, acl$edu)
acl$edu = ifelse(acl$edu5 == 1, 16, acl$edu)

#calculate age
acl$age = acl$interviewyr - acl$birthyr
```

```
save(acl, file=here("Study 1/created data/acl.Rdata"))
```

1.2 ALSA

```
#load raw data
alsa1 <- read_por(here("Study 1/raw data/ALSA/06707-0003-Data.por"))
alsa <- read_por(here("Study 1/raw data/ALSA/06707-0007-Data.por"))
alsa = full_join(alsa, alsa1); rm(alsa1)

#dplyr::select variables in main data set

alsa <- alsa %>%
  dplyr::select(SEQNUM = SEQNUM,
         birthday = BIRTHDAT,
         gender = SEXW3,
         edu = FORMSCW3,
         purpose1 = DIRPURW3,
         purpose2 = GOALWSW3,
         purpose3 = FUTPLNW3,
         srh = SRHW3,
         diabetes1 = DIABETE,
         diabetes3 = DIABW3,
         smoker = SMOKERW3)

alsa$birthyear = substr(alsa$birthday, nchar(alsa$birthday)-4+1, nchar(alsa$birthday))
# there's one that doesn't make sense. remove
alsa <- alsa %>%
  mutate(birthyear = ifelse(birthyear == "9100", NA, birthyear),
         birthyear = as.numeric(birthyear),
         age = 1994-birthyear)

alsa = alsa %>%
  mutate(gender = gender-1, #make binary, 1 = female, 0 = male
         srh = srh*-1 + 6, # reverse code so 5 = excellent, 1 = poor
         diabetes1 = ifelse(diabetes1 == 1, 1, 0), #recode so that no is 0
         diabetes3 = ifelse(diabetes3 == 1, 1, 0), #recode so that no is 0
         smoker = ifelse(smoker == 1, 1, 0)) #recode so that no is 0


# use information from two diabetes variables to create most complete and up-to-date diabetes status variable
alsa$diabetes = alsa$diabetes1
alsa$diabetes[alsa$diabetes3 == 1] = 1

alsa = dplyr::select(alsa, -diabetes1, - diabetes3)

save(alsa, file=here("Study 1/created data/alsa.Rdata"))
```

1.3 CLOC

```
cloc <- read_sav(here("Study 1/raw data/CLOC/CLOCdata.sav"))

cloc <- cloc %>%
```

```r
  dplyr::select(id = V1,
         purpose1 = V9522,
         purpose2 = V9523,
         purpose3 = V9524,
         purpose4 = V9525,
         purpose5 = V9526,
         diabetes = V8006,
         srh = V8312,
         smoker = V8330,
         heart = V8005,
         gender = V535,
         race = V438,
         edu = V455,
         birthyr = V454,
         interviewyr = V7015)

#remove values that mean missing or don't know
cloc <- cloc %>%
  mutate(
    across(matches("^purpose\\d$"), ~ifelse(. > 4, NA, .)),
    #remove 8 (don't know)
    heart = ifelse(heart == 8, NA, heart),
    # change 5 (no) to 0
    across(c("diabetes","smoker","heart"), ~ifelse(. == 5, 0, .)))

#reverse purpose items
purpose.items <- cloc[,which(grepl("purpose",names(cloc)))]
purpose.items <- reverse.code(keys = rep(-1, ncol(purpose.items)),
                             items = purpose.items)
cloc[,which(grepl("purpose",names(cloc)))] <- purpose.items

# reverse code self-rated health
cloc$srh <- cloc$srh*-1 + 6

# make gender 0 = male 1 = female
cloc$gender <- cloc$gender - 1

#create single race variable
cloc$race = ifelse(cloc$race == 1, 0, 1)

#calculate age
cloc$age = cloc$interviewyr - cloc$birthyr

save(cloc, file=here("Study 1/created data/cloc.Rdata"))
```

1.4 ELSA

```r
#load raw data
h_elsa <- read_sav(here("Study 1/raw data/ELSA/h_elsa.sav"))
ryff_elsa <- read_sas(here("Study 1/raw data/ELSA/wave_2_ryff_data.sas7bdat"))

#dplyr::select and rename variables in each set
h_elsa <- h_elsa %>%
  dplyr::select(idauniq = idauniq,
```

```
         diabetes = r2diabe,
         srh = r2shlt,
         smoker = r2smoken,
         heart = r2hearte,
         gender = ragender,
         age = r2agey,
         race = raracem,
         edu = raeduc_e)

elsa <- ryff_elsa %>%
  dplyr::select(idauniq = idauniq,
         purpose1 = RFWAND,
         purpose2 = RFONEDA,
         purpose3 = RFDONE,
         purpose4 = RFSENSE,
         purpose5 = RFACCOM,
         purpose6 = RFPLANS,
         purpose7 = RFACTIV) %>%
# merge together
  inner_join(h_elsa)

#remove "don't know"
elsa <- elsa %>%
  mutate(across(everything(), ~ifelse(. == -1, NA, .)))

#reverse purpose items
purpose.items <- elsa[,which(grepl("purpose",names(elsa)))]
purpose.items <- reverse.code(keys = rep(-1, ncol(purpose.items)),
                              items = purpose.items)
elsa[,which(grepl("purpose",names(elsa)))] <- purpose.items

#reverse self rated health item
elsa$srh <- elsa$srh*-1 + 6

# make gender 0 = male 1 = female
elsa$gender <- elsa$gender - 1

# make race 0 = white, 1 = nonwhite
elsa$race <- ifelse(elsa$race == 1, 0, 1)

# make education 1-4 instead of 1, 3, 4, 5
elsa$edu <- ifelse(elsa$edu == 1, 1, elsa$edu-1)

#make diabetes numeric
elsa$diabetes <- as.numeric(elsa$diabetes)

save(elsa, file=here("Study 1/created data/elsa.Rdata"))
```

1.5 HRS

The HRS was the only study to measure purpose at different times for different participants. Consequently, additional care must be taken to match the first time a participant completed the purpose questionnaire with his or her health data.

```r
#load raw data
hrs <- read_sas(here("Study 1/raw data/HRS/rndhrs_p.sas7bdat"))
h06data <- read_sas(here("Study 1/raw data/HRS/h06f2b.sas7bdat"))
h08data <- read_sas(here("Study 1/raw data/HRS/h08f2a.sas7bdat"))
h10data <- read_sas(here("Study 1/raw data/HRS/hd10f5c.sas7bdat"))
h12data <- read_sas(here("Study 1/raw data/HRS/h12f1a.sas7bdat"))
h14data <- read_sas(here("Study 1/raw data/HRS/h14e1a.sas7bdat"))

#dplyr::select variables in main data set

hrs <- hrs %>%
  dplyr::select(HHIDPN = HHIDPN,
         birthyear = RABYEAR,
         gender = RAGENDER,
         race = RARACEM,
         edu = RAEDYRS,
         srh_06 = R8SHLT,
         srh_08 = R9SHLT,
         srh_10 = R10SHLT,
         srh_12 = R11SHLT,
         srh_14 = R12SHLT,
         diabetes_06 = R8DIABE,
         diabetes_08 = R9DIABE,
         diabetes_10 = R10DIABE,
         diabetes_12 = R11DIABE,
         diabetes_14 = R12DIABE,
         smoker_06 = R8SMOKEN,
         smoker_08 = R9SMOKEN,
         smoker_10 = R10SMOKEN,
         smoker_12 = R11SMOKEN,
         smoker_14 = R12SMOKEN,
         heart_06 = R8HEARTE,
         heart_08 = R9HEARTE,
         heart_10 = R10HEARTE,
         heart_12 = R11HEARTE,
         heart_14 = R12HEARTE)

# dplyr::select variables in yearly data sets,
# filter out participants without data,
# rename variables

h06data <- h06data %>%
  dplyr::select(HHIDPN = HHIDPN, # id
         purpose1_06 = KLB035A,
         purpose2_06 = KLB035B,
         purpose3_06 = KLB035C,
         purpose4_06 = KLB035D,
         purpose5_06 = KLB035E,
         purpose6_06 = KLB035F,
         purpose7_06 = KLB035G) %>%
  mutate(n_respond = rowSums(!is.na(.))) %>%
  filter(n_respond > 1) %>%
  dplyr::select(-n_respond)

names(h08data) <- toupper(names(h08data))
```

```r
h08data <- h08data %>%
  dplyr::select(HHIDPN = HHIDPN, # id
         purpose1_08 = LLB035A,
         purpose2_08 = LLB035B,
         purpose3_08 = LLB035C,
         purpose4_08 = LLB035D,
         purpose5_08 = LLB035E,
         purpose6_08 = LLB035F,
         purpose7_08 = LLB035G) %>%
mutate(n_respond = rowSums(!is.na(.))) %>%
  filter(n_respond > 1) %>%
  dplyr::select(-n_respond)


h10data <- h10data %>%
  dplyr::select(HHIDPN = HHIDPN, # id
         purpose1_10 = MLB035A,
         purpose2_10 = MLB035B,
         purpose3_10 = MLB035C,
         purpose4_10 = MLB035D,
         purpose5_10 = MLB035E,
         purpose6_10 = MLB035F,
         purpose7_10 = MLB035G) %>% #purpose 7
mutate(n_respond = rowSums(!is.na(.))) %>%
  filter(n_respond > 1) %>%
  dplyr::select(-n_respond)

h12data <- h12data %>%
  dplyr::select(HHIDPN, # id
         purpose1_12 = NLB035A,
         purpose2_12 = NLB035B,
         purpose3_12 = NLB035C,
         purpose4_12 = NLB035D,
         purpose5_12 = NLB035E,
         purpose6_12 = NLB035F,
         purpose7_12 = NLB035G) %>%
mutate(n_respond = rowSums(!is.na(.))) %>%
  filter(n_respond > 1) %>%
  dplyr::select(-n_respond)

h14data <- h14data %>%
  dplyr::select(purpose1_14 = OLB033A,
         purpose2_14 = OLB033B,
         purpose3_14 = OLB033C,
         purpose4_14 = OLB033D,
         purpose5_14 = OLB033E,
         purpose6_14 = OLB033F,
         purpose7_14 = OLB033G)
mutate(n_respond = rowSums(!is.na(.))) %>%
  filter(n_respond > 1) %>%
  dplyr::select(-n_respond)

# merge data sets,
hrs <- hrs %>%
  left_join(h06data) %>%
  left_join(h08data) %>%
```

```r
  left_join(h10data) %>%
  left_join(h12data) %>%
  left_join(h14data) %>%

  # gather into long form, with each purpose response for each
  # participant at each year having its own row
  gather(key="variable",value="value",
         which(grepl("purpose", names(.)))) %>%

  #remove rows with missing purpose values
  filter(!is.na(value)) %>%

  #separate the purpose variable into two columns, one with the variable and one with the year
  separate(col = "variable", into = c("variable","pyear")) %>%

  # spread into a different long form, with each year for each participant having its own row
  spread(key="variable", value="value") %>%

  # group by participant, and then for each person,
  # dplyr::select only the row with the smallest (first) year
  group_by(HHIDPN) %>%
  filter(pyear == min(pyear)) %>%
  ungroup() %>%

  # gather the remaining repeated values (i.e., have an underscore) into a long form with
  # each response on each variable for each participant at each year having its own row
  gather(key="variable",value="value", which(grepl("_",names(.))), convert=T) %>%

  #remove missing values
  filter(!is.na(value)) %>%

  # separate the variable column into two, one with the variable and one with the year
  separate(col = "variable", into = c("variable","year")) %>%

  # spread back into long form with each year for each participant having its own row
  # note: purpose responses from the first year will be repeated across rows
  spread(key="variable", value="value") %>%

  #filter such that the year kept for each person matches the year of the purpose assessment
  filter(pyear == year) %>%
  ungroup() %>%

  #remove that purpose year variable
  dplyr::select(-pyear) %>%

  # fix variables to be consistent with other studies
  mutate(age = as.numeric(year)+2000 - birthyear,
         gender = gender - 1,
         race = ifelse(race == 1, 0, 1),
         srh = srh*-1 + 6)

save(hrs, file=here("Study 1/created data/hrs.Rdata"))
```

1.6 KGSS

```r
#load raw data
load(here("Study 1/raw data/KGSS/34665-0001-Data.rda"))
kgss = da34665.0001; rm(da34665.0001)

#dplyr::select variables in main data set

kgss <- kgss %>%
  dplyr::select(RESPID = RESPID,
         age = AGE,
         gender = SEX,
         edu = EDUC,
         purpose1 = DOMOREWK,
         purpose2 = RSNTOLIV,
         purpose3 = HAVEPLAN,
         purpose4 = LIFCNTRL,
         srh = HEALTH,
         diabetes = DIABETES,
         heart = HEARTPRB) %>%
  #make numeric
  mutate(gender = as.numeric(gender),
         edu= as.numeric(edu),
         purpose1= as.numeric(purpose1),
         purpose2= as.numeric(purpose2),
         purpose3= as.numeric(purpose3),
         purpose4= as.numeric(purpose4),
         srh= as.numeric(srh),
         diabetes= as.numeric(diabetes),
         heart= as.numeric(heart)) %>%
  # make binary varibales 0, 1
  mutate(gender = gender - 1,
         purpose1 = ifelse(purpose1 == 1, 1, 0),
         purpose2 = ifelse(purpose2 == 1, 1, 0),
         purpose3 = ifelse(purpose3 == 1, 1, 0),
         purpose4 = ifelse(purpose4 == 1, 1, 0),
         diabetes = ifelse(diabetes == 1, 1, 0),
         heart = ifelse(heart == 1, 1, 0),
         #reverse score srh
         srh = srh*-1 + 6,
         # remove "other" from education
         edu = ifelse(edu < 9, edu, NA))

save(kgss, file=here("Study 1/created data/kgss.Rdata"))
```

1.7 MIDJA

```r
#load raw data
load(here("Study 1/raw data/MIDJA/30822-0001-Data.rda"))
midja = da30822.0001; rm(da30822.0001)

#dplyr::select variables in main data set

midja <- midja %>%
  dplyr::select(MIDJA_IDS = MIDJA_IDS,  # id
         age = J1SQ2AGE,
```

```
         gender = J1SQ1,
         edu = J1SQ3,
         purpose1 = J1SJ8E, # purpose in life
         purpose2 = J1SJ8K, # purpose in life
         purpose3 = J1SJ8Q, # purpose in life
         purpose4 = J1SJ8W, # purpose in life
         purpose5 = J1SJ8CC, # purpose in life
         purpose6 = J1SJ8NN, # purpose in life
         purpose7 = J1SJ8PP, # purpose in life
         srh = J1SA1,
         diabetes = J1SA8X,
         ever_smoker = J1SB1A,
         smoker = J1SB4,
         heart = J1SA9D) %>%
# make values numeric, not factor
  mutate(gender = as.numeric(gender),
         edu = as.numeric(edu),
         purpose1 = as.numeric(purpose1),
         purpose2 = as.numeric(purpose2),
         purpose3 = as.numeric(purpose3),
         purpose4 = as.numeric(purpose4),
         purpose5 = as.numeric(purpose5),
         purpose6 = as.numeric(purpose6),
         purpose7 = as.numeric(purpose7),
         srh = as.numeric(srh),
         diabetes = as.numeric(diabetes),
         ever_smoker = as.numeric(ever_smoker),
         smoker = as.numeric(smoker),
         heart = as.numeric(heart))

#remove missing values
midja = scrub(midja, where = "ever_smoker", isvalue = 3)

#make binary (0,1)
midja = midja %>%
  mutate(gender = gender - 1,
         diabetes = ifelse(diabetes == 1, 1, 0),
         ever_smoker = ifelse(ever_smoker == 1, 1, 0),
         smoker = ifelse(smoker == 1, 1, 0),
         heart = ifelse(heart == 1, 1, 0))

midja$smoker[midja$ever_smoker == 0 & is.na(midja$smoker)] = 0


save(midja, file=here("Study 1/created data/midja.Rdata"))
```

1.8 MIDUS

```
midus <- read_sav(here("Study 1/raw data/MIDUS/02760-0001-Data.sav"))

midus <- midus %>%
  dplyr::select(purpose1 = A1SF1C,
         purpose2 = A1SF1G,
         purpose3 = A1SF1J,
```

```r
         diabetes = A1SA9X,
         srh = A1PA4,
         smoker = A1PA43,
         heart = A1PA29,
         heart.highbp = A1PA29CC,
         heart.none = A1PA29CK,
         gender = A1PRSEX,
         age = A1PAGE_M2,
         race = A1SS7,
         edu = A1PB1)

#remove values that mean missing or don't know
midus <- midus %>%
  mutate(
    across(c("srh", "smoker", "heart", "heart.highbp","heart.none"),
           ~ifelse(. == 7, NA, .)),
    across(c("purpose1","purpose2","purpose3","diabetes", "race", "gender"),
           ~ifelse(. == 8, NA, .)),
    edu = ifelse(edu == 97, NA, edu),
    #recode values that mean "no" in binary questions to 0
    across(c("diabetes", "smoker", "heart","heart.highbp","heart.none"),
           ~ifelse(. == 2, 0, .)),
    #recode values that mean "inappropriate" because earlier question to 0
    across(c("smoker", "heart", "heart.highbp","heart.none"),
           ~ifelse(. == 9, 0, .))
  )

#reverse code purpose items
purpose.items <- midus[,which(grepl("purpose",names(midus)))]
purpose.items <- reverse.code(keys = rep(-1, ncol(purpose.items)),
                              items = purpose.items)
midus[,which(grepl("purpose",names(midus)))] <- purpose.items


#gender binary with 0 = male
midus$gender = midus$gender-1
#race binary with 0 = white
midus$race = ifelse(midus$race == 1, 0, 1)

#heart condition variable (don't include hypertension)
midus$heart[midus$heart.highbp == 1] = 0
midus$heart[midus$heart.none == 1] = 0


midus$diabetes = as.numeric(midus$diabetes)

save(midus, file=here("Study 1/created data/midus.Rdata"))
```

1.9 STRIDE

```r
#load raw data
load(here("Study 1/raw data/STRIDE/35525-0001-Data.rda"))
stride <- da35525.0001
```

```r
#dplyr::select and rename variables in each set
stride <- stride %>%
  dplyr::select(RID = RID,
         purpose = PWB_PIL,
         diabetes = HOI19,
         srh = SF12_AGG_PHYS,
         gender = GENDER,
         age = AGE,
         race = ETHNIC,
         edu_hsd = EDU_HSD,
         edu_sc = EDU_SC,
         edu_ba = EDU_BA)

#reverse purpose item
stride$purpose <- stride$purpose*-1 + 8

#reverse self rated health item
stride$srh <- stride$srh*-1 + 6

# make gender 0 = male 1 = female
stride$gender <- as.numeric(stride$gender) - 1

# make race 0 = white, 1 = nonwhite
stride$race <- ifelse(as.numeric(stride$race) == 1, 0, 1)

# make education and diabetes numeric
stride$edu = case_when(
                       stride$edu_ba == "(1) Yes" ~ 16,
                       stride$edu_sc == "(1) Yes" ~ 14,
                       stride$edu_hsd == "(0) > HS education" ~ 12,
                       stride$edu_hsd == "(1) < or = HS Diploma" ~ 5,
                       TRUE ~ NA_real_)

stride$diabetes <- as.numeric(stride$diabetes)

# make diabetes 0 = no diabetes 1 = diabetes
stride$diabetes <- stride$diabetes-1


#make age numeric - find mid-points for ranges, base end-point on next logical value in sequence
stride$age <- as.numeric(stride$age)
stride$age[stride$age == 1] <- 18
stride$age[stride$age == 2] <- 23
stride$age[stride$age == 3] <- 28
stride$age[stride$age == 4] <- 33
stride$age[stride$age == 5] <- 38
stride$age[stride$age == 6] <- 43
stride$age[stride$age == 7] <- 48
stride$age[stride$age == 8] <- 53

save(stride, file=here("Study 1/created data/stride.Rdata"))
```

1.10 SWAN

```r
#load raw data
swan = read_sav(here("Study 1/raw data/SWAN/04368-0001-Data.sav"))

#dplyr::select variables in main data set
swan <- swan %>%
  dplyr::select(ID = ID,  # id
         age = AGE,
         edu = DEGREE,
         race = ETHNIC,
         purpose1 = MISSION, # purpose in life
         purpose2 = FAITH, # purpose in life
         purpose3 = DIFFICU, # purpose in life
         srh = HEALTH,
         diabetes = DIABETE,
         smoker = SMOKER,
         heart = HEART)

#remove missing values
swan <- swan %>%
  mutate(across(everything(), ~ifelse(. < 0, NA, .)))

swan = swan %>%
  mutate(race = ifelse(race == 10, 0, 1),  #make race binary (1 = nonwhite, 0 = white)
         diabetes = ifelse(diabetes == 2, 1, 0), #make diabetes binary (1 = yes, 0 = no)
         srh = srh*-1 + 6, # reverse code self-rated health
         purpose1 = purpose1*-1 + 4,
         purpose2 = purpose2*-1 + 4,
         purpose3 = purpose3*-1 + 4,
         heart = ifelse(heart == 2, 1, 0)) # make heart binary

save(swan, file=here("Study 1/created data/swan.Rdata"))
```

1.11 USNHMS

```r
usnhms <- read.delim(here("Study 1/raw data/USNHMS/23263-0001-Data.tsv"))

# dplyr::select and rename variables

usnhms <- usnhms %>%
  dplyr::select(id = CASEID,
         purpose1 = PWBP1,
         purpose2 = PWBP2,
         purpose3 = PWBP3,
         purpose4 = PWBP4,
         purpose5 = PWBP5,
         purpose6 = PWBP6,
         purpose7 = PWBP7,
         purpose8 = PWBP8,
         diabetes = DIABETES,
         srh = SF1,
         smoker = SMOKE2,
         heart = CHD,
         gender = SEX,
         age = AGE,
```

```
         race = RACE,
         edu = EDUC)

#remove 'don't know'
usnhms <- scrub(usnhms, isvalue = -1)
#remove 'refused to answer'
usnhms <- scrub(usnhms, isvalue = -2)
#change 2 to 0 for binary items
usnhms <- scrub(usnhms, where = c("diabetes", "heart"), isvalue = 2, newvalue = 0)

#reverse purpose items
purpose.items <- usnhms[,which(grepl("purpose",names(usnhms)))]
purpose.items <- reverse.code(keys = rep(-1, ncol(purpose.items)),
                              items = purpose.items)
usnhms[,which(grepl("purpose",names(usnhms)))] <- purpose.items

#reverse self rated health item
usnhms$srh <- usnhms$srh*-1 + 6

# make smoker variable binary
usnhms$smoker <- ifelse(usnhms$smoker == 3, 0, 1)

# make gender 0 = male 1 = female
usnhms$gender <- usnhms$gender - 1

# make race variable binary
usnhms$race <- ifelse(usnhms$race == 1, 0, 1)

save(usnhms, file=here("Study 1/created data/usnhms.Rdata"))
```

1.12 WLS

```
wls <- read.csv(here("Study 1/raw data/WLS/WLS.csv"))

wls <- wls %>%
  dplyr::select(
    ID = idpub,
    purpose1_grd = mn039rer,
         purpose2_grd = mn040rer,
         purpose3_grd = mn041rer,
         purpose4_grd = mn042rer,
         purpose5_grd = mn043rer,
         purpose6_grd = mn044rer,
         purpose7_grd = mn045rer,
         purpose1_sib = np039rer,
         purpose2_sib = np040rer,
         purpose3_sib = np041rer,
         purpose4_sib = np042rer,
         purpose5_sib = np043rer,
         purpose6_sib = np044rer,
         purpose7_sib = np045rer,
         diabetes_grd = mx095rer,
         diabetes_sib = nx115rer,
         srh_grd = mx001rer,
```

```r
         srh_sib = nx001rer,
         smokerever_grd = mx012rer,
         smokerever_sib = nx038rer,
         smokernow_grd = mx013rer,
         smokernow_sib = nx039rec,
         heart_grd = mx099rer,
         heart_sib = nx119rer,
         gender_grd = sexrsp,
         gender_sib = ssbsex,
         age_grd = ra029re,
         age_sib = sa029re,
         edu_grd = rb003red,
         edu_sib = sb003red) %>%
  gather(key="variable", value="value", which(grepl("_", names(.)))) %>%
  separate(col = "variable", into=c("variable","cohort")) %>%
  spread(key="variable", value="value")

#remove values that mean missing or don't know
wls <- scrub(wls, min = 0)

#recode values that mean "no" in binary questions to 0
wls <- scrub(wls, where=c("diabetes", "smokerever", "smokernow", "heart"),
               isvalue = 2, newvalue = 0)

#reverse code purpose items
purpose.items <- wls[,which(grepl("purpose",names(wls)))]
purpose.items <- reverse.code(keys = rep(-1, ncol(purpose.items)),
                              items = purpose.items)
wls[,which(grepl("purpose",names(wls)))] <- purpose.items



#smoking binary with 0 = not a current smoker
wls$smoker <- ifelse(wls$smokerever == 0, 0, wls$smokernow)

#gender binary with 0 = male
wls$gender = wls$gender-1

save(wls, file=here("Study 1/created data/wls.Rdata"))
```

2 Analyze individual studies

2.1 Code for all studies

A single script file was written that could be sourced with each of the datasets in turn. The script was written in order to allow us to loop through each of the data sets in turn.

First, assume i refers to the name of a dataset. The cleaned data is loaded into R.

```r
load(paste0(here("Study 1/created data/"), i, ".Rdata"))

dataset = get(i)
```

Next, the purpose scale is scored and the alpha coefficient is extracted.

```r
# how many purpose items
purpose.items = length(names(dataset)[grepl("purpose", names(dataset))])

# harmonize scaling. All purpose items will be score on a scale from 0 to 5
min.p = min(dataset[,grepl("purpose", names(dataset))], na.rm=T)
max.p = max(dataset[,grepl("purpose", names(dataset))], na.rm=T)

if(purpose.items > 1){

  purpose.alpha = dataset %>%
  select_if(grepl("purpose", names(.))) %>%
    mutate(across(everything(), as.numeric)) %>%
  psych::alpha(check.keys = TRUE)

  keys = purpose.alpha$keys

  if(keys[1] != first.key){keys = keys*-1}

  # score dataset
  dataset$purpose = dataset %>%
    select_if(grepl("purpose", names(.))) %>%
    mutate(across(everything(), as.numeric)) %>%
    reverse.code(keys = keys, items = .) %>%
    rowMeans(na.rm=T)

  } else (purpose.alpha = NULL)
```

We record the total sample size of the study, then filter out participants who do not meet elligability criteria. Participants must have a measure of purpose, must have information regarding their diabetes status, must have information on at leeast one of the outcomes measures and must have all covariates. We record the new sample size.

```r
totalStudyN = nrow(dataset)

#identify which columns are in data set

# possible outcome columns
outcomes <- names(dataset)[which(names(dataset) %in% c("srh", "smoker", "heart"))]

# possible covariate columns
covariates <- names(dataset)[which(names(dataset) %in% c("age", "gender", "race", "edu"))]

# filter out participants without purpose, without at least one outcome
dataset = filter(dataset, !is.na(purpose))
dataset = filter(dataset, !is.na(diabetes))

if(length(outcomes) > 1){
  missing.outcomes = apply(X = dataset[,outcomes],
                           MARGIN = 1, FUN = function(x) sum(is.na(x)))
  dataset = dataset[missing.outcomes < length(outcomes), ]
  } else{
    dataset = filter(dataset, !is.na(outcomes))
  }

missing.covariates = apply(X = dataset[,covariates],
                           MARGIN = 1, FUN = function(x) sum(is.na(x)))
```

```r
dataset = dataset[missing.covariates == 0, ]
analysisSample = nrow(dataset)

# create diabetes only and non-diabetes only data sets

dataset.diab = filter(dataset, diabetes == 1)
dataset.nod = filter(dataset, diabetes == 0)
```

Additional datasets are created to represent only participants with diabetes and only participants without diabetes. Continuous measures are standardized within all three datasets.

Descriptive statistics and correlations are calculated for the sample. A correlation table is saved to a word document for reference.

```r
dataset %>%
  dplyr::select(purpose, diabetes, outcomes, covariates) %>%
  apa.cor.table(filename = paste0(here("Study 1/tables and figures/tables/"),i,"_descriptives.doc"))

descriptives <- dataset %>%
  dplyr::select(purpose, diabetes, outcomes, "age.raw", covariates) %>%
  describe(fast=T)

#diabetes only descriptives
diab.desc <- dataset %>%
  filter(diabetes == 1) %>%
  dplyr::select(purpose, diabetes, outcomes, "age.raw", covariates) %>%
  describe(fast=T)

#diabetes only alpha
if(purpose.items > 1){

  purpose.alpha.diab = dataset %>%
    filter(diabetes == 1) %>%
    select_if(grepl("purpose", names(.))) %>%
    mutate(across(everything(), as.numeric)) %>%
    psych::alpha(check.keys = TRUE)

} else (purpose.alpha.diab = NULL)

# diabetes only N
totalStudyN.diab = dataset %>% filter(diabetes == 1) %>% nrow()

if("smoker" %in% outcomes) {

  smoker.tab <- dataset %>%
    dplyr::select(diabetes, smoker) %>%
    table()

  } else {smoker.tab = NULL}

if("heart" %in% outcomes){

  heart.tab <- dataset %>%
    dplyr::select(diabetes, heart) %>%
    table()

  } else {heart.tab = NULL}
```

We calculate the correlation between purpose and self-rated health. We do this separately for the participants with and without diabetes.

```
if("srh" %in% outcomes){

  cor.diabetes <- dataset.diab %>%
    dplyr::select(purpose, srh) %>%
    cor(use="pairwise")

  cor.nodiabetes <- dataset.nod %>%
    dplyr::select(purpose, srh) %>%
    cor(use="pairwise")
} else {
  cor.diabetes = NULL
  cor.nodiabetc = NULL
}
```

We calculate estimate the difference in purpose scores between smokers and nonsmokers; we do this separately for participants with and without diabetes.

```
if("smoker" %in% outcomes){

  smoker.means.sd <- dataset %>%
    filter(!is.na(smoker)) %>%
    group_by(diabetes, smoker) %>%
    summarize(m.purpose = mean(purpose, na.rm = T),
              sd.purpose = sd(purpose, na.rm=T),
              n=n())

  d.smoker.diabetes <- dataset.diab %>%
    effsize::cohen.d(purpose ~ smoker, data =.)

  d.smoker.nodiabetes <- dataset.nod %>%
    effsize::cohen.d(purpose ~ smoker, data =.)
} else {
  smoker.means.sd = NULL
  d.smoker.diabetes = NULL
  d.smoker.nodiabetes = NULL
}
```

We calculate estimate the difference in purpose scores between participants with heart disease and participants without heart disease; we do this separately for participants with and without diabetes.

```
if("heart" %in% outcomes){

  heart.means.sd <- dataset %>%
    filter(!is.na(heart)) %>%
    group_by(diabetes, heart) %>%
    summarize(m.purpose = mean(purpose, na.rm = T),
              sd.purpose = sd(purpose, na.rm=T),
              n=n())

  d.heart.diabetes <- dataset.diab %>%
    effsize::cohen.d(purpose ~ heart, data =.)
```

```
  d.heart.nodiabetes <- dataset.nod %>%
    effsize::cohen.d(purpose ~ heart, data =.)
} else {
  heart.means.sd = NULL
  d.heart.diabetes = NULL
  d.heart.nodiabetes = NULL
}
```

Regression formulas are created, based on which covariates are available in that sample.

```
all.formula <- paste0("~ purpose*diabetes + ", paste(covariates, collapse = " + "))

one.formula <- paste0("~ purpose + ", paste(covariates, collapse = " + "))
```

We analyze the interaction of purpose and diabetes on each of the outcomes available, controlling for the covariates available. Continuous measures are standardized. Logistic regression is used for binary outcomes.

```
if("srh" %in% outcomes){
  mod.srh.all <- lm(as.formula(paste0("srh", all.formula)), data = dataset)
  } else {mod.srh.all = NULL}

if("smoker" %in% outcomes){
  mod.smoker.all <- glm(as.formula(paste0("smoker", all.formula)), data = dataset,
                        family = "binomial")
  } else {mod.smoker.all = NULL}

if("heart" %in% outcomes){
  mod.heart.all <- glm(as.formula(paste0("heart", all.formula)), data = dataset,
                       family = "binomial")
  } else {mod.heart.all = NULL}
```

We extract predicted values for these models.

```
if("srh" %in% outcomes) {plot.srh = plot_model(mod.srh.all, type="int")
  } else {plot.srh = NULL}
if("smoker" %in% outcomes) {plot.smoker = plot_model(mod.smoker.all, type="int")
  } else {plot.smoker = NULL}
if("heart" %in% outcomes) {plot.heart = plot_model(mod.heart.all, type="int")
  } else {plot.heart = NULL}
```

We analyze the relationship between purpose and each ouf the available outcomes, controlling for the covariates available. Continuous measures are standardized. Logistic regression is used for binary outcomes. We do this only for adults with diabetes.

We repeat the last set of regressions in the sample of only adults without diabetes.

Finally, we gather the analysis results into a single list and save this as a new R object. This object is saved to an external file for later use.

```
return.object = list(
  descriptives = list(
    totalN = totalStudyN,
    studyN = analysisSample,
    alpha = purpose.alpha,
    describe = descriptives,
    smoker.tab = smoker.tab,
```

```
    heart.tab = heart.tab),
  diab.desc = list(
    totalN = totalStudyN.diab,
    describe = diab.desc,
    alpha = purpose.alpha.diab
  ),
  srh = list(
    diab = cor.diabetes,
    nodiab = cor.nodiabetes),
  smoker = list(
    means = smoker.means.sd,
    diab = d.smoker.diabetes,
    nodiab = d.smoker.nodiabetes),
  heart = list(
    means = heart.means.sd,
    diab = d.heart.diabetes,
    nodiab = d.heart.nodiabetes),
  regression = list(
    interaction = list(
      srh = mod.srh.all,
      smoker = mod.smoker.all,
      heart = mod.heart.all),
    diabetes = list(
      srh = mod.srh.diab,
      smoker = mod.smoker.diab,
      heart = mod.heart.diab),
    nodiabetes = list(
      srh = mod.srh.nod,
      smoker = mod.smoker.nod,
      heart = mod.heart.nod)),
  plotdata = list(
    srh = plot.srh$data,
    smoker = plot.smoker$data,
    heart = plot.heart$data)
)

assign(x = paste(i, "out", sep="_"), return.object)
save(list = paste(i, "out", sep="_"),
     file = paste0(here("Study 1/created data/"), i, "_out.Rdata"))
```

2.2 Source script on all studies

While running the script, we also extract the number of purpose items and the mean age of the sample, which will be used in the moderation analyses when trying to explain heterogeneity between the studies.

```
study.names = c("acl", "alsa", "cloc", "elsa", "hrs", "kgss", "midja",
                "midus", "stride", "swan", "usnhms", "wls")

keys.df = data.frame(study = study.names,
                  first.key = c(1, 1, 1, 1,
                                1, 1, -1, 1,
                                1, 1, -1, 1))

study.level = data.frame(study = study.names)
```

```r
for(i in study.names){
  first.key = keys.df$first.key[keys.df$study == i]
  source(here("Study 1/scripts/study_analysis.R"))
  study.level$number_items[study.level$study == i] = purpose.items
  study.level$age[study.level$study == i] = descriptives["age", "mean"]

}
```

3 Descriptive Statistics

3.1 Mean, standard deviation, min, max, α

```
# pull the describe data.frame out of each study list
describe.df = lapply(X = study.names,
                     FUN = function(x) get(paste0(x,"_out"))$descriptives$describe) %>%
  # add to each a column called study and a column of variable names
  map2_df(., study.names, ~ mutate(.x, study = .y, var = rownames(.x))) %>%
  # dplyr::select the columns we want to include in the table
  dplyr::select(study, var, n, mean, sd, min, max) %>%
  filter(var != "age") %>%
  mutate(var = gsub("\\.raw", "", var))

# which rows belong to which study?
acl.rows = which(describe.df$study == "acl")
alsa.rows = which(describe.df$study == "alsa")
cloc.rows = which(describe.df$study == "cloc")
elsa.rows = which(describe.df$study == "elsa")
hrs.rows = which(describe.df$study == "hrs")
kgss.rows = which(describe.df$study == "kgss")
midja.rows = which(describe.df$study == "midja")
midus.rows = which(describe.df$study == "midus")
stride.rows = which(describe.df$study == "stride")
swan.rows = which(describe.df$study == "swan")
usnhms.rows = which(describe.df$study == "usnhms")
wls.rows = which(describe.df$study == "wls")

# build table
describe.df %>%
  dplyr::select(-study) %>%
  kable(.,  caption = "Descriptives Table",
        booktabs = T, escape = F, digits = 2, format = "latex", longtable = T,
        col.names = c("Variable", "N Valid", "Mean", "SD", "Min", "Max")) %>%
  kable_styling(latex_options = c("repeat_header", "hold_position")) %>%
  column_spec(1, width = "5cm") %>%
  group_rows("ACL", min(acl.rows), max(acl.rows)) %>%
  group_rows("ALSA", min(alsa.rows), max(alsa.rows)) %>%
  group_rows("CLOC", min(cloc.rows), max(cloc.rows)) %>%
  group_rows("ELSA", min(elsa.rows), max(elsa.rows)) %>%
  group_rows("HRS", min(hrs.rows), max(hrs.rows)) %>%
  group_rows("KGSS", min(kgss.rows), max(kgss.rows)) %>%
  group_rows("MIDJA", min(midja.rows), max(midja.rows)) %>%
  group_rows("MIDUS", min(midus.rows), max(midus.rows)) %>%
  group_rows("STRIDE", min(stride.rows), max(stride.rows)) %>%
  group_rows("SWAN", min(swan.rows), max(swan.rows)) %>%
  group_rows("USNHMS", min(usnhms.rows), max(usnhms.rows)) %>%
  group_rows("WLS", min(wls.rows), max(wls.rows))
```

Table 1: Descriptives Table

	Variable	N Valid	Mean	SD	Min	Max
**ACL**						
purpose...1	purpose	1648	4.33	1.01	1.00	5
diabetes...2	diabetes	1648	0.14	0.35	0.00	1
srh...3	srh	1648	3.44	1.04	1.00	5
smoker...4	smoker	1648	0.15	0.36	0.00	1
heart...5	heart	1647	0.07	0.25	0.00	1
age.raw...6	age	1648	61.52	14.32	40.00	98
gender...7	gender	1648	0.63	0.48	0.00	1
race...8	race	1648	0.27	0.44	0.00	1
edu...9	edu	1648	12.74	2.31	8.00	16
**ALSA**						
purpose...10	purpose	1524	3.39	0.94	1.00	5
diabetes...11	diabetes	1524	0.10	0.29	0.00	1
srh...12	srh	1523	3.06	1.05	1.00	5
smoker...13	smoker	1523	0.06	0.24	0.00	1
age.raw...14	age	1524	79.29	6.34	67.00	105
gender...15	gender	1524	0.50	0.50	0.00	1
edu...16	edu	1524	9.27	2.76	0.00	20
**CLOC**						
purpose...17	purpose	202	3.86	0.72	2.07	5
diabetes...18	diabetes	202	0.13	0.34	0.00	1
srh...19	srh	202	2.98	0.99	1.00	5
smoker...20	smoker	202	0.11	0.32	0.00	1
heart...21	heart	202	0.19	0.39	0.00	1
age.raw...22	age	202	75.44	6.14	57.00	92
gender...23	gender	202	0.91	0.29	0.00	1
race...24	race	202	0.15	0.36	0.00	1
edu...25	edu	202	12.04	2.40	4.00	17
**ELSA**						
purpose...26	purpose	373	3.86	0.63	1.95	5
diabetes...27	diabetes	373	0.07	0.26	0.00	1
srh...28	srh	373	3.24	1.07	1.00	5
smoker...29	smoker	373	0.13	0.34	0.00	1
heart...30	heart	373	0.16	0.37	0.00	1
age.raw...31	age	373	66.18	9.28	52.00	86
gender...32	gender	373	0.54	0.50	0.00	1
race...33	race	373	0.01	0.09	0.00	1
edu...34	edu	373	2.03	1.09	1.00	4
**HRS**						
purpose...35	purpose	20929	3.87	0.77	1.00	5
diabetes...36	diabetes	20929	0.20	0.40	0.00	1
heart...37	heart	20910	0.21	0.41	0.00	1
smoker...38	smoker	20615	0.16	0.36	0.00	1
srh...39	srh	20914	3.15	1.10	1.00	5
age.raw...40	age	20929	65.93	11.07	19.00	105
gender...41	gender	20929	0.58	0.49	0.00	1
race...42	race	20929	0.24	0.43	0.00	1

Table 1: Descriptives Table *(continued)*

	Variable	N Valid	Mean	SD	Min	Max
edu...43	edu	20929	12.65	3.15	0.00	17
**KGSS**						
purpose...44	purpose	1587	4.50	0.96	1.00	5
diabetes...45	diabetes	1587	0.05	0.21	0.00	1
srh...46	srh	1586	3.48	1.12	1.00	5
heart...47	heart	1587	0.04	0.20	0.00	1
age.raw...48	age	1587	43.47	15.21	18.00	94
gender...49	gender	1587	0.52	0.50	0.00	1
edu...50	edu	1587	4.61	1.49	1.00	8
**MIDJA**						
purpose...51	purpose	1001	3.22	0.53	1.00	5
diabetes...52	diabetes	1001	0.07	0.25	0.00	1
srh...53	srh	1001	3.49	0.79	1.00	5
smoker...54	smoker	859	0.31	0.46	0.00	1
heart...55	heart	950	0.06	0.23	0.00	1
age.raw...56	age	1001	54.17	14.01	30.00	79
gender...57	gender	1001	0.51	0.50	0.00	1
edu...58	edu	1001	4.48	2.07	1.00	8
**MIDUS**						
purpose...59	purpose	6116	4.01	0.80	1.00	5
diabetes...60	diabetes	6116	0.05	0.22	0.00	1
srh...61	srh	6109	3.55	0.98	1.00	5
smoker...62	smoker	3130	0.42	0.49	0.00	1
heart...63	heart	6109	0.06	0.24	0.00	1
age.raw...64	age	6116	46.87	12.93	20.00	75
gender...65	gender	6116	0.53	0.50	0.00	1
race...66	race	6116	0.09	0.29	0.00	1
edu...67	edu	6116	6.87	2.48	1.00	12
**STRIDE**						
purpose...68	purpose	524	2.28	0.89	1.00	5
diabetes...69	diabetes	524	0.03	0.18	0.00	1
srh...70	srh	524	2.35	0.66	1.00	5
age.raw...71	age	524	32.06	9.27	18.00	53
gender...72	gender	524	0.50	0.50	0.00	1
race...73	race	524	0.50	0.50	0.00	1
edu...74	edu	524	13.40	4.10	5.00	16
**SWAN**						
purpose...75	purpose	15442	4.67	0.67	1.00	5
diabetes...76	diabetes	15442	0.07	0.25	0.00	1
srh...77	srh	15426	3.48	1.07	1.00	5
smoker...78	smoker	15358	0.23	0.42	0.00	1
heart...79	heart	15433	0.03	0.17	0.00	1
age.raw...80	age	15442	47.09	4.34	40.00	55
edu...81	edu	15442	3.02	1.21	1.00	5
race...82	race	15442	0.50	0.50	0.00	1
**USNHMS**						
purpose...83	purpose	3801	4.09	0.71	1.00	5

Table 1: Descriptives Table *(continued)*

	Variable	N Valid	Mean	SD	Min	Max
diabetes...84	diabetes	3801	0.19	0.39	0.00	1
srh...85	srh	3798	3.30	1.09	1.00	5
smoker...86	smoker	2010	0.36	0.48	0.00	1
heart...87	heart	3790	0.13	0.33	0.00	1
age.raw...88	age	3801	60.20	14.03	35.00	89
gender...89	gender	3801	0.57	0.49	0.00	1
race...90	race	3801	0.33	0.47	0.00	1
edu...91	edu	3801	13.60	2.77	0.00	18
**WLS**						
purpose...92	purpose	10236	4.05	0.66	1.00	5
diabetes...93	diabetes	10236	0.04	0.20	0.00	1
heart...94	heart	10229	0.07	0.25	0.00	1
srh...95	srh	10205	4.14	0.67	1.00	5
smoker...96	smoker	10075	0.17	0.38	0.00	1
age.raw...97	age	10236	53.31	4.27	29.00	79
edu...98	edu	10236	13.71	2.38	0.00	21
gender...99	gender	10236	0.53	0.50	0.00	1

Participants with diabetes only

```
# pull the describe data.frame out of each study list
describe.df = lapply(X = study.names,
                     FUN = function(x) get(paste0(x,"_out"))$diab.desc$describe) %>%
  # add to each a column called study and a column of variable names
  map2_df(., study.names, ~ mutate(.x, study = .y, var = rownames(.x))) %>%
  # dplyr::select the columns we want to include in the table
  dplyr::select(study, var, n, mean, sd, min, max) %>%
  filter(var != "age") %>%
  mutate(var = gsub("\\.raw", "", var))

# which rows belong to which study?
acl.rows = which(describe.df$study == "acl")
alsa.rows = which(describe.df$study == "alsa")
cloc.rows = which(describe.df$study == "cloc")
elsa.rows = which(describe.df$study == "elsa")
hrs.rows = which(describe.df$study == "hrs")
kgss.rows = which(describe.df$study == "kgss")
midja.rows = which(describe.df$study == "midja")
midus.rows = which(describe.df$study == "midus")
stride.rows = which(describe.df$study == "stride")
swan.rows = which(describe.df$study == "swan")
usnhms.rows = which(describe.df$study == "usnhms")
wls.rows = which(describe.df$study == "wls")

# build table
describe.df %>%
  dplyr::select(-study) %>%
  kable(.,  caption = "Descriptives Table",
```

```
      booktabs = T, escape = F, digits = 2, format = "latex", longtable = T,
      col.names = c("Variable", "N Valid", "Mean", "SD", "Min", "Max")) %>%
  kable_styling(latex_options = c("repeat_header", "hold_position")) %>%
  column_spec(1, width = "5cm") %>%
  group_rows("ACL", min(acl.rows), max(acl.rows)) %>%
  group_rows("ALSA", min(alsa.rows), max(alsa.rows)) %>%
  group_rows("CLOC", min(cloc.rows), max(cloc.rows)) %>%
  group_rows("ELSA", min(elsa.rows), max(elsa.rows)) %>%
  group_rows("HRS", min(hrs.rows), max(hrs.rows)) %>%
  group_rows("KGSS", min(kgss.rows), max(kgss.rows)) %>%
  group_rows("MIDJA", min(midja.rows), max(midja.rows)) %>%
  group_rows("MIDUS", min(midus.rows), max(midus.rows)) %>%
  group_rows("STRIDE", min(stride.rows), max(stride.rows)) %>%
  group_rows("SWAN", min(swan.rows), max(swan.rows)) %>%
  group_rows("USNHMS", min(usnhms.rows), max(usnhms.rows)) %>%
  group_rows("WLS", min(wls.rows), max(wls.rows))
```

Table 2: Descriptives Table

	Variable	N Valid	Mean	SD	Min	Max
**ACL**						
purpose...1	purpose	228	4.22	1.15	1.00	5.00
diabetes...2	diabetes	228	1.00	0.00	1.00	1.00
srh...3	srh	228	2.80	1.05	1.00	5.00
smoker...4	smoker	228	0.11	0.31	0.00	1.00
heart...5	heart	227	0.11	0.32	0.00	1.00
age.raw...6	age	228	64.55	12.27	40.00	91.00
gender...7	gender	228	0.65	0.48	0.00	1.00
race...8	race	228	0.36	0.48	0.00	1.00
edu...9	edu	228	12.22	2.40	8.00	16.00
**ALSA**						
purpose...10	purpose	146	3.33	0.90	1.00	5.00
diabetes...11	diabetes	146	1.00	0.00	1.00	1.00
srh...12	srh	146	2.75	0.97	1.00	5.00
smoker...13	smoker	146	0.06	0.24	0.00	1.00
age.raw...14	age	146	79.26	6.26	68.00	100.00
gender...15	gender	146	0.44	0.50	0.00	1.00
edu...16	edu	146	9.59	3.24	2.00	20.00
**CLOC**						
purpose...17	purpose	27	3.70	0.83	2.33	5.00
diabetes...18	diabetes	27	1.00	0.00	1.00	1.00
srh...19	srh	27	2.44	0.93	1.00	4.00
smoker...20	smoker	27	0.04	0.19	0.00	1.00
heart...21	heart	27	0.15	0.36	0.00	1.00
age.raw...22	age	27	77.33	5.90	66.00	87.00
gender...23	gender	27	0.85	0.36	0.00	1.00
race...24	race	27	0.26	0.45	0.00	1.00
edu...25	edu	27	11.78	3.24	4.00	17.00
**ELSA**						
purpose...26	purpose	27	3.75	0.59	1.95	5.00

Table 2: Descriptives Table *(continued)*

	Variable	N Valid	Mean	SD	Min	Max
diabetes...27	diabetes	27	1.00	0.00	1.00	1.00
srh...28	srh	27	2.67	1.04	1.00	5.00
smoker...29	smoker	27	0.19	0.40	0.00	1.00
heart...30	heart	27	0.26	0.45	0.00	1.00
age.raw...31	age	27	69.63	9.99	52.00	84.00
gender...32	gender	27	0.48	0.51	0.00	1.00
race...33	race	27	0.00	0.00	0.00	0.00
edu...34	edu	27	2.04	1.13	1.00	4.00
**HRS**						
purpose...35	purpose	4218	3.71	0.79	1.00	5.00
diabetes...36	diabetes	4218	1.00	0.00	1.00	1.00
heart...37	heart	4213	0.33	0.47	0.00	1.00
smoker...38	smoker	4133	0.13	0.34	0.00	1.00
srh...39	srh	4217	2.58	1.00	1.00	5.00
age.raw...40	age	4218	67.05	10.21	19.00	100.00
gender...41	gender	4218	0.54	0.50	0.00	1.00
race...42	race	4218	0.33	0.47	0.00	1.00
edu...43	edu	4218	11.92	3.43	0.00	17.00
**KGSS**						
purpose...44	purpose	72	3.68	1.41	1.00	5.00
diabetes...45	diabetes	72	1.00	0.00	1.00	1.00
srh...46	srh	72	2.46	1.16	1.00	5.00
heart...47	heart	72	0.25	0.44	0.00	1.00
age.raw...48	age	72	61.67	12.45	32.00	85.00
gender...49	gender	72	0.57	0.50	0.00	1.00
edu...50	edu	72	3.35	1.81	1.00	7.00
**MIDJA**						
purpose...51	purpose	67	3.29	0.64	1.57	5.00
diabetes...52	diabetes	67	1.00	0.00	1.00	1.00
srh...53	srh	67	3.01	0.83	1.40	5.00
smoker...54	smoker	58	0.40	0.49	0.00	1.00
heart...55	heart	63	0.27	0.45	0.00	1.00
age.raw...56	age	67	63.43	10.12	38.00	79.00
gender...57	gender	67	0.33	0.47	0.00	1.00
edu...58	edu	67	4.43	2.30	1.00	8.00
**MIDUS**						
purpose...59	purpose	308	3.74	0.87	1.22	5.00
diabetes...60	diabetes	308	1.00	0.00	1.00	1.00
srh...61	srh	306	2.71	0.95	1.00	5.00
smoker...62	smoker	179	0.27	0.45	0.00	1.00
heart...63	heart	308	0.09	0.29	0.00	1.00
age.raw...64	age	308	55.35	12.04	24.00	75.00
gender...65	gender	308	0.44	0.50	0.00	1.00
race...66	race	308	0.16	0.36	0.00	1.00
edu...67	edu	308	6.27	2.68	1.00	12.00
**STRIDE**						
purpose...68	purpose	17	2.53	0.90	1.00	4.25

Table 2: Descriptives Table *(continued)*

	Variable	N Valid	Mean	SD	Min	Max
diabetes...69	diabetes	17	1.00	0.00	1.00	1.00
srh...70	srh	17	2.90	0.86	1.93	4.60
age.raw...71	age	17	35.94	9.85	18.00	53.00
gender...72	gender	17	0.35	0.49	0.00	1.00
race...73	race	17	0.65	0.49	0.00	1.00
edu...74	edu	17	11.53	5.04	5.00	16.00
**SWAN**						
purpose...75	purpose	1014	4.70	0.68	1.00	5.00
diabetes...76	diabetes	1014	1.00	0.00	1.00	1.00
srh...77	srh	1013	2.60	0.99	1.00	5.00
smoker...78	smoker	1012	0.23	0.42	0.00	1.00
heart...79	heart	1012	0.11	0.32	0.00	1.00
age.raw...80	age	1014	48.23	4.41	40.00	55.00
edu...81	edu	1014	2.69	1.15	1.00	5.00
race...82	race	1014	0.65	0.48	0.00	1.00
**USNHMS**						
purpose...83	purpose	720	3.85	0.77	1.00	5.00
diabetes...84	diabetes	720	1.00	0.00	1.00	1.00
srh...85	srh	720	2.65	1.07	1.00	5.00
smoker...86	smoker	381	0.30	0.46	0.00	1.00
heart...87	heart	715	0.25	0.43	0.00	1.00
age.raw...88	age	720	65.00	12.35	35.00	89.00
gender...89	gender	720	0.58	0.49	0.00	1.00
race...90	race	720	0.47	0.50	0.00	1.00
edu...91	edu	720	12.79	2.88	0.00	18.00
**WLS**						
purpose...92	purpose	440	3.86	0.78	1.11	5.00
diabetes...93	diabetes	440	1.00	0.00	1.00	1.00
heart...94	heart	437	0.22	0.41	0.00	1.00
srh...95	srh	438	3.55	0.73	1.00	5.00
smoker...96	smoker	436	0.15	0.36	0.00	1.00
age.raw...97	age	440	54.51	4.84	41.00	77.00
edu...98	edu	440	13.32	2.17	8.00	20.00
gender...99	gender	440	0.47	0.50	0.00	1.00

3.2 Cross-tabs

```
#identify which studies do not have smoking or heart disease
study.outcomes = describe.df %>%
  group_by(study) %>%
  summarize(srh = ifelse("srh" %in% var, 1, 0),
            smoker = ifelse("smoker" %in% var, 1, 0),
            heart = ifelse("heart" %in% var, 1, 0))


# pull the smoker cross tabs (mean and sd of purpose, plus n for each combination of
# diabetes/no diabetes and smoker/nonsmoker)
```

```
smoker.df = lapply(X = study.outcomes$study[study.outcomes$smoker == 1],
                   FUN = function(x) get(paste0(x,"_out"))$smoker$means) %>%
  # add to each a column called study
  map2_df(., study.outcomes$study[study.outcomes$smoker == 1], ~ mutate(.x, study = .y)) %>%
  # gather into long form, with each descriptive stat given a single line
  gather(key = "key", value = "var", -study, -diabetes, -smoker) %>%
  # change smoker variable
  mutate(category = ifelse(smoker == 0, "nonsmoker", "smoker")) %>%
  # remove smoker category
  dplyr::select(-smoker)

#repeat for heart disease

heart.df = lapply(X = study.outcomes$study[study.outcomes$heart == 1],
                   FUN = function(x) get(paste0(x,"_out"))$heart$means) %>%
  map2_df(., study.outcomes$study[study.outcomes$heart == 1], ~ mutate(.x, study = .y)) %>%
  gather(key = "key", value = "var", -study, -diabetes, -heart) %>%
  mutate(category = ifelse(heart == 0, "nonheart", "heart")) %>%
  dplyr::select(-heart)

# merge together

full_join(smoker.df, heart.df) %>%
  unite(variable, category, key, sep = "__") %>%
  spread(key = "variable", value = "var") %>%
  dplyr::select(study, diabetes,
              nonsmoker__n, nonsmoker__m.purpose, nonsmoker__sd.purpose,
              smoker__n, smoker__m.purpose, smoker__sd.purpose,
              nonheart__n, nonheart__m.purpose, nonheart__sd.purpose,
              heart__n, heart__m.purpose, heart__sd.purpose) %>%
  arrange(study, diabetes) %>%
  ungroup %>%
  mutate(diabetes = ifelse(diabetes == 0, "No Diabetes", "Diabetes")) %>%
  dplyr::select(-study) %>%
  kable(.,  caption = "Frequencies and purpose descriptives \\label{crosstabs}",
        booktabs = T, escape = F, digits = 2, format = "latex", longtable = T,
        col.names = c(" ",rep(c("N", "Mean", "SD"), 4))) %>%
  add_header_above(c(" " = 1, rep(c(" " = 1, "Purpose" = 2), 4))) %>%
  add_header_above(c(" ", "Non-Smoker" = 3, "Smoker" = 3,
                   "No Heart Disease" = 3, "Heart Disease" = 3)) %>%
  column_spec(1, width = "5cm") %>%
  group_rows("ACL", 1,2) %>%
  group_rows("ALSA", 3,4) %>%
  group_rows("CLOC", 5,6) %>%
  group_rows("ELSA", 7,8) %>%
  group_rows("HRS", 9,10) %>%
  group_rows("KGSS", 11,12) %>%
  group_rows("MIDJA", 13,14) %>%
  group_rows("MIDUS", 15,16) %>%
  group_rows("SWAN", 17,18) %>%
  group_rows("USNHMS", 19,20) %>%
```

```
  group_rows("WLS", 21,22) %>%
    footnote(general = "Project STRIDE did not have either smoking status or heart disease status, and s
```

Table 3: Frequencies and purpose descriptives

	Non-Smoker			Smoker			No Heart Disease			Heart Disease		
		Purpose			Purpose			Purpose			Purpose	
	N	Mean	SD	N	Mean	SD	N	Mean	SD	N	Mean	SD
**ACL**												
No Diabetes	1194	4.36	0.97	226	4.29	1.05	1338	4.37	0.97	82	4.11	1.11
Diabetes	204	4.22	1.17	24	4.22	1.03	201	4.22	1.16	26	4.23	1.14
**ALSA**												
No Diabetes	1293	3.42	0.94	84	3.06	0.99						
Diabetes	137	3.34	0.90	9	3.19	0.94						
**CLOC**												
No Diabetes	153	3.92	0.68	22	3.60	0.86	141	3.89	0.72	34	3.85	0.68
Diabetes	26	3.66	0.82	1	4.73		23	3.78	0.76	4	3.27	1.16
**ELSA**												
No Diabetes	302	3.92	0.62	44	3.57	0.69	292	3.89	0.62	54	3.76	0.70
Diabetes	22	3.72	0.65	5	3.88	0.26	20	3.89	0.52	7	3.37	0.67
**HRS**												
No Diabetes	13804	3.94	0.74	2678	3.75	0.81	13587	3.94	0.75	3110	3.77	0.79
Diabetes	3586	3.73	0.78	547	3.63	0.82	2838	3.77	0.78	1375	3.60	0.79
**KGSS**												
No Diabetes							1470	4.56	0.89	45	3.80	1.31
Diabetes							54	3.76	1.33	18	3.44	1.65
**MIDJA**												
No Diabetes	560	3.24	0.51	241	3.12	0.53	851	3.22	0.52	36	3.24	0.46
Diabetes	35	3.37	0.65	23	3.13	0.58	46	3.30	0.64	17	3.43	0.53
**MIDUS**												
No Diabetes	1682	4.00	0.81	1269	3.87	0.82	5442	4.04	0.78	359	3.88	0.89
Diabetes	130	3.76	0.86	49	3.65	0.82	279	3.76	0.86	29	3.54	0.97
**SWAN**												
No Diabetes	11069	4.67	0.66	3277	4.67	0.67	14087	4.67	0.67	334	4.72	0.61
Diabetes	784	4.71	0.68	228	4.66	0.67	897	4.70	0.68	115	4.68	0.63
**USNHMS**												
No Diabetes	1023	4.14	0.65	606	4.04	0.76	2775	4.17	0.67	300	3.93	0.71
Diabetes	265	3.94	0.70	116	3.65	0.87	535	3.91	0.77	180	3.70	0.75
**WLS**												
No Diabetes	7948	4.07	0.64	1691	4.02	0.69	9222	4.07	0.65	570	3.95	0.69
Diabetes	370	3.87	0.75	66	3.81	0.92	342	3.95	0.75	95	3.57	0.83

Note:
Project STRIDE did not have either smoking status or heart disease status, and so is not included in this table.

Note in Table 3 that the CLOC study has only one participant who both has diabetes and is a smoker. This is not enough information for our analyses, and so we will not use the CLOC in our meta analysis.

4 Bivariate effect sizes

4.1 Correlation between purpose and self-rated health

```
cor.srh = data.frame(study = study.names)
cor.srh$n_all = unlist(lapply(X = study.names,
                              FUN = function(x) get(paste0(x,"_out"))$descriptives$studyN))
cor.srh$n_diabetes = unlist(lapply(X = study.names,
                              FUN = function(x) get(paste0(x,"_out"))$descriptives$studyN*get(paste0(x,"_
cor.srh$diabetes = unlist(lapply(X = study.names,
                             FUN = function(x) get(paste0(x,"_out"))$srh$diab["purpose","srh"]))
cor.srh$n_nodiabetes = cor.srh$n_all - cor.srh$n_diabetes
cor.srh$nodiabetes = unlist(lapply(X = study.names,
                             FUN = function(x) get(paste0(x,"_out"))$srh$nodiab["purpose","srh"]))

cor.srh = cor.srh %>%
  dplyr::select(-n_all) %>%
  mutate(t_diabetes = diabetes/sqrt((1-(diabetes^2))/(n_diabetes-2)),
         t_nodiabetes = nodiabetes/sqrt((1-(nodiabetes^2))/(n_nodiabetes-2)),
         p_diabetes = pt(q = abs(t_diabetes), df = n_diabetes-2, lower.tail=F)*2,
         p_nodiabetes = pt(q = abs(t_nodiabetes), df = n_nodiabetes-2, lower.tail=F)*2) %>%
  mutate(study = toupper(study)) %>%
  dplyr::select(study, n_diabetes, diabetes, p_diabetes,
              n_nodiabetes, nodiabetes, p_nodiabetes)

kable(cor.srh,  caption = "Correlations between purpose and self-rated health",
        booktabs = T, escape = F, digits = 3, format = "latex", longtable = T,
        col.names = c("Study", "N", "$r$", "$p$", "N", "$r$", "$p$")) %>%
    add_header_above(c(" ", "Adults with diabetes" = 3, "Adults without diabetes" = 3)) %>%
  kable_styling(latex_options = c("repeat_header", "hold_position")) %>%
  column_spec(1, width = "4cm")
```

Table 4: Correlations between purpose and self-rated health

	Adults with diabetes			Adults without diabetes		
Study	N	r	p	N	r	p
ACL	228	0.154	0.020	1420	0.181	0.000
ALSA	146	0.209	0.011	1378	0.272	0.000
CLOC	27	-0.027	0.892	175	0.303	0.000
ELSA	27	0.392	0.043	346	0.331	0.000
HRS	4218	0.268	0.000	16711	0.287	0.000
KGSS	72	0.348	0.003	1515	0.275	0.000
MIDJA	67	0.348	0.004	934	0.214	0.000
MIDUS	308	0.204	0.000	5808	0.225	0.000
STRIDE	17	0.318	0.213	507	0.127	0.004
SWAN	1014	0.095	0.002	14428	0.037	0.000
USNHMS	720	0.274	0.000	3081	0.292	0.000
WLS	440	0.303	0.000	9796	0.273	0.000

4.2 Standardized difference in purpose between smokers and non-smokers

```
smoker.names = study.outcomes$study[study.outcomes$smoker == 1]
smoker.names = smoker.names[!(smoker.names == "cloc")]

d.smoker = data.frame(study = smoker.names)
d.smoker$diabetes_d = unlist(lapply(X = smoker.names,
                            FUN = function(x) get(paste0(x,"_out"))$smoker$diab$estimate))
d.smoker$diabetes_ub = unlist(lapply(X = smoker.names,
                            FUN = function(x) get(paste0(x,"_out"))$smoker$diab$conf.int[2]))
d.smoker$nondiabetes_d = unlist(lapply(X = smoker.names,
                            FUN = function(x) get(paste0(x,"_out"))$smoker$nond$estimate))
d.smoker$nondiabetes_ub = unlist(lapply(X = smoker.names,
                            FUN = function(x) get(paste0(x,"_out"))$smoker$nond$conf.int[2]))

d.smoker = d.smoker %>%
  gather(key = "diabetes", value = "value", -study) %>%
  separate(diabetes, into =c("diabetes","statistic")) %>%
  spread(key = "statistic", value = "value") %>%
  mutate(diabetes = ifelse(grepl("non", diabetes), 0, 1)) %>%
  full_join(smoker.df) %>%
  unite(key, key, category) %>%
  spread(key = "key", value = "var") %>%
  mutate(t.sig = qt(p = .025, df = (n_nonsmoker + n_smoker -2), lower.tail = F),
         se = abs(ub-d)/t.sig,
         p = pt(abs(d/se), df = (n_nonsmoker + n_smoker -2), lower.tail = F)) %>%
  arrange(study, diabetes) %>%
  mutate(diabetes = ifelse(diabetes == 0, "No Diabetes", "Diabetes"))

d.smoker %>%
  dplyr::select(diabetes, n_smoker, m.purpose_smoker, sd.purpose_smoker,
              n_nonsmoker, m.purpose_nonsmoker, sd.purpose_nonsmoker, d, p) %>%
kable(., caption = "Differences in purpose between smokers and non-smokers \\label{dsmoker}",
        booktabs = T, escape = F, digits = 3, format = "latex", longtable = T,
        col.names = c(" ", rep(c("N", "Mean", "SD"), 2), "Cohen's D", "p value")) %>%
  add_header_above(c(" ", "Smokers" = 3, "Non-Smokers" = 3, " ", " ")) %>%
  column_spec(1, width = "5cm") %>%
  group_rows("ACL", 1,2) %>%
  group_rows("ALSA", 3,4) %>%
  group_rows("ELSA", 5,6) %>%
  group_rows("HRS", 7,8) %>%
  group_rows("KGSS", 9,10) %>%
  group_rows("MIDJA", 11,12) %>%
  group_rows("MIDUS", 13,14) %>%
  group_rows("SWAN", 15,16) %>%
  group_rows("USNHMS", 17,18) %>%
  group_rows("WLS", 19,20)
```

Table 5: Differences in purpose between smokers and non-smokers

	Smokers			Non-Smokers				

	N	Mean	SD	N	Mean	SD	Cohen's D	p value
**ACL**								
No Diabetes	226	4.292	1.045	1194	4.362	0.970		
Diabetes	24	4.222	1.034	204	4.222	1.167	0.000	0.500
**ALSA**								
No Diabetes	84	3.063	0.992	1293	3.419	0.939		
Diabetes	9	3.193	0.941	137	3.338	0.900	0.161	0.320
**ELSA**								
No Diabetes	22	3.597	0.861	153	3.920	0.676		
Diabetes	1	4.733		26	3.662	0.816		
**HRS**								
No Diabetes	44	3.574	0.694	302	3.915	0.619		
Diabetes	5	3.876	0.264	22	3.723	0.648	-0.254	0.307
**KGSS**								
No Diabetes	2678	3.754	0.808	13804	3.938	0.743		
Diabetes	547	3.630	0.820	3586	3.727	0.781	0.124	0.004
**MIDJA**								
No Diabetes	241	3.117	0.531	560	3.243	0.511		
Diabetes	23	3.133	0.582	35	3.367	0.650	0.375	0.086
**MIDUS**								
No Diabetes	1269	3.868	0.817	1682	3.999	0.812		
Diabetes	49	3.649	0.821	130	3.756	0.861	0.126	0.227
**SWAN**								
No Diabetes	3277	4.666	0.669	11069	4.669	0.663		
Diabetes	228	4.664	0.667	784	4.710	0.681	0.068	0.184
**USNHMS**								
No Diabetes	606	4.038	0.755	1023	4.143	0.653		
Diabetes	116	3.645	0.870	265	3.943	0.695	0.395	0.000
**WLS**								
No Diabetes	1691	4.016	0.691	7948	4.074	0.644		
Diabetes	66	3.808	0.922	370	3.872	0.752	0.082	0.270

4.3 Standardized difference in purpose between individuals with heart disease and individuals without

```
heart.names = study.outcomes$study[study.outcomes$heart == 1]
heart.names = heart.names[!(heart.names == "cloc")]

d.heart = data.frame(study = heart.names)
d.heart$diabetes_d = unlist(lapply(X = heart.names,
                         FUN = function(x) get(paste0(x,"_out"))$heart$diab$estimate))
d.heart$diabetes_ub = unlist(lapply(X = heart.names,
                         FUN = function(x) get(paste0(x,"_out"))$heart$diab$conf.int[2]))
d.heart$nondiabetes_d = unlist(lapply(X = heart.names,
                         FUN = function(x) get(paste0(x,"_out"))$heart$nond$estimate))
d.heart$nondiabetes_ub = unlist(lapply(X = heart.names,
                         FUN = function(x) get(paste0(x,"_out"))$heart$nond$conf.int[2]))
```

```
d.heart = d.heart %>%
  gather(key = "diabetes", value = "value", -study) %>%
  separate(diabetes, into =c("diabetes","statistic")) %>%
  spread(key = "statistic", value = "value") %>%
  mutate(diabetes = ifelse(grepl("non", diabetes), 0, 1)) %>%
  full_join(heart.df) %>%
  unite(key, key, category) %>%
  spread(key = "key", value = "var") %>%
  mutate(t.sig = qt(p = .025, df = (n_nonheart + n_heart -2), lower.tail = F),
         se = abs(ub-d)/t.sig,
         p = pt(abs(d/se), df = (n_nonheart + n_heart -2), lower.tail = F)) %>%
  arrange(study, diabetes) %>%
  mutate(diabetes = ifelse(diabetes == 0, "No Diabetes", "Diabetes"))

d.heart %>%
  dplyr::select(diabetes, n_heart, m.purpose_heart, sd.purpose_heart,
                n_nonheart, m.purpose_nonheart, sd.purpose_nonheart, d, p) %>%
kable(.,
      caption = "Differences in purpose between individuals with heart disease and individuals without \\
        booktabs = T, escape = F, digits = 3, format = "latex", longtable = T,
        col.names = c(" ", rep(c("N", "Mean", "SD"), 2), "Cohen's D", "p value")) %>%
  add_header_above(c(" ", "Heart Disease" = 3, "No Heart Disease" = 3, " ", " ")) %>%
  column_spec(1, width = "5cm") %>%
  group_rows("ACL", 1,2) %>%
  group_rows("ALSA", 3,4) %>%
  group_rows("ELSA", 5,6) %>%
  group_rows("HRS", 7,8) %>%
  group_rows("KGSS", 9,10) %>%
  group_rows("MIDJA", 11,12) %>%
  group_rows("MIDUS", 13,14) %>%
  group_rows("SWAN", 15,16) %>%
  group_rows("USNHMS", 17,18) %>%
  group_rows("WLS", 19,20)
```

Table 6: Differences in purpose between individuals with heart disease and individuals without

	Heart Disease			No Heart Disease				
	N	Mean	SD	N	Mean	SD	Cohen's D	p value
**ACL**								
No Diabetes	82	4.106	1.110	1338	4.366	0.973		
Diabetes	26	4.231	1.142	201	4.217	1.157	-0.012	0.478
**ALSA**								
No Diabetes	34	3.847	0.675	141	3.887	0.717		
Diabetes	4	3.267	1.162	23	3.777	0.763		
**ELSA**								
No Diabetes	54	3.764	0.700	292	3.892	0.625		
Diabetes	7	3.367	0.672	20	3.886	0.517	0.929	0.026
**HRS**								
No Diabetes	3110	3.774	0.790	13587	3.941	0.746		

Diabetes	1375	3.596	0.792	2838	3.770	0.777	0.222	0.000
**KGSS**								
No Diabetes	45	3.800	1.307	1470	4.557	0.891		
Diabetes	18	3.444	1.653	54	3.759	1.331	0.222	0.209
**MIDJA**								
No Diabetes	36	3.235	0.464	851	3.221	0.523		
Diabetes	17	3.431	0.529	46	3.296	0.637	-0.222	0.219
**MIDUS**								
No Diabetes	359	3.884	0.890	5442	4.036	0.782		
Diabetes	29	3.540	0.974	279	3.755	0.859	0.247	0.103
**SWAN**								
No Diabetes	334	4.719	0.606	14087	4.667	0.666		
Diabetes	115	4.681	0.633	897	4.701	0.684	0.029	0.385
**USNHMS**								
No Diabetes	300	3.932	0.707	2775	4.174	0.675		
Diabetes	180	3.701	0.747	535	3.906	0.766	0.270	0.001
**WLS**								
No Diabetes	570	3.952	0.690	9222	4.070	0.650		
Diabetes	95	3.572	0.831	342	3.949	0.746	0.494	0.000

5 Meta-analysis: Interaction

5.1 Self-rated health

Here we extract the data from the lists and format a data frame for analysis. We need the estimates and standard errors of the purpose coefficients for the diabetes and no-diabetes models, and we need the estimates and standard errors of the interaction terms for the combined models. We also need the sample sizes for each model.

```
# gather regression estimates into a data frame
# each row is one study
# each column is one regression model
srh.data = data.frame(study = study.names)
srh.data$diab = lapply(X = study.names,
                      FUN = function(x) get(paste0(x,"_out"))$regression$diabetes$srh)
srh.data$nodiab = lapply(X = study.names,
                      FUN = function(x) get(paste0(x,"_out"))$regression$nodiabetes$srh)
srh.data$interaction = lapply(X = study.names,
                      FUN = function(x) get(paste0(x,"_out"))$regression$interaction$srh)


srh.data = srh.data %>%
  # gather so all regression models are in one column
  gather(key = "model", value = "value", -study) %>%
  # tidy
  mutate(tidy = map(value, broom::tidy)) %>%
  # unnest the tidy (the coefficients become the data frame)
  unnest(tidy, .drop = T) %>%
  #we only need the estimate and standard error for these models
  dplyr::select(study, model, term, estimate, std.error) %>%
  # and we don't care about the intercept or covariates
  filter(term %in% c("purpose", "purpose:diabetes")) %>%
```

```r
  # and for the interaction models, we only care about the interaction term
  filter(!(term == "purpose" & model == "interaction")) %>%
  dplyr::select(-term) %>%
  # pull the estimates and standard errors into a single column
  gather(key = "key", value = "value", -study, -model) %>%
  # merge these two columns
  unite(stat, model, key) %>%
  # spread back out. now each of our estimates and se's have their own columns
  spread(key = "stat", value = "value")

## Warning:  The `.drop` argument of `unnest()` is deprecated as of tidyr 1.0.0.
## i All list-columns are now preserved.
## Call `lifecycle::last_lifecycle_warnings()` to see where this warning was
## generated.

# We need the sample sizes still
srh.data$n_all = unlist(lapply(X = study.names,
                     FUN = function(x) length(
                       get(paste0(x,"_out"))$regression$interaction$srh$residuals)))
srh.data$n_diabetes = unlist(lapply(X = study.names,
                     FUN = function(x) length(
                       get(paste0(x,"_out"))$regression$diabetes$srh$residuals)))
srh.data$n_nodiabetes = unlist(lapply(X = study.names,
                     FUN = function(x) length(
                       get(paste0(x,"_out"))$regression$nodiabetes$srh$residuals)))
```

Now we run the meta-analysis models and save these to output for later use.

```r
meta.srh.diabetes <- rma(yi = diab_estimate,
                         sei = diab_std.error,
                         ni = n_diabetes,
                         slab = study, method="REML", data = srh.data)

summary(meta.srh.diabetes)

##
## Random-Effects Model (k = 12; tau^2 estimator: REML)
##
##   logLik  deviance       AIC       BIC      AICc
##   5.7397  -11.4794   -7.4794   -6.6836   -5.9794
##
## tau^2 (estimated amount of total heterogeneity): 0.0046 (SE = 0.0040)
## tau (square root of estimated tau^2 value):      0.0679
## I^2 (total heterogeneity / total variability):   58.32%
## H^2 (total variability / sampling variability):  2.40
##
## Test for Heterogeneity:
## Q(df = 11) = 31.6414, p-val = 0.0009
##
## Model Results:
##
## estimate      se    zval    pval   ci.lb   ci.ub
##   0.2266  0.0301  7.5172  <.0001  0.1675  0.2856  ***
##
## ---
## Signif. codes:  0 '***' 0.001 '**' 0.01 '*' 0.05 '.' 0.1 ' ' 1
```

```r
meta.srh.nodiabetes <- rma(yi = nodiab_estimate,
                           sei = nodiab_std.error,
                           ni = n_nodiabetes,
                           slab = study, method="REML", data = srh.data)

summary(meta.srh.nodiabetes)
```

```
##
## Random-Effects Model (k = 12; tau^2 estimator: REML)
##
##   logLik  deviance       AIC       BIC      AICc
##   8.0990  -16.1980  -12.1980  -11.4022  -10.6980
##
## tau^2 (estimated amount of total heterogeneity): 0.0108 (SE = 0.0052)
## tau (square root of estimated tau^2 value):      0.1040
## I^2 (total heterogeneity / total variability):   96.71%
## H^2 (total variability / sampling variability):  30.42
##
## Test for Heterogeneity:
## Q(df = 11) = 308.6397, p-val < .0001
##
## Model Results:
##
## estimate      se    zval    pval   ci.lb   ci.ub
##   0.2425  0.0321  7.5475  <.0001  0.1795  0.3055  ***
##
## ---
## Signif. codes:  0 '***' 0.001 '**' 0.01 '*' 0.05 '.' 0.1 ' ' 1
```

```r
meta.srh.interaction <- rma(yi = interaction_estimate,
                            sei = interaction_std.error,
                            ni = n_all,
                            slab = study, method="REML", data = srh.data)

summary(meta.srh.interaction)
```

```
##
## Random-Effects Model (k = 12; tau^2 estimator: REML)
##
##   logLik  deviance       AIC       BIC      AICc
##  10.5985  -21.1970  -17.1970  -16.4012  -15.6970
##
## tau^2 (estimated amount of total heterogeneity): 0.0007 (SE = 0.0015)
## tau (square root of estimated tau^2 value):      0.0257
## I^2 (total heterogeneity / total variability):   16.14%
## H^2 (total variability / sampling variability):  1.19
##
## Test for Heterogeneity:
## Q(df = 11) = 12.2744, p-val = 0.3434
##
## Model Results:
##
## estimate      se     zval    pval    ci.lb   ci.ub
##  -0.0216  0.0191  -1.1267  0.2599  -0.0591  0.0160
##
## ---
## Signif. codes:  0 '***' 0.001 '**' 0.01 '*' 0.05 '.' 0.1 ' ' 1
```

We repeat this code for the smoker models and the heart disease models.

5.2 Smoking status

Here we extract the data from the lists and format a data frame for analysis. We need the estimates and standard errors of the purpose coefficients for the diabetes and no-diabetes models, and we need the estimates and standard errors of the interaction terms for the combined models. We also need the sample sizes for each model.

As a reminder, CLOC did not have substantial covariance and therefore was excluded from these analyses (see Table 3 for values). We also removed the studies ALSA and ELSA for having too few smokers after recommendation by peer reviewers.

```
smoker.names = study.outcomes$study[study.outcomes$smoker == 1]
smoker.names = smoker.names[!(smoker.names %in% c("cloc", "alsa", "elsa"))]

# gather regression estimates into a data frame
# each row is one study
# each column is one regression model


smoker.data = data.frame(study = smoker.names)
smoker.data$diab = lapply(X = smoker.names,
                     FUN = function(x) get(paste0(x,"_out"))$regression$diabetes$smoker)
smoker.data$nodiab = lapply(X = smoker.names,
                     FUN = function(x) get(paste0(x,"_out"))$regression$nodiabetes$smoker)
smoker.data$interaction = lapply(X = smoker.names,
                     FUN = function(x) get(paste0(x,"_out"))$regression$interaction$smoker)


smoker.data = smoker.data %>%
  # gather so all regression models are in one column
  gather(key = "model", value = "value", -study) %>%
  # tidy
  mutate(tidy = map(value, broom::tidy)) %>%
  # unnest the tidy (the coefficients become the data frame)
  unnest(tidy, .drop = T) %>%
  #we only need the estimate and standard error for these models
  dplyr::select(study, model, term, estimate, std.error) %>%
  # and we don't care about the intercept or covariates
  filter(term %in% c("purpose", "purpose:diabetes")) %>%
  # and for the interaction models, we only care about the interaction term
  filter(!(term == "purpose" & model == "interaction")) %>%
  dplyr::select(-term) %>%
  # pull the estimates and standard errors into a single column
  gather(key = "key", value = "value", -study, -model) %>%
  # merge these two columns
  unite(stat, model, key) %>%
  # spread back out. now each of our estimates and se's have their own columns
  spread(key = "stat", value = "value")

## Warning: The `.drop` argument of `unnest()` is deprecated as of tidyr 1.0.0.
## i All list-columns are now preserved.
## Call `lifecycle::last_lifecycle_warnings()` to see where this warning was
## generated.

# We need the sample sizes still
```

```
smoker.data$n_all = unlist(lapply(X = smoker.names,
                     FUN = function(x) length(
                       get(paste0(x,"_out"))$regression$interaction$smoker$residuals)))
smoker.data$n_diabetes = unlist(lapply(X = smoker.names,
                     FUN = function(x) length(
                       get(paste0(x,"_out"))$regression$diabetes$smoker$residuals)))
smoker.data$n_nodiabetes = unlist(lapply(X = smoker.names,
                     FUN = function(x) length(
                       get(paste0(x,"_out"))$regression$nodiabetes$smoker$residuals)))
```

Now we run the meta-analysis models and save these to output for later use.

```
meta.smoker.diabetes <- rma(yi = diab_estimate,
                            sei = diab_std.error,
                            ni = n_diabetes,
                            slab = study, method="REML",
                            measure = "OR", data = smoker.data)

summary(meta.smoker.diabetes)

##
## Random-Effects Model (k = 7; tau^2 estimator: REML)
##
##   logLik  deviance       AIC       BIC      AICc
##   2.5368   -5.0735   -1.0735   -1.4900    2.9265
##
## tau^2 (estimated amount of total heterogeneity): 0 (SE = 0.0084)
## tau (square root of estimated tau^2 value):      0
## I^2 (total heterogeneity / total variability):   0.00%
## H^2 (total variability / sampling variability):  1.00
##
## Test for Heterogeneity:
## Q(df = 6) = 4.3093, p-val = 0.6349
##
## Model Results:
##
## estimate      se     zval    pval    ci.lb    ci.ub
##  -0.1813  0.0461  -3.9305  <.0001  -0.2716  -0.0909  ***
##
## ---
## Signif. codes:  0 '***' 0.001 '**' 0.01 '*' 0.05 '.' 0.1 ' ' 1

meta.smoker.nodiabetes <- rma(yi = nodiab_estimate,
                              sei = nodiab_std.error,
                              ni = n_nodiabetes,
                              slab = study, method="REML",
                              measure = "OR", data = smoker.data)

summary(meta.smoker.nodiabetes)

##
## Random-Effects Model (k = 7; tau^2 estimator: REML)
##
##   logLik  deviance       AIC       BIC      AICc
##   3.4063   -6.8127   -2.8127   -3.2292    1.1873
##
```

```
## tau^2 (estimated amount of total heterogeneity): 0.0160 (SE = 0.0116)
## tau (square root of estimated tau^2 value):      0.1263
## I^2 (total heterogeneity / total variability):   88.06%
## H^2 (total variability / sampling variability):  8.38
##
## Test for Heterogeneity:
## Q(df = 6) = 82.2869, p-val < .0001
##
## Model Results:
##
## estimate      se     zval    pval    ci.lb    ci.ub
##  -0.1550  0.0539  -2.8741  0.0041  -0.2607  -0.0493  **
##
## ---
## Signif. codes:  0 '***' 0.001 '**' 0.01 '*' 0.05 '.' 0.1 ' ' 1

meta.smoker.interaction <- rma(yi = interaction_estimate,
                               sei = interaction_std.error,
                               ni = n_all,
                               slab = study, method="REML",
                               control=list(stepadj=0.5),
                               measure = "OR", data = smoker.data)

summary(meta.smoker.interaction)

##
## Random-Effects Model (k = 7; tau^2 estimator: REML)
##
##   logLik  deviance       AIC       BIC      AICc
##   2.5164   -5.0328   -1.0328   -1.4492    2.9672
##
## tau^2 (estimated amount of total heterogeneity): 0.0057 (SE = 0.0147)
## tau (square root of estimated tau^2 value):      0.0756
## I^2 (total heterogeneity / total variability):   20.88%
## H^2 (total variability / sampling variability):  1.26
##
## Test for Heterogeneity:
## Q(df = 6) = 4.9094, p-val = 0.5555
##
## Model Results:
##
## estimate      se    zval    pval    ci.lb   ci.ub
##   0.0662  0.0629  1.0525  0.2926  -0.0570  0.1893
##
## ---
## Signif. codes:  0 '***' 0.001 '**' 0.01 '*' 0.05 '.' 0.1 ' ' 1
```

5.3 Heart Disease

Here we extract the data from the lists and format a data frame for analysis. We need the estimates and standard errors of the purpose coefficients for the diabetes and no-diabetes models, and we need the estimates and standard errors of the interaction terms for the combined models. We also need the sample sizes for each model.

```r
heart.names = study.outcomes$study[study.outcomes$heart == 1]

# gather regression estimates into a data frame
# each row is one study
# each column is one regression model
heart.data = data.frame(study = heart.names)
heart.data$diab = lapply(X = heart.names,
                     FUN = function(x) get(paste0(x,"_out"))$regression$diabetes$heart)
heart.data$nodiab = lapply(X = heart.names,
                     FUN = function(x) get(paste0(x,"_out"))$regression$nodiabetes$heart)
heart.data$interaction = lapply(X = heart.names,
                     FUN = function(x) get(paste0(x,"_out"))$regression$interaction$heart)


heart.data = heart.data %>%
  # gather so all regression models are in one column
  gather(key = "model", value = "value", -study) %>%
  # tidy
  mutate(tidy = map(value, broom::tidy)) %>%
  # unnest the tidy (the coefficients become the data frame)
  unnest(tidy, .drop = T) %>%
  #we only need the estimate and standard error for these models
  dplyr::select(study, model, term, estimate, std.error) %>%
  # and we don't care about the intercept or covariates
  filter(term %in% c("purpose", "purpose:diabetes")) %>%
  # and for the interaction models, we only care about the interaction term
  filter(!(term == "purpose" & model == "interaction")) %>%
  dplyr::select(-term) %>%
  # pull the estimates and standard errors into a single column
  gather(key = "key", value = "value", -study, -model) %>%
  # merge these two columns
  unite(stat, model, key) %>%
  # spread back out. now each of our estimates and se's have their own columns
  spread(key = "stat", value = "value")

## Warning:  The `.drop` argument of `unnest()` is deprecated as of tidyr 1.0.0.
## i All list-columns are now preserved.
## Call `lifecycle::last_lifecycle_warnings()` to see where this warning was
## generated.

# We need the sample sizes still
heart.data$n_all = unlist(lapply(X = heart.names,
                     FUN = function(x) length(
                       get(paste0(x,"_out"))$regression$interaction$heart$residuals)))
heart.data$n_diabetes = unlist(lapply(X = heart.names,
                     FUN = function(x) length(
                       get(paste0(x,"_out"))$regression$diabetes$heart$residuals)))
heart.data$n_nodiabetes = unlist(lapply(X = heart.names,
                     FUN = function(x) length(
                       get(paste0(x,"_out"))$regression$nodiabetes$heart$residuals)))
```

Now we run the meta-analysis models and save these to output for later use.

```r
meta.heart.diabetes <- rma(yi = diab_estimate,
                         sei = diab_std.error,
                         ni = n_diabetes,
                         slab = study, method="REML",
```

```r
                         measure = "OR", data = heart.data)

summary(meta.heart.diabetes)

##
## Random-Effects Model (k = 10; tau^2 estimator: REML)
##
##   logLik  deviance       AIC       BIC      AICc
##  -1.9519    3.9037    7.9037    8.2982    9.9037
##
## tau^2 (estimated amount of total heterogeneity): 0.0207 (SE = 0.0240)
## tau (square root of estimated tau^2 value):      0.1439
## I^2 (total heterogeneity / total variability):   45.72%
## H^2 (total variability / sampling variability):  1.84
##
## Test for Heterogeneity:
## Q(df = 9) = 12.8382, p-val = 0.1701
##
## Model Results:
##
## estimate      se     zval    pval    ci.lb    ci.ub
##  -0.2495  0.0772  -3.2308  0.0012  -0.4008  -0.0981  **
##
## ---
## Signif. codes:  0 '***' 0.001 '**' 0.01 '*' 0.05 '.' 0.1 ' ' 1

meta.heart.nodiabetes <- rma(yi = nodiab_estimate,
                            sei = nodiab_std.error,
                            ni = n_nodiabetes,
                            slab = study, method="REML",
                            measure = "OR", data = heart.data)

summary(meta.heart.nodiabetes)

##
## Random-Effects Model (k = 10; tau^2 estimator: REML)
##
##   logLik  deviance       AIC       BIC      AICc
##   4.3474   -8.6948   -4.6948   -4.3003   -2.6948
##
## tau^2 (estimated amount of total heterogeneity): 0.0053 (SE = 0.0063)
## tau (square root of estimated tau^2 value):      0.0731
## I^2 (total heterogeneity / total variability):   43.98%
## H^2 (total variability / sampling variability):  1.78
##
## Test for Heterogeneity:
## Q(df = 9) = 13.4794, p-val = 0.1421
##
## Model Results:
##
## estimate      se     zval    pval    ci.lb    ci.ub
##  -0.1356  0.0392  -3.4561  0.0005  -0.2125  -0.0587  ***
##
## ---
## Signif. codes:  0 '***' 0.001 '**' 0.01 '*' 0.05 '.' 0.1 ' ' 1

meta.heart.interaction <- rma(yi = interaction_estimate,
```

```
                            sei = interaction_std.error,
                            ni = n_all,
                            slab = study, method="REML",
                            measure = "OR", data = heart.data)

summary(meta.heart.interaction)

##
## Random-Effects Model (k = 10; tau^2 estimator: REML)
##
##   logLik  deviance       AIC       BIC      AICc
##  -0.9501    1.9002    5.9002    6.2947    7.9002
##
## tau^2 (estimated amount of total heterogeneity): 0 (SE = 0.0090)
## tau (square root of estimated tau^2 value):      0
## I^2 (total heterogeneity / total variability):   0.00%
## H^2 (total variability / sampling variability):  1.00
##
## Test for Heterogeneity:
## Q(df = 9) = 8.3319, p-val = 0.5011
##
## Model Results:
##
## estimate      se     zval    pval    ci.lb   ci.ub
##  -0.0697  0.0423  -1.6476  0.0994  -0.1527  0.0132  .
##
## ---
## Signif. codes:  0 '***' 0.001 '**' 0.01 '*' 0.05 '.' 0.1 ' ' 1
```

5.4 Summarize meta analyses

We combine all values into a single data frame for summary purposes.

```
srh.data$outcome = "srh"
smoker.data$outcome = "smoker"
heart.data$outcome = "heart"

all.meta <- full_join(srh.data, smoker.data) %>%
  full_join(heart.data)
```

We calculate the odds ratios for the smoker and heart disease models.

```
all.meta = all.meta %>%
  mutate(diabetes.OR = exp(diab_estimate),
         nondiabetes.OR = exp(nodiab_estimate),
         interaction.OR = exp(interaction_estimate))

all.meta$diabetes.OR = printnum(all.meta$diabetes.OR)
all.meta$nondiabetes.OR = printnum(all.meta$nondiabetes.OR)
all.meta$interaction.OR = printnum(all.meta$interaction.OR)

all.meta$diabetes.OR[all.meta$outcome == "srh"] = NA
all.meta$nondiabetes.OR[all.meta$outcome == "srh"] = NA
all.meta$interaction.OR[all.meta$outcome == "srh"] = NA
```

We format the estimates and confidence intervals for the main effects of purpose in each study.

```r
all.meta = all.meta %>%
  mutate(diab_ci_lb = diab_estimate - 1.96*diab_std.error,
         diab_ci_ub = diab_estimate + 1.96*diab_std.error,
         nodiab_ci_lb = nodiab_estimate - 1.96*nodiab_std.error,
         nodiab_ci_ub = nodiab_estimate + 1.96*nodiab_std.error)
```

```r
all.meta$diab_estimate = printnum(all.meta$diab_estimate)
all.meta$diab_ci_lb = printnum(all.meta$diab_ci_lb)
all.meta$diab_ci_ub = printnum(all.meta$diab_ci_ub)
all.meta$nodiab_estimate = printnum(all.meta$nodiab_estimate)
all.meta$nodiab_ci_lb = printnum(all.meta$nodiab_ci_lb)
all.meta$nodiab_ci_ub = printnum(all.meta$nodiab_ci_ub)

all.meta = all.meta %>%
  mutate(diab_ci = paste0(diab_estimate, " [",
                              diab_ci_lb, ", ",
                              diab_ci_ub, "]"),
         nodiab_ci = paste0(nodiab_estimate, " [",
                              nodiab_ci_lb, ", ",
                              nodiab_ci_ub, "]"))
```

We format the average main effect from the meta-analyses.

```r
average.diabetes.srh = paste0(
  printnum(meta.srh.diabetes$b), " [",
  printnum(meta.srh.diabetes$ci.lb), ", ",
  printnum(meta.srh.diabetes$ci.ub), "]")

average.nodiabetes.srh = paste0(
  printnum(meta.srh.nodiabetes$b), " [",
  printnum(meta.srh.nodiabetes$ci.lb), ", ",
  printnum(meta.srh.nodiabetes$ci.ub), "]")

average.diabetes.smoker = paste0(
  printnum(meta.smoker.diabetes$b), " [",
  printnum(meta.smoker.diabetes$ci.lb), ", ",
  printnum(meta.smoker.diabetes$ci.ub), "]")

average.diabetes.smoker.OR = printnum(exp(meta.smoker.diabetes$b))

average.nodiabetes.smoker = paste0(
  printnum(meta.smoker.nodiabetes$b), " [",
  printnum(meta.smoker.nodiabetes$ci.lb), ", ",
  printnum(meta.smoker.nodiabetes$ci.ub), "]")

average.nodiabetes.smoker.OR = printnum(exp(meta.smoker.nodiabetes$b))

average.diabetes.heart = paste0(
  printnum(meta.heart.diabetes$b), " [",
  printnum(meta.heart.diabetes$ci.lb), ", ",
  printnum(meta.heart.diabetes$ci.ub), "]")

average.diabetes.heart.OR = printnum(exp(meta.heart.diabetes$b))

average.nodiabetes.heart = paste0(
```

```
  printnum(meta.heart.nodiabetes$b), " [",
  printnum(meta.heart.nodiabetes$ci.lb), ", ",
  printnum(meta.heart.nodiabetes$ci.ub), "]")

average.nodiabetes.heart.OR = printnum(exp(meta.heart.nodiabetes$b))
```

Now we can build the figure. First we find the horizontal limits of the graph.

```
extra.columns = all.meta %>%
  mutate(p_diabetes = paste0(round((n_diabetes/n_all)*100),"%"),
         study = toupper(study)) %>%
  dplyr::select(n_all, p_diabetes, diab_ci, diabetes.OR,
                nodiab_ci, nondiabetes.OR,
                interaction.OR)

num.ci = length(which(grepl("ci", names(extra.columns))))

# find plot limits
max.ci = max(all.meta$interaction_estimate+1.96*all.meta$interaction_std.error)
min.ci = min(all.meta$interaction_estimate-1.96*all.meta$interaction_std.error)
range = max.ci-min.ci
# the lower bound must give room for all the extra columns we have
# most columns need only a little space, but columns containing confidence intervals need more
lower = min.ci-(range*(.5*ncol(extra.columns) + .5*num.ci))
# the upper bound gives us a little space
upper = max.ci+range
```

Next we figure out where the extra columns go. We do so by dividing up the space between the minimum CI and the lower bound.

```
#how much room needed
needed.space = ncol(extra.columns) + num.ci

pos = min.ci-lower
pos = pos/(needed.space + 1)
column.position = 1:ncol(extra.columns)
for(i in 1:length(column.position)){
  if(grepl("ci", names(extra.columns)[i])) {
    column.position[(i):ncol(extra.columns)] = column.position[(i):ncol(extra.columns)] + .5
    }
}

positions = lower + pos*column.position + pos
```

Now we calculated the needed space along the y-axis. First we determine how many extra rows we need between each ”set” of analyses. We need two extra rows for specific information: the weighted average estimates and a label. From there, we added extra rows until the figure looked appealing. We ended up with 5 extra rows.

From there, we figure out which rows will be filled with each set of analyses. It’s important to remember that rows start from the bottom and as you move up the figure, the row number increases.

```
extra.space = 5

# which rows are for heart disease
total.heart = nrow(heart.data)
```

```
rows.heart = c(1, total.heart)

# which rows are for smokers
total.smoker = nrow(smoker.data)
# skip row for diabetes summary, space, and heart title
rows.smoker = c(rows.heart[2] + extra.space, rows.heart[2] + extra.space + total.smoker - 1)

# which rows are for self-rated health
total.srh = nrow(srh.data)
# skip row for hbp summary, space, title
rows.srh = c(rows.smoker[2] + extra.space, rows.smoker[2] + extra.space + total.srh - 1)
```

The last step before beginning the plot is to set the font size and formatting. ‘font = 1‘ is the non-bold, non-italic font.

```
cex.value = .55
par(font = 1)
```

The final chunck builds the forest plot. Comments are included throughout as a guide.

```
pdf(here("Study 1/tables and figures/figures/meta.pdf"),width = 15*2/3, height = 10*2/3)
forest(all.meta$interaction_estimate, #estimate
       all.meta$interaction_std.error^2, #variance
       xlim = c(lower, upper), #limits of x-axis, full figure
       ylim = c(-1, nrow(all.meta) + extra.space*2 + 2), # limits of y-axis
       cex = cex.value, #font size
       slab = all.meta$study, #study label
       # which rows do I fill in. Remember, data frame goes from top to bottom, but row numbers on
       # figure go from bottom to top
       rows = c(rows.srh[2]:rows.srh[1],
                rows.smoker[2]:rows.smoker[1],
                rows.heart[2]:rows.heart[1]),
       # what extra information is added?
       ilab = cbind(extra.columns[,1], extra.columns[,2], extra.columns[,3],
                    extra.columns[,4], extra.columns[,5], printnum(extra.columns[,6]),
                    extra.columns[,7]),
       # where does extra information go?
       ilab.xpos = positions[1:7])

# add weighted average effects polygon
addpoly(meta.srh.interaction, row=rows.srh[1]-1,
        cex = cex.value, mlab = "", col = "blue")
addpoly(meta.smoker.interaction, row=rows.smoker[1]-1,
        cex = cex.value, mlab = "", col = "blue")
addpoly(meta.heart.interaction, row=rows.heart[1]-1,
        cex = cex.value, mlab = "", col = "blue")

### add text with Q-value, dfs, p-value, and I^2 statistic for subgroups
text(x = lower, y = rows.srh[1]-2.25, pos=4, cex = cex.value,
     bquote(paste("RE Model for self-rated health (Q = ",
                  .(formatC(meta.srh.interaction$QE, digits=2, format="f")),
                  ", df = ", .(meta.srh.interaction$k -
                               meta.srh.interaction$p),
                  ", p = ", .(formatC(meta.srh.interaction$QEp, digits=2, format="f")), "; ", I^2, " = ",
                  .(formatC(meta.srh.interaction$I2, digits=1, format="f")), "%)")))
```

```
text(x = lower, y = rows.smoker[1]-2.25, pos=4, cex = cex.value,
     bquote(paste("RE Model for smoking status (Q = ",
                  .(formatC(meta.smoker.interaction$QE, digits=2, format="f")),
                  ", df = ", .(meta.smoker.interaction$k -
                               meta.smoker.interaction$p),
                  ", p = ", .(formatC(meta.smoker.interaction$QEp, digits=2, format="f")), "; ", I^2, " = ",
                  .(formatC(meta.smoker.interaction$I2, digits=1, format="f")), "%)")))

text(x = lower, y = rows.heart[1]-2.25, pos=4, cex = cex.value,
     bquote(paste("RE Model for heart disease status (Q = ",
                  .(formatC(meta.heart.interaction$QE, digits=2, format="f")),
                  ", df = ", .(meta.heart.interaction$k -
                               meta.heart.interaction$p),
                  ", p = ", .(formatC(meta.heart.interaction$QEp, digits=2, format="f")), "; ", I^2, " = ",
                  .(formatC(meta.heart.interaction$I2, digits=1, format="f")), "%)")))
par(font = 2)

# add "overall"" label to each set of analyses
text(lower, y = c(rows.srh[1]-1, rows.smoker[1]-1, rows.heart[1]-1),
     pos = 4,
     cex = cex.value,
     "Overall")

# add overall sample sizes for each set of analyses
text(x = positions[which(names(extra.columns) == "n_all")],
     y = rows.srh[1]-1,
     cex = cex.value,
     printnum(sum(srh.data$n_all), format = "d"))
text(x = positions[which(names(extra.columns) == "n_all")],
     y = rows.smoker[1]-1,
     cex = cex.value,
     printnum(sum(smoker.data$n_all), format = "d"))
text(x = positions[which(names(extra.columns) == "n_all")],
     y = rows.heart[1]-1,
     cex = cex.value,
     printnum(sum(heart.data$n_all), format = "d"))

# add weighted average simple slopes for each set of analyses
text(x = positions[which(names(extra.columns) == "diab_ci")],
     y = rows.srh[1]-1, cex = cex.value, average.diabetes.srh)
text(x = positions[which(names(extra.columns) == "diab_ci")],
     y = rows.smoker[1]-1, cex = cex.value, average.diabetes.smoker)
text(x = positions[which(names(extra.columns) == "diab_ci")],
     y = rows.heart[1]-1, cex = cex.value, average.diabetes.heart)
text(x = positions[which(names(extra.columns) == "diabetes.OR")],
     y = c(rows.smoker[1]-1, rows.heart[1]-1), cex = cex.value,
     c(printnum(exp(meta.smoker.diabetes$b)), printnum(exp(meta.heart.diabetes$b))))

text(x = positions[which(names(extra.columns) == "nodiab_ci")],
     y = rows.srh[1]-1, cex = cex.value, average.nodiabetes.srh)
text(x = positions[which(names(extra.columns) == "nodiab_ci")],
     y = rows.smoker[1]-1, cex = cex.value, average.nodiabetes.smoker)
text(x = positions[which(names(extra.columns) == "nodiab_ci")],
     y = rows.heart[1]-1, cex = cex.value, average.nodiabetes.heart)
text(x = positions[which(names(extra.columns) == "nondiabetes.OR")],
     y = c(rows.smoker[1]-1, rows.heart[1]-1), cex = cex.value,
```

```r
     c(printnum(exp(meta.smoker.nodiabetes$b)), printnum(exp(meta.heart.nodiabetes$b))))

text(x = positions[which(names(extra.columns) == "interaction.OR")],
     y = c(rows.smoker[1]-1, rows.heart[1]-1), cex = cex.value,
     c(printnum(exp(meta.smoker.interaction$b)), printnum(exp(meta.heart.interaction$b))))

# column labels
text(lower, nrow(all.meta) + extra.space*2 + 1,
     "Study", cex = cex.value, pos = 4)
text(upper, nrow(all.meta) + extra.space*2 + 1,
     "Interaction Coefficient Estimate [95% CI]", cex = cex.value, pos=2)
text(positions[which(names(extra.columns) == "n_all")], nrow(all.meta) + extra.space*2 + 1,
     "Sample Size", cex = cex.value)
text(positions[which(names(extra.columns) == "diab_ci")], nrow(all.meta) + extra.space*2 + 1,
     "Adults with Diabetes", cex = cex.value)
text(positions[which(names(extra.columns) == "nodiab_ci")], nrow(all.meta) + extra.space*2 + 1,
     "Adults without Diabetes", cex = cex.value)
text((positions[which(names(extra.columns) == "diab_ci")] + positions[which(names(extra.columns) == "nodiab_ci")])/2

text(positions[grepl("OR", names(extra.columns))], nrow(all.meta) + extra.space*2 + 1,
     cex = cex.value, "OR")
text(positions[grepl("interaction.OR", names(extra.columns))], nrow(all.meta) + extra.space*2 + 2,
     cex = cex.value, "Interaction")

#bold and italic font, plus bigger text
par(font = 4)

# outcome labels
text(lower, rows.srh[2] + 1,
     "Self-Rated Health", cex = cex.value, pos = 4)
text(lower, rows.smoker[2] + 1,
     "Smoking Status", cex = cex.value, pos = 4)
text(lower, rows.heart[2] + 1,
     "Heart Disease Status", cex = cex.value, pos = 4)
dev.off()

## pdf
##   2
```

5.5 Plot

We pull the data from the output objects into three data frames, one for each outcome.

```
# self-rated health
plot.srh = data.frame(study = study.names)

plot.srh$n = unlist(
  lapply(X = study.names,FUN = function(x)
      length(get(paste0(x,"_out"))$regression$interaction$srh$residuals)))

plot.srh$data = lapply(X = study.names,
                         FUN = function(x) get(paste0(x,"_out"))$plotdata$srh)

# smoking status
plot.smoker = data.frame(study = smoker.names)

plot.smoker$n = unlist(
  lapply(X = smoker.names, FUN = function(x)
      length(get(paste0(x,"_out"))$regression$interaction$smoker$residuals)))

plot.smoker$data = lapply(X = smoker.names,
                         FUN = function(x) get(paste0(x,"_out"))$plotdata$smoker)


#heart disease status
plot.heart = data.frame(study = heart.names)

plot.heart$n = unlist(
  lapply(X = heart.names, FUN = function(x)
      length(get(paste0(x,"_out"))$regression$interaction$heart$residuals)))

plot.heart$data = lapply(X = heart.names,
                         FUN = function(x) get(paste0(x,"_out"))$plotdata$heart)
```

Next we unnest these data frames and add a variable specfiying the outcome. We also change the group variable to numeric, since it is binary. Finally, for binary outcomes, we calculate the logodds from the predicted value, since the default predicted value from the plot model function is the probability.

```
plot.srh = plot.srh %>%
  unnest() %>%
  mutate(group = factor(group, labels = c("No Diabetes", "Diabetes")))

plot.smoker = plot.smoker %>%
  unnest() %>%
  mutate(group = factor(group, labels = c("No Diabetes", "Diabetes")))

plot.heart = plot.heart %>%
  unnest() %>%
   mutate(group = factor(group, labels = c("No Diabetes", "Diabetes")))
```

We plot the individual study predicted values, as well as the weighted average. These values are shown in Figure ??.

```
plot.srh %>%
  ggplot(aes(x = x, y = predicted)) +
```

```
  geom_line(aes(color = study)) +
  geom_smooth(method = "lm", aes(weight = n), se = TRUE, color = "black") +
  scale_x_continuous("Purpose in Life") +
  scale_y_continuous("Self-rated health") +
  facet_grid(.~group, scale = "free") +
  theme_bw() +
  theme(legend.position = "bottom")

## `geom_smooth()` using formula = 'y ~ x'
```

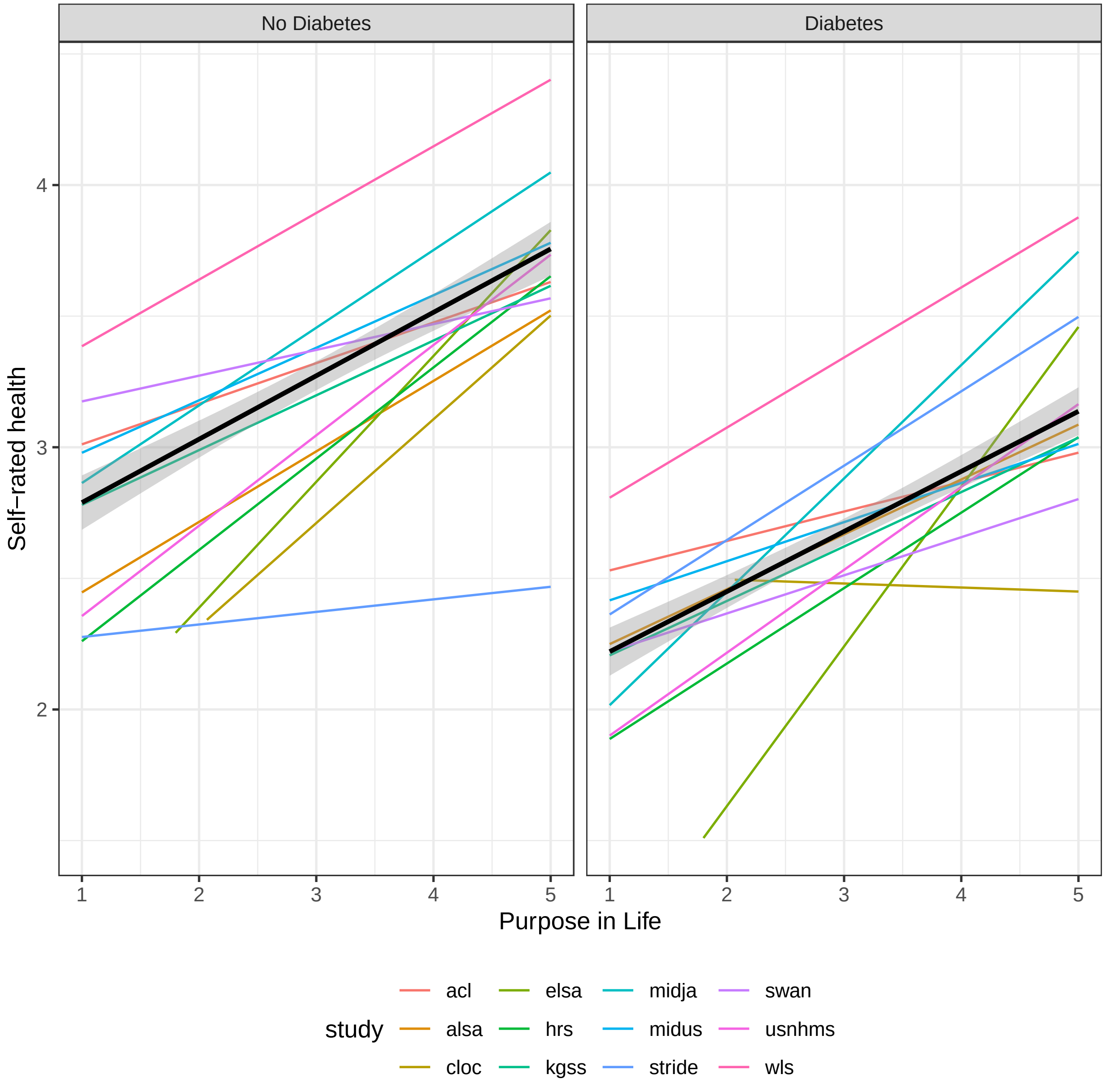

```r
plot.smoker %>%
  ggplot(aes(x = x, y = predicted)) +
  geom_line(aes(color = study)) +
  geom_smooth(aes(weight = n),
              method = "lm", formula = y ~ I(exp(x)/(1+exp(x))),
              size = 1, color = "black") +
  scale_x_continuous("Purpose in Life") +
  scale_y_continuous("Smoking status") +
  facet_grid(.~group, scale = "free") +
  theme_bw() +
  theme(legend.position = "bottom")

## Warning:  Using `size` aesthetic for lines was deprecated in ggplot2 3.4.0.
## i Please use `linewidth` instead.
## This warning is displayed once every 8 hours.
## Call `lifecycle::last_lifecycle_warnings()` to see where this warning was
## generated.
```

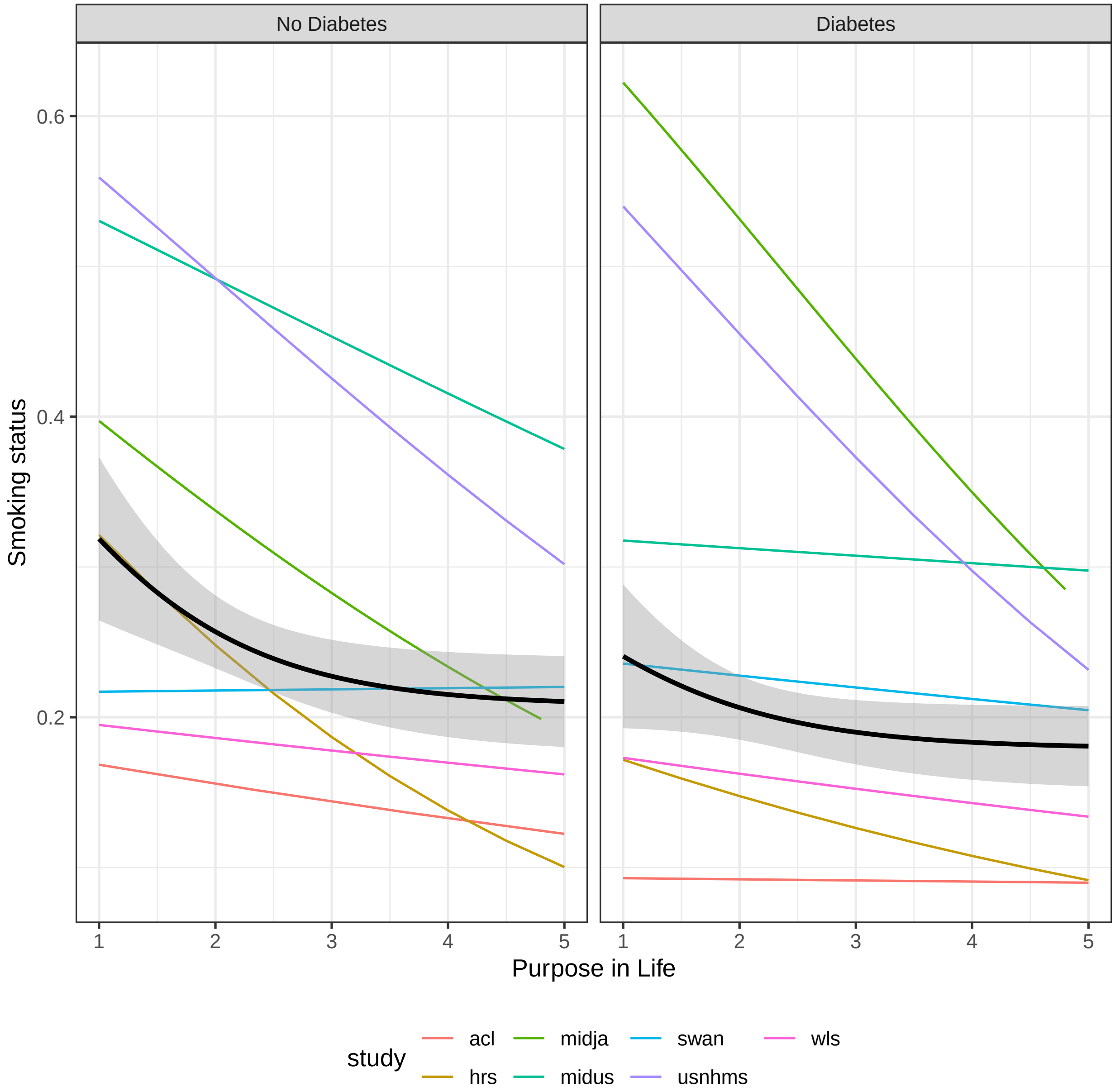


```
plot.heart %>%
  ggplot(aes(x = x, y = predicted)) +
  geom_line(aes(color = study)) +
  geom_smooth(aes(weight = n),
              method = "lm", formula = y ~ I(exp(x)/(1+exp(x))),
              size = 1, color = "black") +
  scale_x_continuous("Purpose in Life") +
  scale_y_continuous("Heart disease status") +
  facet_grid(.~group, scale = "free") +
  theme_bw() +
  theme(legend.position = "bottom")
```

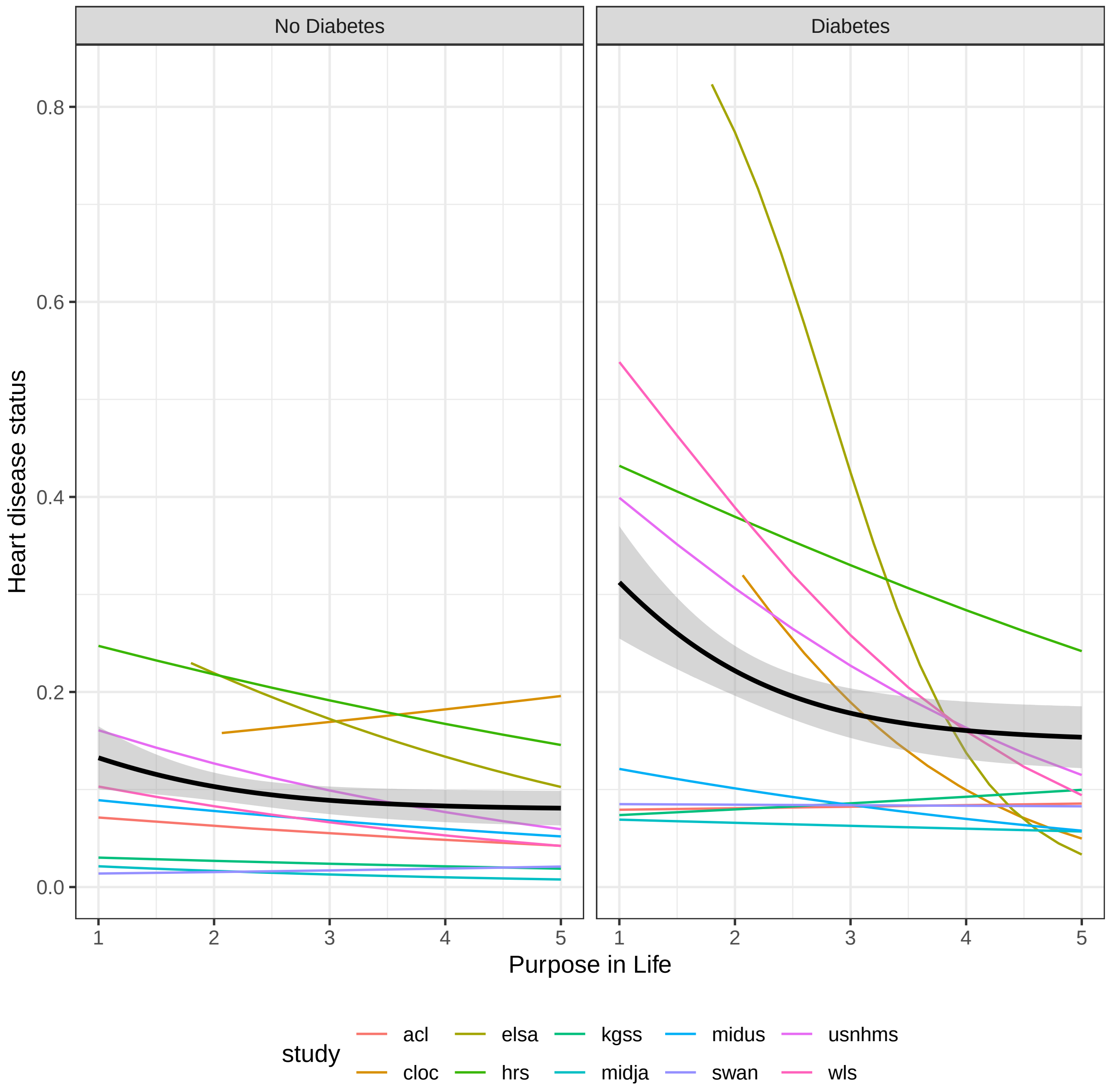

6 Moderators of interaction

We add additional information to the study levels data frame.

```
study.level$year[study.level$study == "acl"] = 2002
study.level$year[study.level$study == "alsa"] = 1994
study.level$year[study.level$study == "cloc"] = 1989
study.level$year[study.level$study == "elsa"] = 2004
study.level$year[study.level$study == "hrs"] = 2006
study.level$year[study.level$study == "kgss"] = 2009
study.level$year[study.level$study == "midja"] = 2008
```

```
study.level$year[study.level$study == "midus"] = 1994
study.level$year[study.level$study == "stride"] = 2004
study.level$year[study.level$study == "swan"] = 1996
study.level$year[study.level$study == "usnhms"] = 2005
study.level$year[study.level$study == "wls"] = 1992

study.level$american = ifelse(study.level$study %in%
                               c("alsa","elsa","kgss","midja"),0,1)
```

```
moderator.data = data.frame(out = c("srh","smoker","heart"),
                            age = "age", year = "year",
                            number_items = "number_items", american = "american")
moderator.data$data = list(srh.data, smoker.data, heart.data)

moderator.data = moderator.data %>%
  gather(key = "mods", value = "value", -data, -out) %>%
  dplyr::select(-value) %>%
  mutate(data = map(data, ~full_join(., study.level)))

moderator.data = moderator.data %>%
  mutate(rma.diab = map2(.x = data, .y = mods,
                         .f = ~rma(yi = diab_estimate,
                                   sei = diab_std.error,
                                   ni = n_diabetes,
                                   method = "REML",
                                   mods = as.formula(paste0("~", .y)),
                                   data = .x,
                                   control=list(maxiter=1000, stepadj=0.5))),
         rma.nond = map2(.x = data, .y = mods,
                         .f = ~rma(yi = nodiab_estimate,
                                   sei = nodiab_std.error,
                                   ni = n_nodiabetes,
                                   method = "REML",
                                   mods = as.formula(paste0("~", .y)),
                                   data = .x,
                                   control=list(maxiter=1000, stepadj=0.5))),
         rma.intr = map2(.x = data, .y = mods,
                         .f = ~rma(yi = interaction_estimate,
                                   sei = interaction_std.error,
                                   ni = n_all,
                                   method = "REML",
                                   mods = as.formula(paste0("~", .y)),
                                   data = .x,
                                   control=list(maxiter=1000, stepadj=0.5)))) %>%
  gather(key = "model", value = "value", which(grepl("rma", names(.)))) %>%
  mutate(beta = map(value, "beta"),
         se = map(value, "se"),
         ci.lb = map(value, "ci.lb"),
         ci.ub = map(value, "ci.ub"),
         zval = map(value, "zval"),
         pval = map(value, "pval")) %>%
```

```r
  dplyr::select(-data, -value) %>%
  mutate(beta = map_dbl(beta, ~unlist(.x)[2]),
         se = map_dbl(se, ~unlist(.x)[2]),
         ci.lb = map_dbl(ci.lb, ~unlist(.x)[2]),
         ci.ub = map_dbl(ci.ub, ~unlist(.x)[2]),
         zval = map_dbl(zval, ~unlist(.x)[2]),
         pval = map_dbl(pval, ~unlist(.x)[2]),
         model = factor(model, levels = c("rma.diab", "rma.nond", "rma.intr")),
         mods = gsub("_", " of ", mods),
         padj = p.adjust(pval, method = "holm"))

moderator.data %>% arrange(desc(out), model) %>%
  dplyr::select(-out, -model) %>%
  kable(.,
        caption = "Study-level moderators",
        booktabs = T, escape = F, digits = 2, format = "latex", longtable = T,
        col.names = c("Moderator", "Coefficient", "SE", "lower", "upper", "Z", "raw", "adjusted")) %>%
  add_header_above(c(" " = 3, "Confidence Interval" = 2, " " = 1, "p value" = 2)) %>%
  group_rows("Self-rated health: Effect of purpose (diabetes only)", 1,4) %>%
  group_rows("Self-rated health: Effect of purpose (no diabetes only)", 5,8) %>%
  group_rows("Self-rated health: Interaction of purpose and diabetes", 9,12) %>%
  group_rows("Smoking status: Effect of purpose (diabetes only)", 13,16) %>%
  group_rows("Smoking status: Effect of purpose (no diabetes only)", 17,20) %>%
  group_rows("Smoking status: Interaction of purpose and diabetes", 21,24) %>%
  group_rows("Heart disease status: Effect of purpose (diabetes only)", 25,28) %>%
  group_rows("Heart disease status: Effect of purpose (no diabetes only)", 29,32) %>%
  group_rows("Heart disease status: Interaction of purpose and diabetes", 33,36)
```

Table 7: Study-level moderators

			Confidence Interval			p value	
Moderator	Coefficient	SE	lower	upper	Z	raw	adjusted
**Self-rated health: Effect of purpose (diabetes only)**							
age	0.03	0.03	-0.03	0.08	0.99	0.32	1.00
year	0.01	0.00	0.00	0.02	1.18	0.24	1.00
number of items	0.03	0.01	0.02	0.05	4.90	0.00	0.00
american	-0.07	0.08	-0.24	0.10	-0.81	0.42	1.00
**Self-rated health: Effect of purpose (no diabetes only)**							
age	0.06	0.02	0.02	0.10	3.02	0.00	0.09
year	0.00	0.01	-0.01	0.01	0.29	0.78	1.00
number of items	0.03	0.01	0.02	0.05	4.54	0.00	0.00
american	-0.08	0.07	-0.21	0.06	-1.09	0.28	1.00
**Self-rated health: Interaction of purpose and diabetes**							
age	-0.04	0.02	-0.08	-0.01	-2.51	0.01	0.38
year	0.00	0.00	-0.01	0.00	-1.23	0.22	1.00
number of items	0.00	0.01	-0.02	0.01	-0.59	0.56	1.00
american	-0.03	0.07	-0.15	0.10	-0.40	0.69	1.00
**Smoking status: Effect of purpose (diabetes only)**							
age	-0.08	0.06	-0.19	0.04	-1.30	0.19	1.00

year	-0.01	0.01	-0.03	0.00	-1.53	0.13	1.00
number of items	-0.04	0.02	-0.08	0.01	-1.59	0.11	1.00
american	0.16	0.49	-0.79	1.12	0.33	0.74	1.00
**Smoking status: Effect of purpose (no diabetes only)**							
age	-0.13	0.05	-0.23	-0.02	-2.42	0.02	0.48
year	-0.02	0.01	-0.03	0.00	-2.59	0.01	0.32
number of items	-0.03	0.02	-0.07	0.01	-1.45	0.15	1.00
american	0.11	0.22	-0.31	0.54	0.52	0.60	1.00
**Smoking status: Interaction of purpose and diabetes**							
age	0.11	0.06	-0.01	0.23	1.73	0.08	1.00
year	0.01	0.01	0.00	0.03	1.46	0.14	1.00
number of items	0.01	0.03	-0.04	0.06	0.36	0.72	1.00
american	0.19	0.50	-0.79	1.16	0.37	0.71	1.00
**Heart disease status: Effect of purpose (diabetes only)**							
age	-0.02	0.11	-0.23	0.18	-0.22	0.82	1.00
year	0.01	0.01	-0.01	0.04	0.92	0.36	1.00
number of items	-0.05	0.03	-0.11	0.00	-1.84	0.07	1.00
american	-0.09	0.26	-0.61	0.42	-0.36	0.72	1.00
**Heart disease status: Effect of purpose (no diabetes only)**							
age	-0.05	0.05	-0.14	0.05	-1.00	0.32	1.00
year	0.00	0.01	-0.02	0.01	-0.39	0.70	1.00
number of items	-0.03	0.01	-0.05	0.00	-2.01	0.04	1.00
american	-0.03	0.13	-0.29	0.23	-0.23	0.82	1.00
**Heart disease status: Interaction of purpose and diabetes**							
age	0.00	0.07	-0.14	0.13	-0.05	0.96	1.00
year	0.02	0.01	0.00	0.03	1.69	0.09	1.00
number of items	-0.03	0.03	-0.08	0.02	-1.07	0.28	1.00
american	-0.22	0.22	-0.65	0.20	-1.02	0.31	1.00

7 Measures of purpose

```
variable_list <- read_excel(here("Study 1/variable list working.xlsx"))

## New names:
## * `Dataset` -> `Dataset...3`
## * `Dataset` -> `Dataset...10`

variable_list = variable_list %>%
  rename(Dataset = Dataset...3,
         file = Dataset...10) %>%
  filter(Construct == "Purpose") %>%
  dplyr::select(-`Study 2`)%>%
  group_by(Dataset) %>%
  arrange(`Wave/Year`) %>%
  filter(`Wave/Year` == `Wave/Year`[1]) %>%
  dplyr::select(Dataset, Item, Ryff) %>%
  mutate(value = "x") %>%
  spread(key = "Dataset", value = "value") %>%
  arrange(Ryff)

totals = apply(variable_list, MARGIN = 2,
                           function(x) length(which(!is.na(x))))

newnames = c("Item",  "Ryff",  "ACL",  "ALSA",  "CLOC",  "ELSA",  "HRS",  "KGSS",  "MIDJA",  "MIDUS",  "[illegible]

variable_list %>%
kable(.,
        caption = "Items used to assess purpose \\label{purpose}",
        booktabs = T, escape = FALSE, format = "latex", longtable = T,
        align = c("l", rep("c", length(totals)+1)),
      col.names = c("Total", " ", as.numeric(totals[-c(1,2)]))) %>%
  kable_styling("striped") %>%
  add_header_above(newnames) %>%
  column_spec(1, width = "15em") %>%
  landscape()
```

Table 8: Items used to assess purpose

Item	Ryff	ACL	ALSA	CLOC	ELSA	HRS	KGSS	MIDJA	MIDUS	STRIDE	SWAN	USNHMS	WLS-G	WLS-S
Total		1	3	5	7	7	4	7	3	4	3	7	7	7
I tend to focus on the present because the future nearly always brings me problems.	Full												x	x
I used to set goals for myself but that now seems like a waste.	Full		x										x	x
Some people wander aimlessly through life, but I am not one of them.	Full				x			x	x	x				
I believe I can find the purpose of life, i.e., a reason to live for.	No						x							
I believe that I have control over my life and destiny.	No						x							
I have a mission or purpose in life.	No										x			
I have future plans I am looking forward to carrying out.	No						x							
I have something meaningful in my life that helps me get through difficult times.	No										x			
I still have many things left to do.	No						x							
My faith sustains me.	No										x			
I am an active person in carrying out the plans I set for myself.	SS-7				x	x						x	x	x
I don't have a good sense of what it is I'm trying to accomplish in life.	SS-7				x	x		x				x	x	x
I enjoy making plans for the future and working to make them a reality.	SS-7		x	x	x	x		x				x	x	x
I have a sense of direction and purpose in my life.	SS-7	x	x	x	x	x		x		x		x		
I live life one day at a time and don't really think about the future.	SS-7				x	x		x	x	x		x		

I sometimes feel as if I've done all there is to do in life.	SS-7		x	x	x	x	x	x	x	x
My daily activities often seem trivial and unimportant to me.	SS-7	x		x	x			x	x	x
I am not a person who wanders aimlessly through life.	Sort of	x								
I have a good sense of what I'm trying to accomplish in life.	Sort of	x								

8 Session Information

The analyses displayed here were run on a computer with the following settings:

```
## setting  value
## version  R version 4.2.3 (2023-03-15)
## os       macOS Big Sur ... 10.16
## system   x86_64, darwin17.0
## ui       X11
## language (EN)
## collate  en_US.UTF-8
## ctype    en_US.UTF-8
## tz       America/Los_Angeles
## date     2023-04-24
## pandoc   2.19.2 @ /Applications/RStudio.app/Contents/Resources/app/quarto/bin/tools/ (via rmarkdown)
```

The following packages were used:

	package	loadedversion	date	source
abind	abind	1.4-5	2016-07-21	CRAN (R 4.2.0)
apaTables	apaTables	2.0.8	2021-01-04	CRAN (R 4.2.0)
arm	arm	1.12-2	2021-10-15	CRAN (R 4.2.0)
assertthat	assertthat	0.2.1	2019-03-21	CRAN (R 4.2.0)
backports	backports	1.4.1	2021-12-13	CRAN (R 4.2.0)
bayestestR	bayestestR	0.13.1	2023-04-07	CRAN (R 4.2.0)
blme	blme	1.0-5	2021-01-05	CRAN (R 4.2.0)
boot	boot	1.3-28.1	2022-11-22	CRAN (R 4.2.3)
broom	broom	1.0.4	2023-03-11	CRAN (R 4.2.0)
broom.mixed	broom.mixed	0.2.9.4	2022-04-17	CRAN (R 4.2.0)
cachem	cachem	1.0.6	2021-08-19	CRAN (R 4.2.0)
callr	callr	3.7.3	2022-11-02	CRAN (R 4.2.0)
cellranger	cellranger	1.1.0	2016-07-27	CRAN (R 4.2.0)
cli	cli	3.6.1	2023-03-23	CRAN (R 4.2.0)
coda	coda	0.19-4	2020-09-30	CRAN (R 4.2.0)
codetools	codetools	0.2-19	2023-02-01	CRAN (R 4.2.3)
colorspace	colorspace	2.1-0	2023-01-23	CRAN (R 4.2.0)
crayon	crayon	1.5.2	2022-09-29	CRAN (R 4.2.0)
datawizard	datawizard	0.7.1	2023-04-03	CRAN (R 4.2.0)
DBI	DBI	1.1.3	2022-06-18	CRAN (R 4.2.0)
dbplyr	dbplyr	2.2.1	2022-06-27	CRAN (R 4.2.0)
devtools	devtools	2.4.5	2022-10-11	CRAN (R 4.2.0)
digest	digest	0.6.31	2022-12-11	CRAN (R 4.2.0)
dplyr	dplyr	1.1.1	2023-03-22	CRAN (R 4.2.0)
effectsize	effectsize	0.8.3	2023-01-28	CRAN (R 4.2.0)
effsize	effsize	0.8.1	2020-10-05	CRAN (R 4.2.0)
ellipsis	ellipsis	0.3.2	2021-04-29	CRAN (R 4.2.0)
emmeans	emmeans	1.8.5	2023-03-08	CRAN (R 4.2.0)
estimability	estimability	1.4.1	2022-08-05	CRAN (R 4.2.0)
evaluate	evaluate	0.20	2023-01-17	CRAN (R 4.2.0)
fansi	fansi	1.0.4	2023-01-22	CRAN (R 4.2.0)
farver	farver	2.1.1	2022-07-06	CRAN (R 4.2.0)
fastmap	fastmap	1.1.0	2021-01-25	CRAN (R 4.2.0)
forcats	forcats	0.5.2	2022-08-19	CRAN (R 4.2.0)

foreach	foreach	1.5.2	2022-02-02	CRAN (R 4.2.0)
fs	fs	1.6.1	2023-02-06	CRAN (R 4.2.0)
furrr	furrr	0.3.1	2022-08-15	CRAN (R 4.2.0)
future	future	1.27.0	2022-07-22	CRAN (R 4.2.0)
gargle	gargle	1.2.0	2021-07-02	CRAN (R 4.2.0)
generics	generics	0.1.3	2022-07-05	CRAN (R 4.2.0)
ggeffects	ggeffects	1.2.1	2023-04-02	CRAN (R 4.2.0)
ggplot2	ggplot2	3.4.2	2023-04-03	CRAN (R 4.2.0)
globals	globals	0.16.1	2022-08-28	CRAN (R 4.2.0)
glue	glue	1.6.2	2022-02-24	CRAN (R 4.2.0)
googledrive	googledrive	2.0.0	2021-07-08	CRAN (R 4.2.0)
googlesheets4	googlesheets4	1.0.1	2022-08-13	CRAN (R 4.2.0)
gtable	gtable	0.3.3	2023-03-21	CRAN (R 4.2.0)
haven	haven	2.5.1	2022-08-22	CRAN (R 4.2.0)
here	here	1.0.1	2020-12-13	CRAN (R 4.2.0)
highr	highr	0.10	2022-12-22	CRAN (R 4.2.0)
hms	hms	1.1.2	2022-08-19	CRAN (R 4.2.0)
htmltools	htmltools	0.5.3	2022-07-18	CRAN (R 4.2.0)
htmlwidgets	htmlwidgets	1.5.4	2021-09-08	CRAN (R 4.2.0)
httpuv	httpuv	1.6.6	2022-09-08	CRAN (R 4.2.0)
httr	httr	1.4.4	2022-08-17	CRAN (R 4.2.0)
insight	insight	0.19.1	2023-03-18	CRAN (R 4.2.0)
iterators	iterators	1.0.14	2022-02-05	CRAN (R 4.2.0)
jsonlite	jsonlite	1.8.4	2022-12-06	CRAN (R 4.2.0)
kableExtra	kableExtra	1.3.4	2021-02-20	CRAN (R 4.2.0)
knitr	knitr	1.42	2023-01-25	CRAN (R 4.2.0)
labeling	labeling	0.4.2	2020-10-20	CRAN (R 4.2.0)
later	later	1.3.0	2021-08-18	CRAN (R 4.2.0)
lattice	lattice	0.20-45	2021-09-22	CRAN (R 4.2.3)
lifecycle	lifecycle	1.0.3	2022-10-07	CRAN (R 4.2.0)
listenv	listenv	0.8.0	2019-12-05	CRAN (R 4.2.0)
lme4	lme4	1.1-32	2023-03-14	CRAN (R 4.2.0)
lubridate	lubridate	1.8.0	2021-10-07	CRAN (R 4.2.0)
magrittr	magrittr	2.0.3	2022-03-30	CRAN (R 4.2.0)
MASS	MASS	7.3-58.2	2023-01-23	CRAN (R 4.2.3)
mathjaxr	mathjaxr	1.6-0	2022-02-28	CRAN (R 4.2.0)
Matrix	Matrix	1.5-3	2022-11-11	CRAN (R 4.2.3)
memoise	memoise	2.0.1	2021-11-26	CRAN (R 4.2.0)
merTools	merTools	0.5.2	2020-06-23	CRAN (R 4.2.0)
metadat	metadat	1.2-0	2022-04-06	CRAN (R 4.2.0)
metafor	metafor	4.0-0	2023-03-19	CRAN (R 4.2.0)
mgcv	mgcv	1.8-42	2023-03-02	CRAN (R 4.2.3)
mime	mime	0.12	2021-09-28	CRAN (R 4.2.0)
miniUI	miniUI	0.1.1.1	2018-05-18	CRAN (R 4.2.0)
minqa	minqa	1.2.5	2022-10-19	CRAN (R 4.2.0)
mnormt	mnormt	2.1.0	2022-06-07	CRAN (R 4.2.0)
modelr	modelr	0.1.11	2023-03-22	CRAN (R 4.2.0)
multcomp	multcomp	1.4-20	2022-08-07	CRAN (R 4.2.0)
munsell	munsell	0.5.0	2018-06-12	CRAN (R 4.2.0)
mvtnorm	mvtnorm	1.1-3	2021-10-08	CRAN (R 4.2.0)
nlme	nlme	3.1-162	2023-01-31	CRAN (R 4.2.3)

nloptr	nloptr	2.0.3	2022-05-26	CRAN (R 4.2.0)
numDeriv	numDeriv	2016.8-1.1	2019-06-06	CRAN (R 4.2.0)
papaja	papaja	0.1.1	2022-07-05	CRAN (R 4.2.0)
parallelly	parallelly	1.32.1	2022-07-21	CRAN (R 4.2.0)
parameters	parameters	0.21.0	2023-04-19	CRAN (R 4.2.3)
performance	performance	0.10.3	2023-04-07	CRAN (R 4.2.0)
pillar	pillar	1.9.0	2023-03-22	CRAN (R 4.2.0)
pkgbuild	pkgbuild	1.4.0	2022-11-27	CRAN (R 4.2.0)
pkgconfig	pkgconfig	2.0.3	2019-09-22	CRAN (R 4.2.0)
pkgload	pkgload	1.3.2	2022-11-16	CRAN (R 4.2.0)
prettyunits	prettyunits	1.1.1	2020-01-24	CRAN (R 4.2.0)
processx	processx	3.8.1	2023-04-18	CRAN (R 4.2.3)
profvis	profvis	0.3.7	2020-11-02	CRAN (R 4.2.0)
promises	promises	1.2.0.1	2021-02-11	CRAN (R 4.2.0)
ps	ps	1.7.5	2023-04-18	CRAN (R 4.2.3)
psych	psych	2.2.5	2022-05-10	CRAN (R 4.2.0)
purrr	purrr	1.0.1	2023-01-10	CRAN (R 4.2.0)
R6	R6	2.5.1	2021-08-19	CRAN (R 4.2.0)
RColorBrewer	RColorBrewer	1.1-3	2022-04-03	CRAN (R 4.2.0)
Rcpp	Rcpp	1.0.10	2023-01-22	CRAN (R 4.2.0)
readr	readr	2.1.2	2022-01-30	CRAN (R 4.2.0)
readxl	readxl	1.4.1	2022-08-17	CRAN (R 4.2.0)
remotes	remotes	2.4.2	2021-11-30	CRAN (R 4.2.0)
reprex	reprex	2.0.2	2022-08-17	CRAN (R 4.2.0)
rlang	rlang	1.1.0	2023-03-14	CRAN (R 4.2.0)
rmarkdown	rmarkdown	2.16	2022-08-24	CRAN (R 4.2.1)
rprojroot	rprojroot	2.0.3	2022-04-02	CRAN (R 4.2.0)
rstudioapi	rstudioapi	0.14	2022-08-22	CRAN (R 4.2.0)
rvest	rvest	1.0.3	2022-08-19	CRAN (R 4.2.0)
sandwich	sandwich	3.0-2	2022-06-15	CRAN (R 4.2.0)
scales	scales	1.2.1	2022-08-20	CRAN (R 4.2.0)
sessioninfo	sessioninfo	1.2.2	2021-12-06	CRAN (R 4.2.0)
shiny	shiny	1.7.2	2022-07-19	CRAN (R 4.2.0)
sjlabelled	sjlabelled	1.2.0	2022-04-10	CRAN (R 4.2.0)
sjmisc	sjmisc	2.8.9	2021-12-03	CRAN (R 4.2.0)
sjPlot	sjPlot	2.8.14	2023-04-02	CRAN (R 4.2.0)
sjstats	sjstats	0.18.2	2022-11-19	CRAN (R 4.2.0)
snakecase	snakecase	0.11.0	2019-05-25	CRAN (R 4.2.0)
stringi	stringi	1.7.12	2023-01-11	CRAN (R 4.2.0)
stringr	stringr	1.5.0	2022-12-02	CRAN (R 4.2.0)
survival	survival	3.5-3	2023-02-12	CRAN (R 4.2.3)
svglite	svglite	2.1.0	2022-02-03	CRAN (R 4.2.0)
systemfonts	systemfonts	1.0.4	2022-02-11	CRAN (R 4.2.0)
TH.data	TH.data	1.1-1	2022-04-26	CRAN (R 4.2.0)
tibble	tibble	3.2.1	2023-03-20	CRAN (R 4.2.0)
tidyr	tidyr	1.3.0	2023-01-24	CRAN (R 4.2.0)
tidyselect	tidyselect	1.2.0	2022-10-10	CRAN (R 4.2.0)
tidyverse	tidyverse	1.3.2	2022-07-18	CRAN (R 4.2.0)
tinylabels	tinylabels	0.2.3	2022-02-06	CRAN (R 4.2.0)
tzdb	tzdb	0.3.0	2022-03-28	CRAN (R 4.2.0)
urlchecker	urlchecker	1.0.1	2021-11-30	CRAN (R 4.2.0)
usethis	usethis	2.1.6	2022-05-25	CRAN (R 4.2.0)

utf8	utf8	1.2.3	2023-01-31	CRAN (R 4.2.0)
vctrs	vctrs	0.6.1	2023-03-22	CRAN (R 4.2.3)
viridisLite	viridisLite	0.4.1	2022-08-22	CRAN (R 4.2.0)
webshot	webshot	0.5.3	2022-04-14	CRAN (R 4.2.0)
withr	withr	2.5.0	2022-03-03	CRAN (R 4.2.0)
xfun	xfun	0.38	2023-03-24	CRAN (R 4.2.0)
xml2	xml2	1.3.3	2021-11-30	CRAN (R 4.2.0)
xtable	xtable	1.8-4	2019-04-21	CRAN (R 4.2.0)
zoo	zoo	1.8-10	2022-04-15	CRAN (R 4.2.0)
