# Supplementary material for: Associations for Sense of Purpose with Smoking and Health Outcomes Among Adults with Diabetes
Source: Int J Behav Med. 2023 Jul 6;31(4):538–48. doi: 10.1007/s12529-023-10191-0 (PMC11269333; doi:10.1007/s12529-023-10191-0)
Supplement: Supplementary file 2 — Supplementary file2 (PDF 336 KB) [file 12529_2023_10191_MOESM2_ESM.pdf]

Supplementary Material: Code and Analysis for Study 2

This document contains all code used to clean and analyze data for Study 1. Results of analyses are saved as R objects and imported into the manuscript.

Contents

1 Data cleaning **2**
- 1.1 ACL . . . 2
- 1.2 ALSA . . . 2
- 1.3 ELSA . . . 3
- 1.4 HRS . . . 4
- 1.5 KGSS . . . 7
- 1.6 MIDJA . . . 9
- 1.7 MIDUS . . . 10
- 1.8 SWAN . . . 11
- 1.9 WLS . . . 13

2 Analyze individual studies **14**
- 2.1 Code for all studies . . . 14
- 2.2 Source script on all studies . . . 17

3 Descriptive Statistics **18**
- 3.1 Mean, standard deviation, min, max, alpha . . . 18

4 Regression Models: Purpose influencing trajectories **19**
- 4.1 Self-rated health . . . 20
- 4.2 Smoking status . . . 23
- 4.3 Heart condition status . . . 26

5 Meta-analysis **29**
- 5.1 Self-rated health . . . 29
- 5.2 Smoking status . . . 30
- 5.3 Heart condition status . . . 31
- 5.4 Summary . . . 33
- 5.5 Forest plot . . . 33

6 Moderation **39**
- 6.1 Self-rated health intercept . . . 39
- 6.2 Self-rated health slope . . . 43

7 Regression Models: Purpose influencing quadratic trajectories **49**
- 7.1 Self-rated health . . . 49
- 7.2 Smoking status . . . 52
- 7.3 Heart Condition status . . . 55

8 Session Information **58**

1 Data cleaning

1.1 ACL

```
load(here("Study 2/raw data/ACL/04690-0001-Data.rda"))
acl <- da04690.0001; rm(da04690.0001)

acl <- acl %>%
  dplyr::select(
         id = V1,
         purpose = V12561,
         diabetes = V12286,
         srh_0 = V12236,
         smoker_0 = V13003,
         heart_0 = V12297,
         srh_1 = V15822,
         smoker_1 = V15835,
         heart_1 = V15933,
         gender = V1801,
         white = V2059,
         edu1 = V2046,
         edu2 = V2047,
         edu3 = V2048,
         edu4 = V2049,
         edu5 = V2050,
         birthyr = V1645,
         interviewyr_0 = V12005,
         interviewyr_1 = V15010) %>%
  gather(key = "variable", value = "value", which(grepl("_", names(.)))) %>%
  separate(col = "variable", into = c("variable", "wave")) %>%
  spread(key = "variable", value = "value") %>%
  group_by(id) %>%
  mutate(interviewyr = substr(interviewyr, nchar(interviewyr)-4+1, nchar(interviewyr)),
         interviewyr = as.numeric(interviewyr),
         time = interviewyr - max(interviewyr),
         b.age = min(interviewyr) - birthyr)

#make variables numeric
acl$purpose <- as.numeric(acl$purpose)
acl$diabetes <- as.numeric(acl$diabetes)
acl$gender <- as.numeric(acl$gender)

acl$srh <- as.numeric(as.factor(acl$srh))
acl$smoker <- as.numeric(as.factor(acl$smoker))
acl$heart <- as.numeric(as.factor(acl$heart))


# reverse code purpose and self-rated health
acl$purpose <- acl$purpose*-1 + 5
acl$srh <- acl$srh*-1 + 6

# change 1 (yes) to 0 and 2 (no) to 1
acl <- scrub(acl, where = c("diabetes","smoker","heart"), isvalue = 2, newvalue = 0)

# make gender 0 = male 1 = female
acl$gender <- acl$gender - 1

#reverse race variable
acl$race = ifelse(acl$white == 1, 0, 1)
#create single education variable
acl$edu = ifelse(acl$edu1 == 1, 8, NA)
acl$edu = ifelse(acl$edu2 == 1, 10, acl$edu)
acl$edu = ifelse(acl$edu3 == 1, 12, acl$edu)
acl$edu = ifelse(acl$edu4 == 1, 14, acl$edu)
acl$edu = ifelse(acl$edu5 == 1, 16, acl$edu)



save(acl, file=here("Study 2/created data/acl.Rdata"))
```

1.2 ALSA

```
#load raw data
alsa1 <- read_por(here("Study 2/raw data/ALSA/06707-0003-Data.por"))
alsa3 <- read_por(here("Study 2/raw data/ALSA/06707-0007-Data.por"))
alsa4 <- read_por(here("Study 2/raw data/ALSA/06707-0008-Data.por"))
alsa5 <- read_por(here("Study 2/raw data/ALSA/06707-0010-Data.por"))

alsa4 = subset(alsa4, select = c(SEQNUM, SRHLTHW4))
alsa5 = subset(alsa5, select = c(SEQNUM, SRHLTHW5))

alsa = full_join(alsa1, alsa3) %>%
  full_join(alsa4) %>%
  full_join(alsa5)

rm(list = c("alsa1", "alsa3", "alsa4", "alsa5"))

#select variables in main data set

alsa <- alsa %>%
  dplyr::select(
         id = SEQNUM,
         birthday = BIRTHDAT,
         gender = SEXW3,
         edu = FORMSCW3,
         purpose1 = DIRPURW3,
         purpose2 = GOALWSW3,
         purpose3 = FUTPLNW3,
         srh_0 = SRHW3,
         srh_1 = SRHLTHW4,
         srh_2 = SRHLTHW5,
         diabetes1 = DIABETE,
         diabetes3 = DIABW3) %>%
  gather(key = "variable", value = "value", which(grepl("_", names(.)))) %>%
  separate(col = "variable", into = c("variable", "time"), sep = "_") %>%
  spread(key = "variable", value = "value") %>%
  mutate(time = as.numeric(time),
         time = time - max(time))

alsa$birthyear = substr(alsa$birthday,
                        nchar(alsa$birthday)-4+1,
                        nchar(alsa$birthday))
# there's one that doesn't make sense. remove
alsa <- scrub(alsa, where = "birthyear", isvalue = "9100")
alsa$birthyear = as.numeric(alsa$birthyear)
alsa$b.age = 1994 - alsa$birthyear

alsa = alsa %>%
  mutate(gender = gender-1, #make binary, 1 = female, 0 = male
         srh = srh*-1 + 6, # reverse code so 5 = excellent, 1 = poor
         diabetes1 = ifelse(diabetes1 == 1, 1, 0), #recode so that no is 0
         diabetes3 = ifelse(diabetes3 == 1, 1, 0)) #recode so that no is 0

# use information from two diabetes variables to create most complete and up-to-date diabetes status variable
alsa$diabetes = alsa$diabetes1
alsa$diabetes[alsa$diabetes3 == 1] = 1

alsa = dplyr::select(alsa, -diabetes1, - diabetes3)

save(alsa, file=here("Study 2/created data/alsa.Rdata"))
```

1.3 ELSA

```
#load raw data
h_elsa <- read_sav(here("Study 2/raw data/ELSA/h_elsa.sav"))
ryff_elsa <- read_sas(here("Study 2/raw data/ELSA/wave_2_ryff_data.sas7bdat"))

#dplyr::select and rename variables in each set
h_elsa <- h_elsa %>%
  dplyr::select(
         id = idauniq,
         diabetes = r2diabe,
         srh_2 = r2shlt,
         smoker_2 = r2smoken,
         heart_2 = r2hearte,
```

```
        interview_2 = r2iwindy,
        smoker_3 = r3smoken,
        heart_3 = r3hearte,
        interview_3 = r3iwindy,
        srh_4 = r4shlt,
        smoker_4 = r4smoken,
        heart_4 = r4hearte,
        interview_4 = r4iwindy,
        srh_5 = r5shlt,
        smoker_5 = r5smoken,
        heart_5 = r5hearte,
        interview_5 = r5iwindy,
        srh_6 = r6shlt,
        smoker_6 = r6smoken,
        heart_6 = r6hearte,
        interview_6 = r6iwindy,
        gender = ragender,
        b.age = r2agey,
        race = raracem,
        edu = raeduc_e) %>%
  gather(key = "variable", value = "value", which(grepl("_", names(.)))) %>%
  separate("variable", into = c("variable", "wave"), sep = "_") %>%
  spread(key = "variable", value = "value") %>%
  group_by(id) %>%
  mutate(baselineyr = interview[wave == 2],
        time = baselineyr-interview) %>%
  ungroup() %>%
  filter(!is.na(interview))

elsa <- ryff_elsa %>%
  dplyr::select(
        id = idauniq,
        purpose1 = RFWAND,
        purpose2 = RFONEDA,
        purpose3 = RFDONE,
        purpose4 = RFSENSE,
        purpose5 = RFACCOM,
        purpose6 = RFPLANS,
        purpose7 = RFACTIV) %>%
# merge together
  full_join(h_elsa)

#remove "don't know"
elsa <- scrub(elsa, isvalue = -1)

#reverse purpose items
purpose.items <- elsa[,which(grepl("purpose",names(elsa)))]
purpose.items <- reverse.code(keys = rep(-1, ncol(purpose.items)),
                              items = purpose.items)
elsa[,which(grepl("purpose",names(elsa)))] <- purpose.items

#reverse self rated health item
elsa$srh <- elsa$srh*-1 + 6

# make gender 0 = male 1 = female
elsa$gender <- elsa$gender - 1

# make race 0 = white, 1 = nonwhite
elsa$race <- ifelse(elsa$race == 1, 0, 1)

# make education 1-4 instead of 1, 3, 4, 5
elsa$edu <- ifelse(elsa$edu == 1, 1, elsa$edu-1)

#make diabetes numeric
elsa$diabetes <- as.numeric(elsa$diabetes)

save(elsa, file=here("Study 2/created data/elsa.Rdata"))
```

1.4 HRS

The HRS was the only study to measure purpose at different times for different participants. Consequently, additional care must be taken to match the first time a participant completed the purpose questionnaire with his or her health data.

```r
#load raw data
hrs <- read_sas(here("Study 2/raw data/HRS/rndhrs_p.sas7bdat"))
h06data <- read_sas(here("Study 2/raw data/HRS/h06f2b.sas7bdat"))
h08data <- read_sas(here("Study 2/raw data/HRS/h08f2a.sas7bdat"))
h10data <- read_sas(here("Study 2/raw data/HRS/hd10f5c.sas7bdat"))
h12data <- read_sas(here("Study 2/raw data/HRS/h12f1a.sas7bdat"))
h14data <- read_sas(here("Study 2/raw data/HRS/h14e1a.sas7bdat"))
h16data_lb<- read.SAScii(fn = here("Study 2/raw data/HRS/H16LB_R.da"),
                         sas_ri = here("Study 2/raw data/HRS/H16LB_R.sas"))
h16data_c<- read.SAScii(fn = here("Study 2/raw data/HRS/H16C_R.da"),
                         sas_ri = here("Study 2/raw data/HRS/H16C_R.sas"))

#select variables in main data set

hrs <- hrs %>%
  dplyr::select(id = HHIDPN,
         birthyear = RABYEAR,
         gender = RAGENDER,
         race = RARACEM,
         edu = RAEDYRS,
         srh_06 = R8SHLT,
         srh_08 = R9SHLT,
         srh_10 = R10SHLT,
         srh_12 = R11SHLT,
         srh_14 = R12SHLT,
         diabetes_06 = R8DIABE,
         diabetes_08 = R9DIABE,
         diabetes_10 = R10DIABE,
         diabetes_12 = R11DIABE,
         diabetes_14 = R12DIABE,
         smoker_06 = R8SMOKEN,
         smoker_08 = R9SMOKEN,
         smoker_10 = R10SMOKEN,
         smoker_12 = R11SMOKEN,
         smoker_14 = R12SMOKEN,
         heart_06 = R8HEARTE,
         heart_08 = R9HEARTE,
         heart_10 = R10HEARTE,
         heart_12 = R11HEARTE,
         heart_14 = R12HEARTE)

# select variables in yearly data sets,
# filter out participants without data,
# rename variables

h06data <- h06data %>%
  dplyr::select(
    id = HHIDPN, # id
         purpose1_06 = KLB035A,
         purpose2_06 = KLB035B,
         purpose3_06 = KLB035C,
         purpose4_06 = KLB035D,
         purpose5_06 = KLB035E,
         purpose6_06 = KLB035F,
         purpose7_06 = KLB035G)

names(h08data) <- toupper(names(h08data))

h08data <- h08data %>%
  dplyr::select(
    id = HHIDPN, # id
         purpose1_08 = LLB035A,
         purpose2_08 = LLB035B,
         purpose3_08 = LLB035C,
         purpose4_08 = LLB035D,
         purpose5_08 = LLB035E,
         purpose6_08 = LLB035F,
         purpose7_08 = LLB035G)


h10data <- h10data %>%
  dplyr::select(id = HHIDPN, # id
         purpose1_10 = MLB035A,
         purpose2_10 = MLB035B,
         purpose3_10 = MLB035C,
         purpose4_10 = MLB035D,
         purpose5_10 = MLB035E,
```

```
         purpose6_10 = MLB035F,
         purpose7_10 = MLB035G)

h12data <- h12data %>%
  dplyr::select(id = HHIDPN,
         purpose1_12 = NLB035A,
         purpose2_12 = NLB035B,
         purpose3_12 = NLB035C,
         purpose4_12 = NLB035D,
         purpose5_12 = NLB035E,
         purpose6_12 = NLB035F,
         purpose7_12 = NLB035G)

h14data <- h14data %>%
  dplyr::select(id = HHIDPN,
                purpose1_14 = OLB033A,
         purpose2_14 = OLB033B,
         purpose3_14 = OLB033C,
         purpose4_14 = OLB033D,
         purpose5_14 = OLB033E,
         purpose6_14 = OLB033F,
         purpose7_14 = OLB033G)


h16data_lb = h16data_lb %>%
  dplyr::select(HHID = HHID,
         PN = PN,
         purpose1_16 = PLB033A,
         purpose2_16 = PLB033B,
         purpose3_16 = PLB033C,
         purpose4_16 = PLB033D,
         purpose5_16 = PLB033E,
         purpose6_16 = PLB033F,
         purpose7_16 = PLB033G) %>%
  mutate(id = as.numeric(HHID)*1000 + as.numeric(PN)) %>%
  dplyr::select(-HHID, -PN)

h16data_c = h16data_c %>%
  dplyr::select(HHID = HHID,
         PN = PN,
         diabetes_16 = PC010,
         srh_16 = PC001,
         smoker_16 = PC117,
         heart_16 = PC036) %>%
  mutate(id = as.numeric(HHID)*1000 + as.numeric(PN)) %>%
  dplyr::select(-HHID, -PN)

h16data_c = scrub(h16data_c,
                  where = c("diabetes_16", "srh_16", "smoker_16", "heart_16"),
                  isvalue = 8)

h16data_c = scrub(h16data_c,
                  where = c("diabetes_16", "srh_16", "smoker_16", "heart_16"),
                  isvalue = 9)

h16data_c$diabetes_16 = ifelse(h16data_c$diabetes_16 %in% c(1,3), 1, 0)
h16data_c$heart_16 = ifelse(h16data_c$heart_16 %in% c(1,3), 1, 0)
h16data_c$smoker_16 = ifelse(h16data_c$smoker_16 == 1, 1, 0)

# merge data sets,
hrs <- hrs %>%
  left_join(h06data) %>%
  left_join(h08data) %>%
  left_join(h10data) %>%
  left_join(h12data) %>%
  left_join(h14data) %>%
  left_join(h16data_lb) %>%
  left_join(h16data_c)

hrs = hrs %>%

  # gather into long form, with each purpose response for each
  # participant at each year having its own row
  gather(key="variable",value="value",
         which(grepl("purpose", names(.)))) %>%

  #remove rows with missing purpose values
```

```
  filter(!is.na(value)) %>%

  #separate the purpose variable into two columns, one with the variable and one with the year
  separate(col = "variable", into = c("variable","pyear")) %>%

  # spread into a different long form, with each year for each participant having its own row
  spread(key="variable", value="value") %>%

  # group by participant, and then for each person,
  # dplyr::select only the row with the smallest (first) year
  group_by(HHIDPN) %>%
  filter(pyear == min(pyear)) %>%
  ungroup() %>%

  # gather the remaining repeated values (i.e., have an underscore) into a long form with
  # each response on each variable for each participant at each year having its own row
  gather(key="variable",value="value", which(grepl("_",names(.))), convert=T) %>%

  #remove missing values
  filter(!is.na(value)) %>%

  # separate the variable column into two, one with the variable and one with the year
  separate(col = "variable", into = c("variable","year")) %>%

  # spread back into long form with each year for each participant having its own row
  # note: purpose responses from the first year will be repeated across rows
  spread(key="variable", value="value") %>%

  #identify the last year of assessment for each participant
  group_by(HHIDPN) %>%
  mutate(last_year = max(as.numeric(year)),
         year = as.numeric(year),
         time = year-last_year,
         pyear = as.numeric(pyear)) %>%
  # remove years before purpose in life assessment
  filter(year >= pyear) %>%
  # fix variables to be consistent with other studies
  mutate(age = as.numeric(year)+2000 - birthyear,
         gender = gender - 1,
         race = ifelse(race == 1, 0, 1),
         srh = srh*-1 + 6,
         b.age = as.numeric(pyear)+2000 - birthyear)

save(hrs, file=here("Study 2/created data/hrs.Rdata"))
```

1.5 KGSS

```
#load raw data
load(here("Study 2/raw data/KGSS/34665-0001-Data.rda"))
load(here("Study 2/raw data/KGSS/34666-0001-Data.rda"))
load(here("Study 2/raw data/KGSS/35334-0001-Data.rda"))
load(here("Study 2/raw data/KGSS/35335-0001-Data.rda"))
kgss_09 = da34665.0001; rm(da34665.0001)
kgss_10 = da34666.0001; rm(da34666.0001)
kgss_11 = da35334.0001; rm(da35334.0001)
kgss_12 = da35335.0001; rm(da35335.0001)

kgss_09$year = 2009
kgss_10$year = 2010
kgss_11$year = 2011
kgss_12$year = 2012

#select variables in main data set

kgss_09 <- kgss_09 %>%
  dplyr::select(
         id = RESPID,
         b.age = AGE,
         gender = SEX,
         edu = EDUC,
         purpose1 = DOMOREWK,
         purpose2 = RSNTOLIV,
         purpose3 = HAVEPLAN,
```

```
         purpose4 = LIFCNTRL,
         srh_09 = HEALTH,
         diabetes = DIABETES,
         heart_09 = HEARTPRB,
         year_09 = year) %>%
  #make numeric
  mutate(gender = as.numeric(gender),
         edu= as.numeric(edu),
         purpose1= as.numeric(purpose1),
         purpose2= as.numeric(purpose2),
         purpose3= as.numeric(purpose3),
         purpose4= as.numeric(purpose4),
         srh_09 = as.numeric(srh_09),
         diabetes= as.numeric(diabetes),
         heart_09 = as.numeric(heart_09)) %>%
  # make binary varibales 0, 1
  mutate(gender = gender - 1,
         purpose1 = ifelse(purpose1 == 1, 1, 0),
         purpose2 = ifelse(purpose2 == 1, 1, 0),
         purpose3 = ifelse(purpose3 == 1, 1, 0),
         purpose4 = ifelse(purpose4 == 1, 1, 0),
         diabetes = ifelse(diabetes == 1, 1, 0),
         heart_09 = ifelse(heart_09 == 1, 1, 0),
         #reverse score srh
         srh_09 = srh_09*-1 + 6,
         # remove "other" from education
         edu = ifelse(edu < 9, edu, NA))

kgss_10 = kgss_10 %>%
  dplyr::select(
         id = RESPID,
         srh_10 = HEALTHY,
         smoker_10 = FREQSMOK,
         heart_10 = HEARTP10,
         year_10 = year) %>%
  mutate(smoker_10 = as.numeric(smoker_10),
         smoker_10 = ifelse(smoker_10 == 5, 0, 1),
         heart_10 = as.numeric(heart_10),
         heart_10 = ifelse(heart_10 == 1, 1, 0),
         srh_10 = as.numeric(srh_10)*-1 + 6)

kgss_11 = kgss_11 %>%
  dplyr::select(
         id = RESPID,
         srh_11 = HEALTHY,
         smoker_11 = FREQSM10,
         year_11 = year) %>%
  mutate(smoker_11 = as.numeric(smoker_11),
         smoker_11 = ifelse(smoker_11 %in% c(1,2), 0, 1),
         srh_11 = as.numeric(srh_11)*-1 + 6)

kgss_12 = kgss_12 %>%
  dplyr::select(
         id = RESPID,
         srh_12 = HEALTHY,
         year_12 = year) %>%
  mutate(srh_12 = as.numeric(srh_12)*-1 + 6)

kgss = full_join(kgss_09, kgss_10) %>%
  full_join(kgss_11) %>%
  full_join(kgss_12) %>%
  gather(key = "key", value = "value", which(grepl("_", names(.)))) %>%
  separate("key", c("key","wave"), sep ="_") %>%
  spread("key", "value") %>%
  mutate(time = year - 2012) %>%
  group_by(id) %>%
  mutate(time = time - max(time)) %>%
  filter(!is.na(year))

## due to convergence issues, we use only heart disease data from individuals with data at both time points

# one.heart = kgss %>%
#   group_by(id) %>%
#   filter(!is.na(heart)) %>%
#   summarize(n = n()) %>%
#   filter(n == 1)
#
```

```
# kgss£heart[kgss£id %in% one.heart£id] = NA

# # due to multicollinearity, we remove education from the models
#
# kgss = kgss %>% dplyr::select(-edu)

# due to low response rates regarding heart disease, we remove this outcome from the kgss analyses
table(kgss$heart, kgss$diabetes)

kgss = kgss %>% dplyr::select(-heart)

save(kgss, file=here("Study 2/created data/kgss.Rdata"))
```

1.6 MIDJA

```
#load raw data
load(here("Study 2/raw data/MIDJA/30822-0001-Data.rda"))
load(here("Study 2/raw data/MIDJA/36427-0001-Data.rda"))
midja_08 = da30822.0001; rm(da30822.0001)
midja_12 = da36427.0001; rm(da36427.0001)

midja = full_join(midja_08, midja_12)

#dplyr::select variables in main data set

midja <- midja %>%
  dplyr::select(
         id = MIDJA_IDS,  # id
         b.age = J1SQ2AGE,
         gender = J1SQ1,
         edu = J1SQ3,
         purpose1 = J1SJ8E, # purpose in life
         purpose2 = J1SJ8K, # purpose in life
         purpose3 = J1SJ8Q, # purpose in life
         purpose4 = J1SJ8W, # purpose in life
         purpose5 = J1SJ8CC, # purpose in life
         purpose6 = J1SJ8NN, # purpose in life
         purpose7 = J1SJ8PP, # purpose in life
         diabetes = J1SA8X,
         srh_0 = J1SA1,
         eversmoker_0 = J1SB1A,
         smoker_0 = J1SB4,
         heart_0 = J1SA9D,
         srh_4 = K1SA1,
         smoker_4 = K1SB1,
         heart_4 = K1SA10D) %>%
  #make values numeric, not factor
  mutate(gender = as.numeric(gender),
         edu = as.numeric(edu),
         purpose1 = as.numeric(purpose1),
         purpose2 = as.numeric(purpose2),
         purpose3 = as.numeric(purpose3),
         purpose4 = as.numeric(purpose4),
         purpose5 = as.numeric(purpose5),
         purpose6 = as.numeric(purpose6),
         purpose7 = as.numeric(purpose7),
         diabetes = as.numeric(diabetes),
         srh_0 = as.numeric(srh_0),
         eversmoker_0 = as.numeric(eversmoker_0),
         smoker_0 = as.numeric(smoker_0),
         heart_0 = as.numeric(heart_0),
         srh_4 = as.numeric(srh_4),
         smoker_4 = as.numeric(smoker_4),
         heart_4 = as.numeric(heart_4)) %>%
  gather(key = "variable", value = 'value', which(grepl("_", names(.)))) %>%
  separate(col = "variable", into = c("variable", "time"), sep = "_") %>%
  spread(key = "variable", value = "value")
#

#remove missing values
midja = scrub(midja, where = "eversmoker", isvalue = 3)

#make binary (0,1)
```

```
midja = midja %>%
  mutate(gender = gender - 1,
         diabetes = ifelse(diabetes == 1, 1, 0),
         eversmoker = ifelse(eversmoker == 1, 1, 0),
         smoker = ifelse(smoker == 1, 1, 0),
         heart = ifelse(heart == 1, 1, 0),
         time = as.numeric(time)) %>%
  group_by(id) %>%
  mutate(time = time - max(time))

midja$smoker[midja$eversmoker == 0 & is.na(midja$smoker)] = 0


save(midja, file=here("Study 2/created data/midja.Rdata"))
```

1.7 MIDUS

```
midus1 <- read_sav(here("Study 2/raw data/MIDUS/02760-0001-Data.sav"))
midus2 <- read_sav(here("Study 2/raw data/MIDUS/04652-0001-Data.sav"))
midus3 <- read_sav(here("Study 2/raw data/MIDUS/36346-0001-Data.sav"))

midus <- full_join(midus1, midus2) %>%
  full_join(midus3) %>%
  dplyr::select(
         id = M2ID,
         purpose1 = A1SF1C,
         purpose2 = A1SF1G,
         purpose3 = A1SF1J,
         diabetes = A1SA9X,
         srh_0 = A1PA4,
         cig.age_0 = A1PA41,
         smoke.ever_0 = A1PA40,
         smoker_0 = A1PA43,
         heart_0 = A1PA29,
         heart.highbp_0 = A1PA29CC,
         heart.none_0 = A1PA29CK,
         srh_9 = B1PA1,
         cig.age_9 = B1PA37,
         smoke.ever_9 = B1PA38A,
         smoker_9 = B1PA39,
         heart_9 = B1PA7,
         heart.highbp_9 = B1PA7BC,
         srh_18 = C1PA1,
         cig.age_18 = C1PA37,
         smoke.ever_18 = C1PA38A,
         smoker_18 = C1PA39,
         heart_18 = C1PA7,
         heart.highbp_18 = C1PA7BC,
         gender = A1PRSEX,
         b.age = A1PAGE_M2,
         race = A1SS7,
         edu = A1PB1) %>%
  mutate(srh_9 = srh_9*-1+6,
         srh_18 = srh_18*-1+6) %>%
  gather(key = "key", value = "value", which(grepl("_", names(.)))) %>%
  separate("key", into = c("key", "time"), sep = "_") %>%
  spread("key", "value") %>%
  mutate(time = as.numeric(time)) %>%
  group_by(id) %>%
  mutate(time = time - max(time))


#remove values that mean missing or don't know
midus <- scrub(midus, where=c("srh", "smoker",
                              "heart.highbp","heart.none"),
               isvalue = 7)

midus <- scrub(midus, where=c("purpose1","purpose2","purpose3",
                              "diabetes", "race", "gender"),
               isvalue = 8)

#reverse code purpose items
purpose.items <- midus[,which(grepl("purpose",names(midus)))]
```

```
purpose.items <- reverse.code(keys = rep(-1, ncol(purpose.items)),
                              items = purpose.items)
midus[,which(grepl("purpose",names(midus)))] <- purpose.items


midus <- scrub(midus, where="edu",
               isvalue = 97)

#recode values that mean "no" in binary questions to 0
midus <- scrub(midus, where=c("diabetes", "smoker", "heart",
                              "heart.highbp","heart.none"),
               isvalue = 2, newvalue = 0)

#use values from prior questions to fill in responses. E.g., if answered "never had a cig," then not a current smoker
midus$smoker[midus$cig.age == 96] <- 0
midus$smoker[midus$smoke.ever == 2] <- 0


#gender binary with 0 = male
midus$gender = midus$gender-1
#race binary with 0 = white
midus$race = ifelse(midus$race == 1, 0, 1)

#heart condition variable (don't include hypertension)

midus$heart[midus$heart.highbp == 1] = 0
midus$heart[midus$heart.none == 1] = 0

midus = dplyr::select(midus, -cig.age, -smoke.ever, -heart.highbp, -heart.none)

midus$diabetes = as.numeric(midus$diabetes)

save(midus, file=here("Study 2/created data/midus.Rdata"))
```

1.8 SWAN

```
#load raw data
swan_0 = read_sav(here("Study 2/raw data/SWAN/04368-0001-Data.sav"))
swan_1 = read_sav(here("Study 2/raw data/SWAN/29221-0001-Data.sav"))
swan_2 = read_sav(here("Study 2/raw data/SWAN/29401-0001-Data.sav"))
swan_3 = read_sav(here("Study 2/raw data/SWAN/29701-0001-Data.sav"))
swan_4 = read_sav(here("Study 2/raw data/SWAN/30142-0001-Data.sav"))
swan_5 = read_sav(here("Study 2/raw data/SWAN/30501-0001-Data.sav"))
swan_6 = read_sav(here("Study 2/raw data/SWAN/31181-0001-Data.sav"))
swan_7 = read_sav(here("Study 2/raw data/SWAN/31901-0001-Data.sav"))
swan_8 = read_sav(here("Study 2/raw data/SWAN/32122-0001-Data.sav"))
swan_9 = read_sav(here("Study 2/raw data/SWAN/32721-0001-Data.sav"))
swan_10 = read_sav(here("Study 2/raw data/SWAN/32961-0001-Data.sav"))

swan_0$SWANID = as.numeric(swan_0$ID)
swan_0$SMOKER = swan_0$SMOKER+1
swan_1 = dplyr::select(swan_1, -VISIT)
swan_2 = dplyr::select(swan_2, -VISIT)
swan_3 = dplyr::select(swan_3, -VISIT)
swan_4 = dplyr::select(swan_4, -VISIT)
swan_5 = dplyr::select(swan_5, -VISIT)
swan_6 = dplyr::select(swan_6, -VISIT)
swan_7 = dplyr::select(swan_7, -VISIT)
swan_8 = dplyr::select(swan_8, -VISIT)
swan_9 = dplyr::select(swan_9, -VISIT)
swan_10 = dplyr::select(swan_10, -VISIT)

#select variables in main data set
swan <- full_join(swan_0, swan_1) %>%
  full_join(swan_2) %>%
  full_join(swan_3) %>%
  full_join(swan_4) %>%
  full_join(swan_5) %>%
  full_join(swan_6) %>%
  full_join(swan_7) %>%
  full_join(swan_8) %>%
  full_join(swan_9) %>%
  full_join(swan_10) %>%
```

```
  dplyr::select(id = ID,  # id
         b.age = AGE,
         edu = DEGREE,
         race = ETHNIC,
         purpose1 = MISSION, # purpose in life
         purpose2 = FAITH, # purpose in life
         purpose3 = DIFFICU, # purpose in life
         diabetes = DIABETE,
         srh_0 = HEALTH,
         smoker_0 = SMOKER,
         heart_0 = HEART,
         srh_1 = OVERHLT1,
         smoker_1 = SMOKERE1,
         heartat_1 = HEARTAT1,
         angina_1 = ANGINA1,
         srh_2 = OVERHLT2,
         smoker_2 = SMOKERE2,
         heartat_2 = HEARTAT2,
         angina_2 = ANGINA2,
         srh_3 = OVERHLT3,
         smoker_3 = SMOKERE3,
         heartat_3 = HEARTAT3,
         angina_3 = ANGINA3,
         srh_4 = OVERHLT4,
         smoker_4 = SMOKERE4,
         heartat_4 = HEARTAT4,
         angina_4 = ANGINA4,
         srh_5 = OVERHLT5,
         smoker_5 = SMOKERE5,
         heartat_5 = HEARTAT5,
         angina_5 = ANGINA5,
         srh_6 = OVERHLT6,
         smoker_6 = SMOKERE6,
         heartat_6 = HEARTAT6,
         angina_6 = ANGINA6,
         srh_7 = OVERHLT7,
         smoker_7 = SMOKERE7,
         heartat_7 = HEARTAT7,
         angina_7 = ANGINA7,
         srh_8 = OVERHLT8,
         smoker_8 = SMOKERE8,
         heartat_8 = HEARTAT8,
         angina_8 = ANGINA8,
         srh_9 = OVERHLT9,
         smoker_9 = SMOKERE9,
         heartat_9 = HEARTAT9,
         angina_9 = ANGINA9,
         srh_10 = OVERHLT10,
         smoker_10 = SMOKERE10,
         heartat_10 = HEARTAT10,
         angina_10 = ANGINA10) %>%
  filter(!is.na(id))

swan[,grepl("_", names(swan))] = apply(swan[,grepl("_", names(swan))],
                                       MARGIN = 2, as.numeric)

swan = swan %>%
  gather(key = "variable", value = "value",
         which(grepl("_", names(.)))) %>%
  separate("variable", into = c("variable", "time"), sep = "_") %>%
  spread(key = "variable", value = "value") %>%
  mutate(time = as.numeric(time)) %>%
  group_by(id) %>%
  mutate(time = as.numeric(time))

#remove missing values
swan = scrub(swan, min = 0)

swan$heart[swan$heartat == 1 | swan$angina == 1] = 1
swan$heart[swan$heartat == 2 | swan$angina == 2] = 2


swan = swan %>%
  mutate(race = ifelse(race == 10, 0, 1),  #make race binary (1 = nonwhite, 0 = white)
         diabetes = ifelse(diabetes == 2, 1, 0), #make diabetes binary (1 = yes, 0 = no)
         srh = srh*-1 + 6, # reverse code self-rated health
         edu = as.numeric(edu),
```

```
        time = time - max(time, na.rm=T),
        purpose1 = purpose1*-1 + 4,
        purpose2 = purpose2*-1 + 4,
        purpose3 = purpose3*-1 + 4,
        heart = ifelse(heart == 2, 1, 0),  # make heart binary
        smoker = smoker - 1)

save(swan, file=here("Study 2/created data/swan.Rdata"))
```

1.9 WLS

```
wls <- read_sas(here("Study 2/raw data/WLS/wls_b_13_06.sas7bdat"))

wls_grd <- wls %>%
  dplyr::select(
        id = idpub,
        purpose1 = MN039RER,
        purpose2 = MN040RER,
        purpose3 = MN041RER,
        purpose4 = MN042RER,
        purpose5 = MN043RER,
        purpose6 = MN044RER,
        purpose7 = MN045RER,
        diabetes = MX095RER,
        srh_0 = MX001RER,
        heart_0 = MX099RER,
        srh_11 = IX001RER,
        heart_11 = GX351RE,
        srh_19 = jx001rer,
        heart_19 = hx351re,
        gender = sexrsp,
        b.age = RA029RE,
        edu = RB003RED) %>%
  gather(key="variable", value="value", which(grepl("_", names(.)))) %>%
  separate(col = "variable", into=c("variable","time")) %>%
  spread(key="variable", value="value") %>%
  mutate(time = as.numeric(time),
        cohort = "grd")


wls_sib <- wls %>%
  dplyr::select(
    id = idpub,
        purpose1 = NP039RER,
        purpose2 = NP040RER,
        purpose3 = NP041RER,
        purpose4 = NP042RER,
        purpose5 = NP043RER,
        purpose6 = NP044RER,
        purpose7 = NP045RER,
        diabetes = NX115RER,
        srh_0 = NX001RER,
        heart_0 = NX119RER,
        smokerever_0 = NX038RER,
        smokernow_0 = NX039REC,
        srh_12 = DX001RER,
        heart_12 = CX351RE,
        smokerever_12 = CX015RER,
        smokernow_12 = CX016RER,
        srh_18 = px001rer,
        heart_18 = kx351re,
        gender = ssbsex,
        b.age = SA029RE,
        edu = SB003RED) %>%
  gather(key="variable", value="value", which(grepl("_", names(.)))) %>%
  separate(col = "variable", into=c("variable","time")) %>%
  spread(key="variable", value="value") %>%
  mutate(time = as.numeric(time),
        cohort = "sib")

wls = full_join(wls_grd, wls_sib) %>%
  unite(id, id, cohort, sep = "_")
```

```
#remove values that mean missing or don't know
wls <- scrub(wls, min = 0)

#recode values that mean "no" in binary questions to 0
wls <- scrub(wls, where=c("diabetes", "smokerever", "smokernow", "heart"),
               isvalue = 2, newvalue = 0)

#reverse code purpose items
purpose.items <- wls[,which(grepl("purpose",names(wls)))]
purpose.items <- reverse.code(keys = rep(-1, ncol(purpose.items)),
                              items = purpose.items)
wls[,which(grepl("purpose",names(wls)))] <- purpose.items



#smoking binary with 0 = not a current smoker
wls$smoker <- ifelse(wls$smokerever == 0, 0, wls$smokernow)

#gender binary with 0 = male
wls$gender = wls$gender-1

wls = wls %>%
  group_by(id) %>%
  mutate(time = time - max(time, na.rm=T))

save(wls, file=here("Study 2/created data/wls.Rdata"))
```

2 Analyze individual studies

2.1 Code for all studies

A single script file was written that could be sourced with each of the datasets in turn. The script was written in order to allow us to loop through each of the data sets in turn.

First, assume i refers to the name of a dataset. The cleaned data is loaded into R.

```
load(here(paste0("Study 2/created data/", i, ".Rdata")))

dataset = get(i)

#filter out nodiabetes
dataset = dataset %>% as_tibble() %>%  dplyr::filter(diabetes == 1)
```

Next, the purpose scale is scored and the alpha coefficient is extracted.

```
# how many purpose items
purpose.items = length(names(dataset)[grepl("purpose", names(dataset))])


# harmonize scaling. All purpose items will be score on a scale from 0 to 5
min.p = min(dataset[,grepl("purpose", names(dataset))], na.rm=T)
max.p = max(dataset[,grepl("purpose", names(dataset))], na.rm=T)

if (purpose.items > 1){

  purpose.alpha = dataset[grepl("purpose", names(dataset))] %>%
    mutate(across(everything(), as.numeric)) %>%
    psych::alpha(check.keys = TRUE)

  keys = purpose.alpha$keys

  if (keys[1] != first.key){keys = keys*-1}

  # score dataset
  dataset$purpose = dataset[grepl("purpose", names(dataset))] %>%
    reverse.code(keys = keys, items = .) %>%
    rowMeans(na.rm=T)

} else (purpose.alpha = NULL)
```

We record the total sample size of the study, then filter out participants who do not meet elligability criteria. Participants must have a measure of purpose, must have information regarding their diabetes status, must have information on at leeast one of the outcomes measures and must have all covariates. We record the new sample size.

```
#identify which columns are in data set

# possible outcome columns
outcomes <- names(dataset)[which(names(dataset) %in% c("srh", "smoker", "heart"))]

# possible covariate columns
covariates <- names(dataset)[which(names(dataset) %in% c("b.age", "gender", "race", "edu"))]

# filter out participants without purpose, without at least one outcome
dataset = filter(dataset, !is.na(purpose))

if (length(outcomes) > 1){
  missing.outcomes = apply(X = dataset[,outcomes], MARGIN = 1, FUN = function(x) sum(is.na(x)))
  dataset = dataset[missing.outcomes < length(outcomes), ]
} else{
  dataset = filter(dataset, !is.na(outcomes))
}

missing.covariates = apply(X = dataset[,covariates], MARGIN = 1, FUN = function(x) sum(is.na(x)))

dataset = dataset[missing.covariates == 0, ]
analysisSampleP = length(unique(dataset$id))
analysisSampleObs = nrow(dataset)

maxwaves = max(abs(dataset$time), na.rm=T)
```

Additional datasets are created to represent only participants with diabetes and only nonparticipants with diabetes. Continuous measures are standardized within all three datasets.

Descriptive statistics and correlations are calculated for the sample. A correlation table is saved to a word document for reference.

```
dataset %>%
  dplyr::select(time, purpose, outcomes, covariates) %>%
  apa.cor.table(filename = here(paste0("Study 2/tables and figures/tables/",i,"_descriptives.doc")))

descriptives <- dataset %>%
  dplyr::select(time, purpose, outcomes, covariates) %>%
  describe(fast=T)


if ("smoker" %in% outcomes) {

  smoker.tab <- dataset %>%
    dplyr::select(smoker, time) %>%
    table()

} else {smoker.tab = NULL}

if ("heart" %in% outcomes){

  heart.tab <- dataset %>%
    dplyr::select(heart, time) %>%
    table()

} else {heart.tab = NULL}

# recalculate alpha on smaller sample

if (purpose.items > 1){
  purpose.alpha = dataset[grepl("purpose", names(dataset))] %>%
    mutate(across(everything(), as.numeric)) %>%
    psych::alpha(check.keys = TRUE)
  }
```

We calculate the correlation between purpose and self-rated health. We do this separately for the participants with and without diabetes.

We calculate estimate the difference in purpose scores between smokers and nonsmokers; we do this separately for the participants with and without diabetes.

We calculate estimate the difference in purpose scores between participants with heart disease and participants without heart disease; we do this separately for the participants with and without diabetes.

Regression formulas are created, based on which covariates are available in that sample.

```
avg.slope <- paste0("~ time + ",
                    paste(covariates, collapse = " + "),
                    " + (1 + time | id)")
avg.slope = gsub("b.age", "c.age", avg.slope)

purpose.slope <- paste0("~ time*purpose + ",
                    paste(covariates, collapse = " + "),
                    " + (1 + time | id)")
purpose.slope = gsub("b.age", "c.age", purpose.slope)

lowpurpose.slope <- paste0("~ time*low.purpose + ",
                        paste(covariates, collapse = " + "),
                        " + (1 + time | id)")
lowpurpose.slope = gsub("b.age", "c.age", lowpurpose.slope)

hghpurpose.slope <- paste0("~ time*hgh.purpose + ",
                           paste(covariates, collapse = " + "),
                           " + (1 + time | id)")
hghpurpose.slope = gsub("b.age", "c.age", hghpurpose.slope)




#LMER Control Settings
nlopt <- function(par, fn, lower, upper, control) {
  .nloptr <<- res <- nloptr(par, fn, lb = lower, ub = upper,
                            opts = list(algorithm = "NLOPT_LN_BOBYQA", print_level = 1,
                                        maxeval = 1000, xtol_abs = 1e-6, ftol_abs = 1e-6))
  list(par = res$solution,
       fval = res$objective,
       conv = if (res$status > 0) 0 else res$status,
       message = res$message)
}
```

We analyze the interaction of purpose and diabetes on each of the outcomes available, controlling for the covariates available. Continuous measures are standardized. Logistic regression is used for binary outcomes.

We extract predicted values for these models.

```
if ("srh" %in% outcomes) {
  plot.srh.full = plot_model(mod.srh.purpose, type = "pred", ci.lvl = NA,
                      terms = c("time", "purpose"))
  plot.srh.plot = plot_model(mod.srh.purpose, type = "pred", ci.lvl = NA,
                           terms = c("time", "purpose [-1,1]"))
} else {plot.srh.full = NULL; plot.srh.plot = NULL}
if ("smoker" %in% outcomes) {
  plot.smoker.full = plot_model(mod.smoker.purpose, type = "pred", ci.lvl = NA,
                              terms = c("time", "purpose"))
  plot.smoker.plot = plot_model(mod.smoker.purpose, type = "pred", ci.lvl = NA,
                              terms = c("time", "purpose [-1,1]"))
} else {plot.smoker.full = NULL; plot.smoker.plot = NULL}
if ("heart" %in% outcomes) {
  plot.heart.full = plot_model(mod.heart.purpose, type = "pred", ci.lvl = NA,
                        terms = c("time", "purpose"))
  plot.heart.plot = plot_model(mod.heart.purpose, type = "pred", ci.lvl = NA,
                             terms = c("time", "purpose [-1,1]"))
} else {plot.heart.full = NULL; plot.heart.plot = NULL}
```

We analyze the relationship between purpose and each ouf the available outcomes, controlling for the covariates available. Continuous measures are standardized. Logistic regression is used for binary outcomes. We do this only for adults with diabetes.

We repeat the last set of regressions in the sample of only adults without diabetes.

Finally, we gather the analysis results into a single list and save this as a new R object. This object is saved to an external file for later use.

```
return.object = list(
  descriptives = list(
    totalP = analysisSampleP,
    studyObs = analysisSampleObs,
    alpha = purpose.alpha,
    describe = descriptives,
    smoker.tab = smoker.tab,
    heart.tab = heart.tab),
  regression = list(
    purpose = list(
      srh = mod.srh.purpose,
      smoker = mod.smoker.purpose,
      heart = mod.heart.purpose),
    average = list(
      srh = mod.srh.avg,
      smoker = mod.smoker.avg,
      heart = mod.heart.avg),
    low.purpose = list(
      srh = mod.srh.low,
      smoker = mod.smoker.low,
      heart = mod.heart.low),
    hgh.purpose = list(
      srh = mod.srh.hgh,
      smoker = mod.smoker.hgh,
      heart = mod.heart.hgh)),
  plotdata = list(
    fulldata = list(
      srh = plot.srh.full$data,
      smoker = plot.smoker.full$data,
      heart = plot.heart.full$data),
    forplot = list(
      srh = plot.srh.plot$data,
      smoker = plot.smoker.plot$data,
      heart = plot.heart.plot$data)
))

assign(x = paste(i, "out", sep="_"), return.object)
save(list = paste(i, "out", sep="_"),
     file = here(paste0("Study 2/created data/", i, "_out.Rdata")))
```

2.2 Source script on all studies

While running the script, we also extract the number of purpose items and the mean age of the sample, which will be used in the moderation analyses when trying to explain heterogeneity between the studies.

```
set.seed(080919)

study.names = c("acl", "alsa", "elsa",
                "hrs", "kgss", "midja",
                "midus", "swan", "wls")
```

```
keys.df = data.frame(study = study.names,
                     first.key = c(1, 1, 1,
                                   1, 1,-1,
                                   1, 1, 1))

study.level = data.frame(study = study.names)

for(i in study.names){
  first.key = keys.df$first.key[keys.df$study == i]
  source(here("Study 2/scripts/study_analysis.R"))

  study.level$number_items[study.level$study == i] = purpose.items
  study.level$age[study.level$study == i] = descriptives["b.age", "mean"]
  study.level$maxwaves[study.level$study == i] = maxwaves
  source(here("Study 2/scripts/study_analysis_quad.R"))

  rm(list = setdiff(ls(), c("path", "study.names", "study.level", "keys.df",
                            "num", "def.chunk.hook")))
}
save(study.level, file = here("Study 2/created data/study_level.Rdata"))
```

3 Descriptive Statistics

3.1 Mean, standard deviation, min, max, alpha

```
# pull the describe data.frame out of each study list
describe.df = lapply(X = study.names,
                     FUN = function(x) get(paste0(x,"_out"))$descriptives$describe) %>%
  # add to each a column called study and a column of variable names
  map2_df(., study.names, ~ mutate(.x, study = .y, var = rownames(.x))) %>%
  # dplyr::select the columns we want to include in the table
  dplyr::select(study, var, n, mean, sd, min, max)

# grab alpha for purpose
alpha.df = data.frame(study = study.names[-1],
                      var = "purpose")
alpha.df$alpha = unlist(lapply(X = study.names,
                     FUN = function(x) get(paste0(x,"_out"))$descriptives$alpha$total$raw_alpha))

describe.df = describe.df %>%
  full_join(alpha.df)

# which rows belong to which study?

acl.rows = which(describe.df$study == "acl")
alsa.rows = which(describe.df$study == "alsa")
elsa.rows = which(describe.df$study == "elsa")
hrs.rows = which(describe.df$study == "hrs")
kgss.rows = which(describe.df$study == "kgss")
midja.rows = which(describe.df$study == "midja")
midus.rows = which(describe.df$study == "midus")
swan.rows = which(describe.df$study == "swan")
wls.rows = which(describe.df$study == "wls")

# build table
describe.df %>%
  dplyr::select(-study) %>%
  kable(.,  caption = "Descriptives Table",
        booktabs = T, escape = F, digits = 2, format = "latex", longtable = T,
        col.names = c("Variable", "N Valid", "Mean", "SD", "Min", "Max", "alpha")) %>%
  kable_styling(latex_options = c("repeat_header", "hold_position")) %>%
  column_spec(1, width = "5cm") %>%
  group_rows("ACL", min(acl.rows), max(acl.rows)) %>%
  group_rows("ALSA", min(alsa.rows), max(alsa.rows)) %>%
  group_rows("ELSA", min(elsa.rows), max(elsa.rows)) %>%
  group_rows("HRS", min(hrs.rows), max(hrs.rows)) %>%
  group_rows("KGSS", min(kgss.rows), max(kgss.rows)) %>%
  group_rows("MIDJA", min(midja.rows), max(midja.rows)) %>%
  group_rows("MIDUS", min(midus.rows), max(midus.rows)) %>%
  group_rows("SWAN", min(swan.rows), max(swan.rows)) %>%
  group_rows("WLS", min(wls.rows), max(wls.rows))
```

Table 1: Descriptives Table

Variable	N Valid	Mean	SD	Min	Max	alpha
**ACL**						
time	264	-4.99	5.02	-11.00	0.00	
purpose	264	4.30	1.08	1.00	5.00	
heart	255	0.09	0.28	0.00	1.00	
smoker	204	0.14	0.35	0.00	1.00	
srh	264	2.83	1.05	1.00	5.00	
gender	264	0.61	0.49	0.00	1.00	
b.age	264	58.68	9.45	40.00	84.00	
race	264	0.39	0.49	0.00	1.00	
edu	264	12.39	2.40	8.00	16.00	
**ALSA**						
time	435	-1.00	0.82	-2.00	0.00	
purpose	435	3.33	0.90	1.00	5.00	0.88
srh	350	2.71	1.05	1.00	5.00	
gender	435	0.44	0.50	0.00	1.00	
edu	435	9.60	3.24	2.00	20.00	
b.age	435	79.16	6.15	68.00	100.00	
**ELSA**						
time	112	-3.79	2.75	-8.00	0.00	

Table 1: Descriptives Table *(continued)*

Variable	N Valid	Mean	SD	Min	Max	alpha
purpose	122	3.24	0.58	2.24	4.52	0.74
heart	122	0.36	0.48	0.00	1.00	
smoker	122	0.16	0.36	0.00	1.00	
srh	91	2.63	0.98	1.00	5.00	
gender	122	0.49	0.50	0.00	1.00	
b.age	122	68.82	9.66	52.00	84.00	
race	122	0.00	0.00	0.00	0.00	
edu	122	1.97	1.10	1.00	4.00	
**HRS**						
time	15964	-3.46	2.99	-10.00	0.00	
purpose	15964	3.77	0.76	1.00	5.00	0.86
heart	15951	0.39	0.49	0.00	1.00	
smoker	14654	0.09	0.29	0.00	1.00	
srh	15954	2.67	1.00	1.00	5.00	
gender	15964	0.56	0.50	0.00	1.00	
race	15964	0.26	0.44	0.00	1.00	
edu	15964	12.07	3.31	0.00	17.00	
b.age	15964	68.07	9.08	34.00	100.00	
**KGSS**						
time	88	-1.50	1.12	-3.00	0.00	
purpose	205	3.60	1.42	1.00	5.00	0.92
smoker	90	0.23	0.43	0.00	1.00	
srh	205	2.99	1.17	1.00	5.00	
b.age	205	62.21	12.29	32.00	85.00	
gender	205	0.59	0.49	0.00	1.00	
edu	205	3.19	1.79	1.00	7.00	
**MIDJA**						
time	107	-2.50	1.94	-4.00	0.00	
purpose	107	3.31	0.64	1.57	5.00	0.87
heart	102	0.24	0.43	0.00	1.00	
smoker	97	0.32	0.47	0.00	1.00	
srh	107	2.81	0.91	1.00	5.00	
b.age	107	63.78	9.50	38.00	79.00	
gender	107	0.33	0.47	0.00	1.00	
edu	107	4.45	2.32	1.00	8.00	
**MIDUS**						
time	582	-12.39	6.62	-18.00	0.00	
purpose	582	3.81	0.85	1.22	5.00	0.80
heart	582	0.23	0.42	0.00	1.00	
smoker	538	0.14	0.35	0.00	1.00	
srh	579	2.70	0.98	1.00	5.00	
gender	582	0.42	0.49	0.00	1.00	
b.age	582	54.72	11.81	24.00	75.00	
race	582	0.13	0.33	0.00	1.00	
edu	582	6.50	2.72	1.00	12.00	
**SWAN**						
time	2150	-7.25	3.31	-10.00	0.00	
purpose	2150	4.66	0.73	1.00	5.00	0.94
heart	2147	0.08	0.27	0.00	1.00	
smoker	2106	0.18	0.39	0.00	1.00	
srh	2102	2.65	0.95	1.00	5.00	
b.age	2150	46.93	3.78	40.00	55.00	
edu	2150	2.85	1.22	1.00	5.00	
race	2150	0.68	0.47	0.00	1.00	
**WLS**						
time	986	-10.60	7.71	-19.00	0.00	
purpose	986	3.88	0.77	1.11	5.00	0.92
heart	974	0.32	0.47	0.00	1.00	
srh	904	3.48	0.76	1.00	5.00	
smoker	236	0.09	0.29	0.00	1.00	
gender	986	0.47	0.50	0.00	1.00	
b.age	986	54.22	4.61	41.00	77.00	
edu	986	13.40	2.20	8.00	20.00	

4 Regression Models: Purpose influencing trajectories

We identify which studies have with outcomes. We also removed the studies ALSA and ELSA from the smoking status analyses for having too few smokers after recommendation by peer reviewers.

```
study.outcomes = describe.df %>%
  group_by(study) %>%
  summarize(srh = ifelse("srh" %in% var, 1, 0),
            smoker = ifelse("smoker" %in% var, 1, 0),
            heart = ifelse("heart" %in% var, 1, 0))

srh.names = study.outcomes$study[which(study.outcomes$srh == 1)]
smoker.names = study.outcomes$study[which(study.outcomes$smoker == 1)]
smoker.names = smoker.names[!(smoker.names %in% c("cloc", "alsa", "elsa"))]
heart.names = study.outcomes$study[which(study.outcomes$heart == 1)]
```

4.1 Self-rated health

```
# gather regression estimates into a data frame
# each row is one study
# each column is one regression model
srh.data = data.frame(study = srh.names)
srh.data$standard = lapply(X = srh.names,
                     FUN = function(x) get(paste0(x,"_out"))$regression$purpose$srh)

srh.data.n = data.frame(study = srh.names)
srh.data.n$npeople = unlist(lapply(X = srh.names,
                     FUN = function(x)
                       length(unique(get(paste0(x,"_out"))$regression$purpose$srh@frame$id))))
srh.data.n$nobs = unlist(lapply(X = srh.names,
                     FUN = function(x)
                       nrow(get(paste0(x,"_out"))$regression$purpose$srh@frame)))

srh.data.n = srh.data.n %>%
  gather(-study, key = "term", value = "estimate")


srh.data = srh.data %>%
  # gather so all regression models are in one column
  gather(key = "model", value = "value", -study) %>%
  # tidy
  mutate(tidy = map(value, broom::tidy)) %>%
  # unnest the tidy (the coefficients become the data frame)
  unnest(tidy) %>%
  full_join(srh.data.n) %>%
  # pull the estimates and standard errors into a single column
  mutate(estimate = ifelse(!is.na(std.error),
                          paste0(printnum(estimate), " (", printnum(std.error), ")"),
                          printnum(estimate)),
         estimate =ifelse(term %in% c("nobs", "npeople"), printnum(estimate, format = "d"), estimate),
         statistic = ifelse(!is.na(statistic), printnum(statistic), NA)) %>%
  dplyr::select(-std.error) %>%
  gather(key = "key", value = "value",
         which(names(.) %in% c("estimate", "statistic"))) %>%
  # merge these two columns
  unite(key, key, study) %>%
  # spread back out. now each of our estimates and se's have their own columns
  spread(key = "key", value = "value") %>%
  mutate(term = factor(term,
                       levels = c("(Intercept)", "gender", "race",
                                  "c.age", "edu", "diabetes", "purpose",
                                  "purpose:diabetes",
                                  "time", "time:purpose",
                                  "sd__(Intercept)",
                                  "cor__(Intercept).time",
                                  "sd__time",
                                  "sd__Observation", "nobs", "npeople" ))) %>%
  arrange(group, term) %>%
  mutate(term = as.character(term),
         term = gsub("\\(Intercept\\)", "Intercept", term),
         term = gsub("diabetes", "Diabetes", term),
         term = gsub("gender", "Gender", term),
         term = gsub("race", "Race", term),
         term = gsub("c.age", "Age", term),
         term = gsub("edu", "Education", term),
         term = gsub("purpose", "Purpose", term),
         term = gsub("time", "Time", term),
         term = gsub("\\:", " x ", term),
         term = gsub("sd__(Intercept)", "$\\\\sigma_{\\\\textrm{Intercept}}$", term),
```

```
         term = gsub("sd__Time", "$\\\\sigma_{\\\\textrm{Time}}$", term),
         term = gsub("cor__(Intercept).Time", "$r_{\\\\textrm{Intercept},\\\\textrm{Time}}$", term),
         term = gsub("sd__Observation", "$\\\\sigma_{\\\\textrm{Residual}}$", term),
         term = gsub("nobs", "$N_{\\\\textrm{obs}}$", term),
         term = gsub("npeople", "$N_{\\\\textrm{people}}$", term))

  fixef.srh = which(srh.data$effect == "fixed")
  rand.srh = which(srh.data$effect == "ran_pars")

srh.data %>%
  dplyr::select(term, paste0(c("estimate_", "statistic_"), rep(srh.names, each = 2))) %>%
  kable(., booktabs = T, caption = "Trajectories of self-rated health", escape = F,
        col.names = c("Term", rep(c("$b(SE)$", "$t$"), length(srh.names)))) %>%
  kable_styling(font_size = 7) %>%
  add_header_above(c(" ",
                     "ACL" = 2, "ALSA" = 2, "ELSA" = 2,
                     "HRS" = 2, "KGSS" = 2, "MIDJA" = 2,
                     "MIDUS" = 2, "SWAN" = 2, "WLS" = 2)) %>%
  landscape() %>%
  group_rows("Fixed Effects", min(fixef.srh), max(fixef.srh)) %>%
  group_rows("Random Effects", min(rand.srh), max(rand.srh)) %>%
  column_spec(c(2,4,6,8,10,12,14,16,18), width = "3em")
```

	ACL		ALSA		ELSA		HRS		KGSS		MIDJA		MIDUS		SWAN		WLS	
Term	$b(SE)$	t	$b(SE)$	t	$b(SE)$	t	$b(SE)$	t	$b(SE)$	t	$b(SE)$	t	$b(SE)$	t	$b(SE)$	t	$b(SE)$	t
**Random Effects**																		
σ_{Residual}	0.71		0.74		0.51		0.62		1.05		0.70		0.68		0.52		0.51	
$\sigma_{\text{Intercept}}$	0.83		0.81		0.76		0.75		0.12		0.60		0.82		0.78		0.68	
$r_{\text{Intercept,Time}}$	1.00		1.00		-0.40		0.40		-1.00		0.51		0.68		0.42		0.72	
σ_{Time}	0.02		0.08		0.06		0.05		0.01		0.04		0.02		0.07		0.03	
**Fixed Effects**																		
Intercept	1.21	1.95	1.14	1.78	4.81	3.00	0.78	9.22	2.85	3.52	2.51	3.20	1.66	3.66	1.40	3.27	2.33	7.25
	(0.62)		(0.64)		(1.61)		(0.08)		(0.81)		(0.78)		(0.45)		(0.43)		(0.32)	
Gender	-0.04	-0.26	-0.04	-0.25	0.19	0.40	-0.04	-1.66	0.42	1.49	-0.09	-0.37	-0.11	-1.12			0.14	2.31
	(0.17)		(0.15)		(0.48)		(0.02)		(0.28)		(0.24)		(0.10)				(0.06)	
Race	-0.18	-1.11					-0.15	-5.53					-0.19	-1.38	-0.34	-5.67		
	(0.16)						(0.03)						(0.14)		(0.06)			
Age	0.13	1.62	0.14	1.16	-0.12	-0.52	0.02	1.73	0.16	0.85	0.08	0.73	-0.05	-1.26	-0.07	-1.05	0.02	0.27
	(0.08)		(0.12)		(0.24)		(0.01)		(0.19)		(0.10)		(0.04)		(0.07)		(0.06)	
Education	0.10	2.78	0.02	0.66	0.00	-0.01	0.06	17.50	0.04	0.45	0.02	0.44	0.03	1.83	0.18	7.35	0.03	2.21
	(0.04)		(0.02)		(0.23)		(0.00)		(0.10)		(0.05)		(0.02)		(0.02)		(0.01)	
Purpose	0.08	0.91	0.27	2.20	-0.65	-1.58	0.27	15.29	0.03	0.22	0.05	0.23	0.18	1.62	0.17	1.91	0.13	1.89
	(0.09)		(0.12)		(0.41)		(0.02)		(0.15)		(0.23)		(0.11)		(0.09)		(0.07)	
Time	-0.01	-0.29	-0.09	-0.41	0.58	3.54	-0.02	-1.45	0.84	3.66	0.35	1.72	-0.02	-0.62	-0.02	-0.40	0.02	1.04
	(0.04)		(0.22)		(0.16)		(0.01)		(0.23)		(0.20)		(0.03)		(0.04)		(0.02)	
Time x Purpose	0.00	0.05	0.00	-0.03	-0.17	-3.42	0.00	-1.26	-0.15	-2.07	-0.10	-1.69	0.00	0.06	0.00	0.13	-0.01	-1.88
	(0.01)		(0.06)		(0.05)		(0.00)		(0.07)		(0.06)		(0.01)		(0.01)		(0.00)	
N_{obs}	264.00		350.00		55.00		15,954.00		88.00		107.00		579.00		2,102.00		904.00	
N_{people}	132.00		145.00		17.00		4,529.00		22.00		67.00		308.00		1,014.00		439.00	

Table 2: Trajectories of self-rated health

4.2 Smoking status

```
# gather regression estimates into a data frame
# each row is one study
# each column is one regression model
smoker.data = data.frame(study = smoker.names)
smoker.data$standard = lapply(X = smoker.names,
                     FUN = function(x) get(paste0(x,"_out"))$regression$purpose$smoker)

smoker.data.n = data.frame(study = smoker.names)
smoker.data.n$npeople = unlist(lapply(X = smoker.names,
                     FUN = function(x)
                       length(unique(get(paste0(x,"_out"))$regression$purpose$smoker@frame$id))))
smoker.data.n$nobs = unlist(lapply(X = smoker.names,
                     FUN = function(x)
                       nrow(get(paste0(x,"_out"))$regression$purpose$smoker@frame)))

smoker.data.n = smoker.data.n %>%
  gather(-study, key = "term", value = "estimate")


smoker.data = smoker.data %>%
  # gather so all regression models are in one column
  gather(key = "model", value = "value", -study) %>%
  # tidy
  mutate(tidy = map(value, broom::tidy)) %>%
  # unnest the tidy (the coefficients become the data frame)
  unnest(tidy)

smoker.data$OR[smoker.data$effect == "fixed"] = printnum(exp(smoker.data$estimate[smoker.data$effect == "fixed"]))

smoker.data = smoker.data %>%
  full_join(smoker.data.n) %>%
  # pull the estimates and standard errors into a single column
  mutate(estimate = ifelse(!is.na(std.error),
                         paste0(printnum(estimate), " (", printnum(std.error), ")"),
                         printnum(estimate)),
         estimate =ifelse(term %in% c("nobs", "npeople"),
                        printnum(estimate, format = "d"), estimate),
         statistic = ifelse(!is.na(statistic), printnum(statistic), NA)) %>%
  dplyr::select(-std.error, -p.value) %>%
  gather(key = "key", value = "value",
         which(names(.) %in% c("estimate", "statistic", "OR"))) %>%
  # merge these two columns
  unite(key, key, study) %>%
  # spread back out. now each of our estimates and se's have their own columns
  spread(key = "key", value = "value") %>%
  mutate(term = factor(term,
                     levels = c("(Intercept)", "gender", "race",
                                "c.age", "edu", "diabetes", "purpose",
                                "purpose:diabetes",
                                "time", "time:purpose",
                                "sd__(Intercept)",
                                "cor__(Intercept).time",
                                "sd__time",
                                "sd__Observation", "nobs", "npeople" ))) %>%
  arrange(group, term) %>%
  mutate(term = as.character(term),
         term = gsub("\\(Intercept\\)", "Intercept", term),
         term = gsub("diabetes", "Diabetes", term),
         term = gsub("gender", "Gender", term),
         term = gsub("race", "Race", term),
         term = gsub("c.age", "Age", term),
         term = gsub("edu", "Education", term),
         term = gsub("purpose", "Purpose", term),
         term = gsub("time", "Time", term),
         term = gsub("\\:", " x ", term),
         term = gsub("sd__(Intercept)", "$\\\\sigma_{\\\\textrm{Intercept}}$", term),
         term = gsub("sd__Time", "$\\\\sigma_{\\\\textrm{Time}}$", term),
         term = gsub("cor__(Intercept).Time", "$r_{\\\\textrm{Intercept},\\\\textrm{Time}}$", term),
         term = gsub("sd__Observation", "$\\\\sigma_{\\\\textrm{Residual}}$", term),
         term = gsub("nobs", "$N_{\\\\textrm{obs}}$", term),
         term = gsub("npeople", "$N_{\\\\textrm{people}}$", term))

  fixef.smoker = which(smoker.data$effect == "fixed")
  rand.smoker = which(smoker.data$effect == "ran_pars")
```

```
smoker.data %>%
  dplyr::select(term, paste0(c("estimate_", "OR_", "statistic_"),
                             rep(smoker.names, each = 3))) %>%
  kable(., booktabs = T,
        caption = "Trajectories of smoking status",
        escape = F,
        col.names = c("Term", rep(c("$b(SE)$","OR","$t$"),
                                  length(smoker.names)))) %>%
  kable_styling(font_size = 7) %>%
  add_header_above(c(" ",
                     "ACL" = 3, "HRS" = 3,
                     "KGSS" = 3, "MIDJA" = 3,
                     "MIDUS" = 3, "SWAN" = 3, "WLS" = 3)) %>%
  landscape() %>%
  group_rows("Fixed Effects", min(fixef.smoker), max(fixef.smoker)) %>%
  group_rows("Random Effects", min(rand.smoker), max(rand.smoker)) %>%
  column_spec(seq(from = 2, by = 3, length.out = 8), width = "3em")
```

	ACL			HRS			KGSS			MIDJA			MIDUS			SWAN			WLS		
Term	$b(SE)$	OR	t	$b(SE)$	OR	t	$b(SE)$	OR	t	$b(SE)$	OR	t	$b(SE)$	OR	t	$b(SE)$	OR	t	$b(SE)$	OR	t
**Random Effects**																					
$\sigma_{\text{Intercept}}$	1.47			1.84			43.92			2.47			2.89			24.78			36.32		
$r_{\text{Intercept,Time}}$	-1.00			-0.94			1.00			-1.00			-1.00			1.00			1.00		
σ_{Time}	1.42			5.35			21.26			0.78			0.02			2.34			1.98		
**Fixed Effects**																					
Intercept	3.15	23.30	1.02	0.33	1.39	0.75	-6.93	0.00	-0.12	2.96	19.39	0.77	-1.49	0.22	-0.49	-17.28	0.00	-0.70	6.66	779.06	0.10
	(3.10)			(0.45)			(59.16)			(3.87)			(3.03)			(24.53)			(67.47)		
Gender	-0.81	0.45	-0.93	0.21	1.23	1.46	1.34	3.82	0.44	-3.57	0.03	-1.88	0.21	1.24	0.37				-0.52	0.60	-0.99
	(0.87)			(0.14)			(3.08)			(1.90)			(0.57)						(0.53)		
Race	-0.36	0.69	-0.40	-0.08	0.93	-0.47							-0.61	0.54	-0.75	-0.37	0.69	-1.83			
	(0.90)			(0.16)									(0.81)			(0.20)					
Age	-1.55	0.21	-2.36	-0.77	0.46	-9.62	-0.43	0.65	-0.21	-0.44	0.64	-0.60	-0.80	0.45	-3.23	-0.39	0.68	-1.74	-0.29	0.75	-0.77
	(0.66)			(0.08)			(2.06)			(0.74)			(0.25)			(0.22)			(0.37)		
Education	-0.16	0.85	-0.88	-0.06	0.94	-2.59	-2.67	0.07	-1.48	-0.28	0.76	-0.88	-0.09	0.92	-0.76	-0.35	0.70	-3.88	-0.29	0.75	-2.00
	(0.19)			(0.02)			(1.80)			(0.31)			(0.11)			(0.09)			(0.15)		
Purpose	-0.36	0.70	-0.80	-0.19	0.82	-2.12	2.66	14.36	0.15	-0.67	0.51	-0.54	-0.44	0.64	-0.57	-0.15	0.86	-0.03	-5.10	0.01	-0.26
	(0.45)			(0.09)			(17.68)			(1.24)			(0.78)			(5.18)			(19.29)		
Time	0.38	1.46	0.38	0.68	1.97	0.79	1.65	5.20	0.06	-0.45	0.64	-0.32	-0.05	0.95	-0.31	-1.73	0.18	-0.71	0.22	1.25	0.06
	(0.99)			(0.86)			(29.70)			(1.42)			(0.17)			(2.45)			(3.74)		
Time x Purpose	-0.03	0.97	-0.14	0.17	1.19	0.77	-0.63	0.53	-0.07	0.03	1.03	0.08	-0.01	0.99	-0.25	-0.01	0.99	-0.02	-0.27	0.76	-0.26
	(0.22)			(0.23)			(8.79)			(0.43)			(0.05)			(0.52)			(1.07)		
N_{obs}	204.00			14,654.00			44.00			97.00			538.00			2,106.00			236.00		
N_{people}	132.00			4,349.00			22.00			63.00			308.00			1,013.00			171.00		

Table 3: Trajectories of smoking status

4.3 Heart condition status

```
# gather regression estimates into a data frame
# each row is one study
# each column is one regression model
heart.data = data.frame(study = heart.names)
heart.data$standard = lapply(X = heart.names,
                    FUN = function(x) get(paste0(x,"_out"))$regression$purpose$heart)

heart.data.n = data.frame(study = heart.names)
heart.data.n$npeople = unlist(lapply(X = heart.names,
                    FUN = function(x)
                      length(unique(get(paste0(x,"_out"))$regression$purpose$heart@frame$id))))
heart.data.n$nobs = unlist(lapply(X = heart.names,
                    FUN = function(x)
                      nrow(get(paste0(x,"_out"))$regression$purpose$heart@frame)))

heart.data.n = heart.data.n %>%
  gather(-study, key = "term", value = "estimate")


heart.data = heart.data %>%
  # gather so all regression models are in one column
  gather(key = "model", value = "value", -study) %>%
  # tidy
  mutate(tidy = map(value, broom::tidy)) %>%
  # unnest the tidy (the coefficients become the data frame)
  unnest(tidy)

heart.data$OR[heart.data$effect == "fixed"] = printnum(exp(heart.data$estimate[heart.data$effect == "fixed"]))

heart.data = heart.data %>%
  full_join(heart.data.n) %>%
  # pull the estimates and standard errors into a single column
  mutate(estimate = ifelse(!is.na(std.error),
                           paste0(printnum(estimate), " (", printnum(std.error), ")"),
                           printnum(estimate)),
         estimate =ifelse(term %in% c("nobs", "npeople"),
                          printnum(estimate, int =T), estimate),
         statistic = ifelse(!is.na(statistic), printnum(statistic), NA)) %>%
  dplyr::select(-std.error, -p.value) %>%
  gather(key = "key", value = "value",
         which(names(.) %in% c("estimate", "statistic", "OR"))) %>%
  # merge these two columns
  unite(key, key, study) %>%
  # spread back out. now each of our estimates and se's have their own columns
  spread(key = "key", value = "value") %>%
  mutate(term = factor(term,
                      levels = c("(Intercept)", "gender", "race",
                                 "c.age", "edu", "diabetes", "purpose",
                                 "purpose:diabetes",
                                 "time", "time:purpose",
                                 "sd__(Intercept)",
                                 "cor__(Intercept).time",
                                 "sd__time",
                                 "sd__Observation", "nobs", "npeople" ))) %>%
  arrange(group, term) %>%
  mutate(term = as.character(term),
         term = gsub("\\(Intercept\\)", "Intercept", term),
         term = gsub("diabetes", "Diabetes", term),
         term = gsub("gender", "Gender", term),
         term = gsub("race", "Race", term),
         term = gsub("c.age", "Age", term),
         term = gsub("edu", "Education", term),
         term = gsub("purpose", "Purpose", term),
         term = gsub("time", "Time", term),
         term = gsub("\\:", " x ", term),
         term = gsub("sd__(Intercept)", "$\\\\sigma_{\\\\textrm{Intercept}}$", term),
         term = gsub("sd__Time", "$\\\\sigma_{\\\\textrm{Time}}$", term),
         term = gsub("cor__(Intercept).Time", "$r_{\\\\textrm{Intercept},\\\\textrm{Time}}$", term),
         term = gsub("sd__Observation", "$\\\\sigma_{\\\\textrm{Residual}}$", term),
         term = gsub("nobs", "$N_{\\\\textrm{obs}}$", term),
         term = gsub("npeople", "$N_{\\\\textrm{people}}$", term))

  fixef.heart = which(heart.data$effect == "fixed")
  rand.heart = which(heart.data$effect == "ran_pars")
```

```r
heart.data %>%
  dplyr::select(term, paste0(c("estimate_", "OR_", "statistic_"),
                            rep(heart.names, each = 3))) %>%
  kable(., booktabs = T, caption = "Trajectories of heart conditions", escape =F,
        col.names = c("Term", rep(c("$b(SE)$","OR","$t$"), length(heart.names)))) %>%
  kable_styling(font_size = 7) %>%
  add_header_above(c(" ",
                     "ACL" = 3, "ELSA" = 3,
                     "HRS" = 3, "MIDJA" = 3,
                     "MIDUS" = 3, "SWAN" = 3, "WLS" = 3)) %>%
  landscape() %>%
  group_rows("Fixed Effects", min(fixef.heart), max(fixef.heart)) %>%
  group_rows("Random Effects", min(rand.heart), max(rand.heart)) %>%
  column_spec(seq(from = 2, by = 3, length.out = 7), width = "3em")
```

	ACL			ELSA			HRS			MIDJA			MIDUS			SWAN			WLS		
Term	$b(SE)$	OR	t	$b(SE)$	OR	t	$b(SE)$	OR	t	$b(SE)$	OR	t	$b(SE)$	OR	t	$b(SE)$	OR	t	$b(SE)$	OR	t
**Random Effects**																					
$\sigma_{\text{Intercept}}$	0.63			1.69			4.29			28.86			2.28			5.19			2.80		
$r_{\text{Intercept,Time}}$	-1.00			-1.00			-0.79			1.00			1.00			1.00			1.00		
σ_{Time}	2.63			6.33			2.03			7.00			0.08			0.46			0.11		
**Fixed Effects**																					
Intercept	-6.41 (2.70)	0.00	-2.38	-44.64 (23.55)	0.00	-1.90	0.56 (0.53)	1.75	1.05	-31.14 (52.04)	0.00	-0.60	-0.95 (1.54)	0.39	-0.62	0.54 (3.85)	1.72	0.14	1.68 (1.33)	5.37	1.26
Gender	-0.17 (0.67)	0.84	-0.26	14.70 (7.76)	2,410,979.56	1.89	-0.47 (0.16)	0.63	-2.86	-0.13 (0.78)	0.87	-0.17	-0.13 (0.30)	0.88	-0.41				-0.75 (0.23)	0.47	-3.26
Race	1.11 (0.67)	3.03	1.66				-0.63 (0.19)	0.53	-3.34				-0.68 (0.52)	0.51	-1.31	-0.03 (0.21)	0.97	-0.13			
Age	0.43 (0.31)	1.54	1.39	5.22 (2.99)	184.05	1.74	0.52 (0.09)	1.68	5.91	1.01 (0.44)	2.75	2.27	0.33 (0.14)	1.40	2.41	1.00 (0.24)	2.73	4.16	0.53 (0.24)	1.70	2.23
Education	0.27 (0.15)	1.31	1.76	4.49 (2.33)	89.14	1.93	0.00 (0.03)	1.00	-0.05	0.03 (0.16)	1.03	0.17	-0.01 (0.06)	0.99	-0.14	-0.33 (0.09)	0.72	-3.49	-0.03 (0.05)	0.97	-0.63
Purpose	0.00 (0.30)	1.00	0.01	4.39 (3.25)	80.67	1.35	-0.29 (0.11)	0.75	-2.68	6.45 (13.83)	630.47	0.47	0.30 (0.38)	1.35	0.80	-1.15 (0.85)	0.32	-1.35	-0.27 (0.29)	0.76	-0.94
Time	1.21 (3.11)	3.35	0.39	-2.99 (11.18)	0.05	-0.27	0.59 (0.28)	1.81	2.13	-7.05 (12.94)	0.00	-0.55	0.02 (0.10)	1.02	0.22	0.19 (0.39)	1.20	0.48	0.02 (0.07)	1.02	0.30
Time x Purpose	-0.11 (0.69)	0.90	-0.16	0.43 (3.53)	1.53	0.12	0.10 (0.07)	1.10	1.33	1.59 (3.44)	4.91	0.46	0.03 (0.02)	1.03	1.19	-0.12 (0.09)	0.89	-1.37	0.02 (0.02)	1.02	0.96
N_{obs}	255.00			73.00			15,951.00			102.00			582.00			2,147.00			974.00		
N_{people}	132.00			17.00			4,530.00			64.00			308.00			1,014.00			439.00		

Table 4: Trajectories of heart conditions

5 Meta-analysis

5.1 Self-rated health

First we extract the data from the individual models tested.

```
srh.meta.data = data.frame(study = srh.names)
srh.meta.data$average = lapply(X = srh.names,
                     FUN = function(x) get(paste0(x,"_out"))$regression$average$srh)
srh.meta.data$purpose = lapply(X = srh.names,
                     FUN = function(x) get(paste0(x,"_out"))$regression$purpose$srh)
srh.meta.data$low.purpose = lapply(X = srh.names,
                     FUN = function(x) get(paste0(x,"_out"))$regression$low.purpose$srh)
srh.meta.data$hgh.purpose = lapply(X = srh.names,
                     FUN = function(x) get(paste0(x,"_out"))$regression$hgh.purpose$srh)

srh.meta.data$n = unlist(
  lapply(X = srh.names,
         FUN = function(x)
           length(unique(get(paste0(x,"_out"))$regression$purpose$srh@frame$id))))


srh.meta.data = srh.meta.data %>%
  gather(key = "model", value = "value", -study, -n) %>%
  mutate(tidy = map(value, broom::tidy, conf.int = TRUE)) %>%
  unnest(tidy, .drop =T) %>%
  filter(
    grepl("Intercept", term) |
    grepl("time", term) |
    grepl("purpose", term)) %>%
  filter(effect == "fixed") %>%
  dplyr::select(-effect) %>%
  mutate(term = gsub("\\(Intercept\\)", "intercept", term)) %>%
  gather(key = "statistic", value = "value", estimate:conf.high) %>%
  unite(model, model, statistic) %>%
  spread(key = "model", value = "value")
```

Next we use the metafor package to estimate the weighted average effect and heterogeneity.

```
# average intercept (not controlling for purpose)
srh.average.intcp <- srh.meta.data %>%
  filter(term == "intercept") %>%
  rma(yi = average_estimate,
      sei = average_std.error,
      ni = n,
      method = "REML",
      slab = study,
      data = .)

# average slope (not controlling for purpose)
srh.average.slope <- srh.meta.data %>%
  filter(term == "time") %>%
  rma(yi = average_estimate,
      sei = average_std.error,
      ni = n,
      method = "REML",
      slab = study,
      data = .)

# effect of purpose on intercept
srh.purpose.intcp <- srh.meta.data %>%
  filter(term == "purpose") %>%
  rma(yi = purpose_estimate,
      sei = purpose_std.error,
      ni = n,
      method = "REML",
      slab = study,
      data = .)

# effect of purpose on slope
srh.purpose.slope <- srh.meta.data %>%
  filter(term == "time:purpose") %>%
  rma(yi = purpose_estimate,
      sei = purpose_std.error,
      ni = n,
```

```
      method = "REML",
      slab = study,
      data = .)
```

Additionally, we use the meta-analysis package to estimate the weighted average slopes at different levels of purpose, for the purpose of summarizing in the forest plot.

```
#slope estimate at low levels of purpose
srh.lowpurpose <- srh.meta.data %>%
  filter(term == "time") %>%
  rma(yi = low.purpose_estimate,
      sei = low.purpose_std.error,
      ni = n,
      method = "REML",
      slab = study,
      data = .)

#slope estimate at high levels of purpose
srh.highpurpose <- srh.meta.data %>%
  filter(term == "time") %>%
  rma(yi = hgh.purpose_estimate,
      sei = hgh.purpose_std.error,
      ni = n,
      method = "REML",
      slab = study,
      data = .)
```

5.2 Smoking status

We removed the studies ALSA and ELSA for having too few smokers after recommendation by peer reviewers.

```
smoker.meta.data = data.frame(study = smoker.names)
smoker.meta.data = data.frame(study = smoker.names)
smoker.meta.data$average = lapply(X = smoker.names,
                     FUN = function(x) get(paste0(x,"_out"))$regression$average$smoker)
smoker.meta.data$purpose = lapply(X = smoker.names,
                     FUN = function(x) get(paste0(x,"_out"))$regression$purpose$smoker)
smoker.meta.data$low.purpose = lapply(X = smoker.names,
                     FUN = function(x) get(paste0(x,"_out"))$regression$low.purpose$smoker)
smoker.meta.data$hgh.purpose = lapply(X = smoker.names,
                     FUN = function(x) get(paste0(x,"_out"))$regression$hgh.purpose$smoker)

smoker.meta.data$n = unlist(
  lapply(X = smoker.names,
         FUN = function(x)
           length(unique(get(paste0(x,"_out"))$regression$purpose$smoker@frame$id))))


smoker.meta.data = smoker.meta.data %>%
  gather(key = "model", value = "value", -study, -n) %>%
  mutate(tidy = map(value, broom::tidy, conf.int = TRUE)) %>%
  unnest(tidy, .drop =T) %>%
  filter(
    grepl("Intercept", term) |
    grepl("time", term) |
    grepl("purpose", term)) %>%
  filter(effect == "fixed") %>%
  dplyr::select(-effect) %>%
  mutate(term = gsub("\\(Intercept\\)", "intercept", term)) %>%
  gather(key = "statistic", value = "value", estimate:conf.high) %>%
  unite(model, model, statistic) %>%
  spread(key = "model", value = "value")
```

Next we use the metafor package to estimate the weighted average effect and heterogeneity.

```
# average intercept (not controlling for purpose)
smoker.average.intcp <- smoker.meta.data %>%
  filter(term == "intercept") %>%
  rma(yi = average_estimate,
      sei = average_std.error,
```

```
      ni = n,
      method = "REML",
      slab = study,
      data = .)

# average slope (not controlling for purpose)
smoker.average.slope <- smoker.meta.data %>%
  filter(term == "time") %>%
  rma(yi = average_estimate,
      sei = average_std.error,
      ni = n,
      method = "REML",
      slab = study,
      data = .)

# effect of purpose on intercept
smoker.purpose.intcp <- smoker.meta.data %>%
  filter(term == "purpose") %>%
  rma(yi = purpose_estimate,
      sei = purpose_std.error,
      ni = n,
      method = "REML",
      slab = study,
      data = .)

# effect of purpose on slope
smoker.purpose.slope <- smoker.meta.data %>%
  filter(term == "time:purpose") %>%
  rma(yi = purpose_estimate,
      sei = purpose_std.error,
      ni = n,
      method = "REML",
      slab = study,
      data = .)
```

Additionally, we use the meta-analysis package to estimate the weighted average slopes at different levels of purpose and diabetes status, for the purpose of summarizing in the forest plot.

```
#slope estimate at low levels of purpose
smoker.lowpurpose <- smoker.meta.data %>%
  filter(term == "time") %>%
  rma(yi = low.purpose_estimate,
      sei = low.purpose_std.error,
      ni = n,
      method = "REML",
      slab = study,
      data = .)

#slope estimate at high levels of purpose
smoker.highpurpose <- smoker.meta.data %>%
  filter(term == "time") %>%
  rma(yi = hgh.purpose_estimate,
      sei = hgh.purpose_std.error,
      ni = n,
      method = "REML",
      slab = study,
      data = .)
```

5.3 Heart condition status

```
heart.meta.data = data.frame(study = heart.names)
heart.meta.data$average = lapply(X = heart.names,
                     FUN = function(x) get(paste0(x,"_out"))$regression$average$heart)
heart.meta.data$purpose = lapply(X = heart.names,
                     FUN = function(x) get(paste0(x,"_out"))$regression$purpose$heart)
heart.meta.data$low.purpose = lapply(X = heart.names,
                     FUN = function(x) get(paste0(x,"_out"))$regression$low.purpose$heart)
heart.meta.data$hgh.purpose = lapply(X = heart.names,
                     FUN = function(x) get(paste0(x,"_out"))$regression$hgh.purpose$heart)

heart.meta.data$n = unlist(
  lapply(X = heart.names,
```

```
        FUN = function(x)
          length(unique(get(paste0(x,"_out"))$regression$purpose$heart@frame$id))))


heart.meta.data = heart.meta.data %>%
  gather(key = "model", value = "value", -study, -n) %>%
  mutate(tidy = map(value, broom::tidy, conf.int = TRUE)) %>%
  unnest(tidy, .drop =T) %>%
  filter(
    grepl("Intercept", term) |
    grepl("time", term) |
    grepl("purpose", term)) %>%
  filter(effect == "fixed") %>%
  dplyr::select(-effect) %>%
  mutate(term = gsub("\\(Intercept\\)", "intercept", term)) %>%
  gather(key = "statistic", value = "value", estimate:conf.high) %>%
  unite(model, model, statistic) %>%
  spread(key = "model", value = "value")
```

Next we use the metafor package to estimate the weighted average effect and heterogeneity.

```
# average intercept (not controlling for purpose)
heart.average.intcp <- heart.meta.data %>%
  filter(term == "intercept") %>%
  rma(yi = average_estimate,
      sei = average_std.error,
      ni = n,
      method = "REML",
      slab = study,
      data = .)

# average slope (not controlling for purpose)
heart.average.slope <- heart.meta.data %>%
  filter(term == "time") %>%
  rma(yi = average_estimate,
      sei = average_std.error,
      ni = n,
      method = "REML",
      slab = study,
      data = .)

# effect of purpose on intercept
heart.purpose.intcp <- heart.meta.data %>%
  filter(term == "purpose") %>%
  rma(yi = purpose_estimate,
      sei = purpose_std.error,
      ni = n,
      method = "REML",
      slab = study,
      data = .)

# effect of purpose on slope
heart.purpose.slope <- heart.meta.data %>%
  filter(term == "time:purpose") %>%
  rma(yi = purpose_estimate,
      sei = purpose_std.error,
      ni = n,
      method = "REML",
      slab = study,
      data = .)
```

Additionally, we use the meta-analysis package to estimate the weighted average slopes at different levels of purpose and diabetes status, for the purpose of summarizing in the forest plot.

```
#slope estimate at low levels of purpose
heart.lowpurpose <- heart.meta.data %>%
  filter(term == "time") %>%
  rma(yi = low.purpose_estimate,
      sei = low.purpose_std.error,
      ni = n,
      method = "REML",
      slab = study,
      data = .)

#slope estimate at high levels of purpose
```

```
heart.highpurpose <- heart.meta.data %>%
  filter(term == "time") %>%
  rma(yi = hgh.purpose_estimate,
      sei = hgh.purpose_std.error,
      ni = n,
      method = "REML",
      slab = study,
      data = .)
```

5.4 Summary

We summarize the effects of the meta-analyses here.

```
meta.summary = data.frame(outcome = rep(c("Self-Rated Health", "Smoking", "Heart Disease"), each = 4),
                              model = rep(rep(c("average", "purpose"), each = 2), 3),
                              coef = rep(c("intercept", "slope"), 6))
meta.summary$rma = list(srh.average.intcp, srh.average.slope,
                        srh.purpose.intcp, srh.purpose.slope,
                        smoker.average.intcp, smoker.average.slope,
                        smoker.purpose.intcp, smoker.purpose.slope,
                        heart.average.intcp, heart.average.slope,
                        heart.purpose.intcp, heart.purpose.slope)

meta.summary %>%
  mutate(sum = map(rma, function(x) coef(summary(x)))) %>%
  dplyr::select(-rma) %>%
  unnest() %>%
  mutate(outcome = factor(outcome, levels = c("Self-Rated Health", "Smoking", "Heart Disease"))) %>%
  arrange(outcome, coef) %>%
  unite(coef, model, coef) %>%
  mutate(coef = factor(coef)) %>%
  mutate(coef = factor(coef,
                       labels = c("Average intercept", "Average slope",
                                  "Effect of purpose on intercept", "Effect of purpose on slope"))) %>%
  dplyr::select(-outcome) %>%
  mutate(pval = papaja::printp(pval)) %>%
  kable(., booktabs = TRUE, escape = FALSE, caption = "Meta-analysis models.",
        col.names = c("Coefficient", "b", "$SE_b$", "Z", "p", "$CI_{lb}$", "$CI_{ub}$"),
        format = "latex", digits = 2) %>%
  kable_styling() %>%
  #column_spec(1, width = "30em")  %>%
  group_rows("Self-Rated Health", 1, 4) %>%
  group_rows("Smoking", 5, 8) %>%
  group_rows("Heart Disease", 9, 12)
```

5.5 Forest plot

We combine all values into a single data frame for summary purposes.

```
srh.meta.data$outcome = "srh"
smoker.meta.data$outcome = "smoker"
heart.meta.data$outcome = "heart"

all.meta <- full_join(srh.meta.data, smoker.meta.data) %>%
  full_join(heart.meta.data) %>%
  filter(grepl("time", term)) %>%
  gather("key", "value", -study, -n, -group, -term, -outcome) %>%
  filter((term == "time:purpose" & grepl("^purpose", key)) |
         (term == "time" & grepl("^hgh", key)) |
         (term == "time" & grepl("^low", key))) %>%
  filter(!grepl("statistic", key)) %>%
  filter(!is.na(value) & !is.null(value)) %>%
  dplyr::select(-term) %>%
  spread(key, value)
```

We calculate the odds ratios for the smoker and heart disease models.

```
all.meta = all.meta %>%
  mutate(OR = printnum(exp(purpose_estimate))) %>%
  mutate(OR = ifelse(outcome == "srh", NA, OR))
```

Coefficient	b	SE_b	Z	p	CI_{lb}	CI_{ub}
**Self-Rated Health**						
Average intercept	2.20	0.15	14.37	¡ .001	1.90	2.50
Effect of purpose on intercept	0.17	0.04	4.34	¡ .001	0.10	0.25
Average slope	-0.02	0.00	-3.75	¡ .001	-0.02	-0.01
Effect of purpose on slope	0.00	0.00	-2.05	.040	-0.01	0.00
**Smoking**						
Average intercept	-3.72	3.14	-1.19	.235	-9.87	2.42
Effect of purpose on intercept	-0.21	0.09	-2.32	.020	-0.38	-0.03
Average slope	-0.18	0.43	-0.41	.679	-1.02	0.67
Effect of purpose on slope	-0.01	0.04	-0.13	.900	-0.09	0.08
**Heart Disease**						
Average intercept	-1.98	1.34	-1.48	.138	-4.60	0.64
Effect of purpose on intercept	-0.23	0.09	-2.50	.012	-0.41	-0.05
Average slope	0.18	0.24	0.78	.438	-0.28	0.65
Effect of purpose on slope	0.02	0.01	1.46	.145	-0.01	0.05

Table 5: Meta-analysis models.

We format the estimates and confidence intervals for the simple slopes at different levels of purpose.

```
all.meta = all.meta %>%
  mutate(outcome = factor(outcome, levels = c("srh", "smoker", "heart"))) %>%
  arrange(outcome, study) %>%
  mutate(sig.l = ifelse(low.purpose_conf.high < 0 | low.purpose_conf.low > 0 , "*", ""),
         sig.h = ifelse(hgh.purpose_conf.high < 0 | hgh.purpose_conf.low > 0 , "*", "")) %>%
  mutate(
    across(contains("p.value"), printp)) %>%
  mutate(low_slope = paste0(printnum(low.purpose_estimate),
                            sig.l,
                            " [",
                            printnum(low.purpose_conf.low),
                            ", ",
                            printnum(low.purpose_conf.high),
                            "]"),
         hgh_slope = paste0(printnum(hgh.purpose_estimate),
                            sig.h,
                            " [",
                            printnum(hgh.purpose_conf.low),
                            ", ",
                            printnum(hgh.purpose_conf.high),
                            "]"))
```

Now we can build the figure. First we find the horizontal limits of the graph.

```
extra.columns = all.meta %>%
  dplyr::select(study, outcome, n, which(grepl("_slope$", names(.)) | grepl("OR$", names(.)))) %>%
  gather(key  = "key", value = "value", -n, -study, -outcome) %>%
  spread(key = "key", value = "value") %>%
  arrange(outcome, study) %>%
  dplyr::select(n, low_slope, hgh_slope)

num.ci = length(which(grepl("slope", names(extra.columns))))

# find plot limits
max.ci = order(all.meta$purpose_conf.high, decreasing = T)
max.ci = all.meta$purpose_conf.high[max.ci][2] #midja CI too large for bounds
min.ci = order(all.meta$purpose_conf.low)
min.ci = all.meta$purpose_conf.low[min.ci][2] #midja CI too large for bounds

range = max.ci-min.ci
# the lower bound must give room for all the extra columns we have
# most columns need only a little space, but columns containing confidence intervals need more
lower = min.ci-(range*(.5*ncol(extra.columns) + .25*num.ci))
```

```
# the upper bound gives us a little space
upper = max.ci+range
```

Next we figure out where the extra columns go. We do so by dividing up the space between the minimum CI and the lower bound.

```
#how much room needed
needed.space = ncol(extra.columns) + num.ci

pos = min.ci-lower
pos = pos/(needed.space + 1)
column.position = 1:ncol(extra.columns)
for(i in 1:length(column.position)){
  if(grepl("slope", names(extra.columns)[i])) {
    column.position[(i):ncol(extra.columns)] = column.position[(i):ncol(extra.columns)] + .5
    }
}

positions = lower + pos*column.position + pos
```

Now we calculated the needed space along the y-axis. First we determine how many extra rows we need between each ”set” of analyses. We need two extra rows for specific information: the weighted average estimates and a label. From there, we added extra rows until the figure looked appealing. We ended up with 5 extra rows.

From there, we figure out which rows will be filled with each set of analyses. It’s important to remember that rows start from the bottom and as you move up the figure, the row number increases.

```
extra.space = 5

# which rows are for heart disease
total.heart = length(which(all.meta$outcome == "heart"))
rows.heart = c(1, total.heart)

# which rows are for smokers
total.smoker = length(which(all.meta$outcome == "smoker"))
# skip row for diabetes summary, space, and heart title
rows.smoker = c(rows.heart[2] + extra.space, rows.heart[2] + extra.space + total.smoker - 1)

# which rows are for self-rated health
total.srh = length(which(all.meta$outcome == "srh"))
# skip row for hbp summary, space, title
rows.srh = c(rows.smoker[2] + extra.space, rows.smoker[2] + extra.space + total.srh - 1)
```

The last step before beginning the plot is to set the font size and formatting. ‘font = 1‘ is the non-bold, non-italic font. The final chunk builds the forest plot. Comments are included throughout as a guide.

```
cex.value = .55
par(font = 1)

forest(all.meta$purpose_estimate, #estimate
       all.meta$purpose_std.error^2, #variance
       xlim = c(lower, upper), #limits of x-axis, full figure
       alim = c(floor(min.ci), ceiling(max.ci)), #limits of CI
       ylim = c(-1, nrow(all.meta) + extra.space*2 + 2), # limits of y-axis
       cex = cex.value, #font size
       slab = all.meta$study, #study label
       # which rows do I fill in. Remember, data frame goes from top to bottom, but row numbers on
       # figure go from bottom to top
       rows = c(rows.srh[2]:rows.srh[1],
                rows.smoker[2]:rows.smoker[1],
                rows.heart[2]:rows.heart[1]),
       # what extra information is added?
       ilab = cbind(extra.columns[,1], extra.columns[,2], extra.columns[,3]),
       # where does extra information go?
       ilab.xpos = positions[1:3])

# add weighted average effects polygon
addpoly(srh.purpose.slope, row=rows.srh[1]-1,
        cex = cex.value, mlab = "", col = "blue")
addpoly(smoker.purpose.slope, row=rows.smoker[1]-1,
```

```
        cex = cex.value, mlab = "", col = "blue")
addpoly(heart.purpose.slope, row=rows.heart[1]-1,
        cex = cex.value, mlab = "", col = "blue")

### add text with Q-value, dfs, p-value, and I^2 statistic for subgroups
text(x = lower, y = rows.srh[1]-2.25, pos=4, cex = cex.value,
     bquote(paste("RE Model for self-rated health (Q = ",
                  .(formatC(srh.purpose.slope$QE, digits=2, format="f")),
                  ", df = ", .(srh.purpose.slope$k -
                               srh.purpose.slope$p),
                  ", p = ", .(formatC(srh.purpose.slope$QEp, digits=2, format="f")),
                  "; ", I^2, " = ",
                  .(formatC(srh.purpose.slope$I2, digits=1, format="f")), "%)")))

text(x = lower, y = rows.smoker[1]-2.25, pos=4, cex = cex.value,
     bquote(paste("RE Model for smoking status (Q = ",
                  .(formatC(smoker.purpose.slope$QE, digits=2, format="f")),
                  ", df = ", .(smoker.purpose.slope$k -
                               smoker.purpose.slope$p),
                  ", p = ", .(formatC(smoker.purpose.slope$QEp, digits=2, format="f")),
                  "; ", I^2, " = ",
                  .(formatC(smoker.purpose.slope$I2, digits=1, format="f")), "%)")))

text(x = lower, y = rows.heart[1]-2.25, pos=4, cex = cex.value,
     bquote(paste("RE Model for heart disease status (Q = ",
                  .(formatC(heart.purpose.slope$QE, digits=2, format="f")),
                  ", df = ", .(heart.purpose.slope$k -
                               heart.purpose.slope$p),
                  ", p = ", .(formatC(heart.purpose.slope$QEp, digits=2, format="f")),
                  "; ", I^2, " = ",
                  .(formatC(heart.purpose.slope$I2, digits=1, format="f")), "%)")))
par(font = 2)

# add "overall"" label to each set of analyses
text(lower, y = c(rows.srh[1]-1, rows.smoker[1]-1, rows.heart[1]-1),
     pos = 4, cex = cex.value, "Overall")

# add overall sample sizes for each set of analyses
srh.n = srh.meta.data %>%
  filter(term == "time:purpose") %>%
  summarize(n = sum(n))
text(x = positions[which(names(extra.columns) == "n")],
     y = rows.srh[1]-1, cex = cex.value,
     printnum(sum(srh.n[1,1]), format = "d"))
smoker.n = smoker.meta.data %>%
  filter(term == "time:purpose") %>%
  summarize(n = sum(n))
text(x = positions[which(names(extra.columns) == "n")],
     y = rows.smoker[1]-1, cex = cex.value,
     printnum(sum(smoker.n[1,1]), format = "d"))
heart.n = heart.meta.data %>%
  filter(term == "time:purpose") %>%
  summarize(n = sum(n))
text(x = positions[which(names(extra.columns) == "n")],
     y = rows.heart[1]-1, cex = cex.value,
     printnum(sum(heart.n[1,1]), int =T))

# add weighted average simple slopes for each set of analyses

conf = expand.grid(outcome = c("srh", "smoker", "heart"), slope = c("low", "hgh"))
conf$rma = list(srh.lowpurpose, smoker.lowpurpose, heart.lowpurpose,
                srh.highpurpose, smoker.highpurpose, heart.highpurpose)
conf = conf %>%
  mutate(est = map(rma, "b"),
         lb = map(rma, "ci.lb"),
         ub = map(rma, "ci.ub")) %>%
  mutate(ci = paste0(printnum(est), " [", printnum(lb), ", ", printnum(ub), "]"))

text(x = positions[which(names(extra.columns) == "low_slope")],
     y = c(rows.srh[1]-1, rows.smoker[1]-1, rows.heart[1]-1),
     cex = cex.value,
     c(conf$ci[c(1:3)]))

text(x = positions[which(names(extra.columns) == "hgh_slope")],
     y = c(rows.srh[1]-1, rows.smoker[1]-1, rows.heart[1]-1),
     cex = cex.value,
     c(conf$ci[c(4:6)]))
```

```
# column labels
text(lower, nrow(all.meta) + extra.space*2 + 1,
     "Study", cex = cex.value, pos = 4)
text(upper, nrow(all.meta) + extra.space*2 + 1,
     "Estimate of purpose x slope interaction [95% CI]", cex = cex.value, pos=2)
text(positions[which(names(extra.columns) == "n")], nrow(all.meta) + extra.space*2 + 1,
     "Sample Size", cex = cex.value)

text(positions[grepl("hgh", names(extra.columns))],
     nrow(all.meta) + extra.space*2 + 1,
     cex = cex.value, "Slope at high purpose")

text(positions[grepl("low", names(extra.columns))],
     nrow(all.meta) + extra.space*2 + 1,
     cex = cex.value, "Slope at low purpose")

#bold and italic font, plus bigger text
par(font = 4)

# outcome labels
text(lower, rows.srh[2] + 1,
     "Self-Rated Health", cex = cex.value, pos = 4)
text(lower, rows.smoker[2] + 1,
     "Smoking Status", cex = cex.value, pos = 4)
text(lower, rows.heart[2] + 1,
     "Heart Disease Status", cex = cex.value, pos = 4)
```

Study	Sample Size	Slope at low purpose	Slope at high purpose	Estimate of purpose x slope interaction [95% CI]
Self−Rated Health				
acl	132	−0.01 [−0.03, 0.02]	−0.01 [−0.03, 0.02]	0.00 [−0.02, 0.02]
alsa	145	−0.09 [−0.26, 0.07]	−0.10 [−0.24, 0.04]	−0.00 [−0.12, 0.12]
elsa	17	0.14* [0.06, 0.23]	−0.07 [−0.15, 0.01]	−0.17 [−0.27, −0.07]
hrs	4529	−0.03* [−0.03, −0.02]	−0.03* [−0.04, −0.02]	−0.00 [−0.01, 0.00]
kgss	22	0.63* [0.35, 0.91]	0.20 [−0.08, 0.48]	−0.15 [−0.29, −0.01]
midja	67	0.08 [−0.03, 0.19]	−0.04 [−0.14, 0.05]	−0.10 [−0.22, 0.02]
midus	308	−0.01 [−0.03, 0.00]	−0.01* [−0.03, 0.00]	0.00 [−0.01, 0.01]
swan	1014	−0.01 [−0.03, 0.01]	−0.01 [−0.03, 0.01]	0.00 [−0.02, 0.02]
wls	439	−0.01 [−0.02, 0.00]	−0.02* [−0.03, −0.01]	−0.01 [−0.02, 0.00]
**Overall**	**6673**	**0.03 [−0.03, 0.10]**	**−0.02 [−0.03, −0.01]**	−0.00 [−0.01, −0.00]
RE Model for self−rated health (Q = 19.70, df = 8, p = 0.01; I^2 = 0.3%)				
Smoking Status				
acl	132	0.28 [−0.38, 0.94]	0.21 [−0.45, 0.87]	−0.03 [−0.47, 0.41]
hrs	4349	1.19* [0.72, 1.67]	1.47* [0.95, 1.98]	0.17 [−0.27, 0.62]
kgss	22	0.72 [−34.14, 35.59]	−1.10 [−26.71, 24.51]	−0.63 [−17.85, 16.59]
midja	63	−0.36 [−1.06, 0.34]	−0.32 [−1.10, 0.47]	0.03 [−0.82, 0.89]
midus	308	−0.09 [−0.19, 0.02]	−0.11* [−0.21, 0.00]	−0.01 [−0.10, 0.08]
swan	1013	−1.77* [−2.75, −0.79]	−1.97* [−3.08, −0.85]	−0.01 [−1.02, 1.00]
wls	171	−0.52 [−1.78, 0.73]	−0.96 [−3.55, 1.63]	−0.27 [−2.37, 1.82]
**Overall**	**6058**	**−0.14 [−0.91, 0.63]**	**−0.15 [−1.09, 0.79]**	−0.01 [−0.09, 0.08]
RE Model for smoking status (Q = 0.73, df = 6, p = 0.99; I^2 = 0.0%)				
Heart Disease Status				
acl	132	0.86 [−1.16, 2.89]	0.63 [−1.09, 2.35]	−0.11 [−1.45, 1.24]
elsa	17	−1.89 [−7.60, 3.82]	−1.36 [−8.10, 5.39]	0.43 [−6.50, 7.35]
hrs	4530	0.88* [0.73, 1.04]	1.04* [0.88, 1.19]	0.10 [−0.05, 0.24]
midja	64	−2.84 [−11.32, 5.64]	−0.84 [−6.18, 4.50]	1.59 [−5.14, 8.32]
midus	308	0.11* [0.04, 0.17]	0.16* [0.10, 0.21]	0.03 [−0.02, 0.08]
swan	1014	−0.29* [−0.47, −0.11]	−0.45* [−0.68, −0.22]	−0.12 [−0.29, 0.05]
wls	439	0.07* [0.03, 0.11]	0.10* [0.06, 0.14]	0.02 [−0.02, 0.05]
**Overall**	**6504**	**0.20 [−0.25, 0.65]**	**0.22 [−0.32, 0.76]**	0.02 [−0.01, 0.05]
RE Model for heart disease status (Q = 4.12, df = 6, p = 0.66; I^2 = 0.0%)				

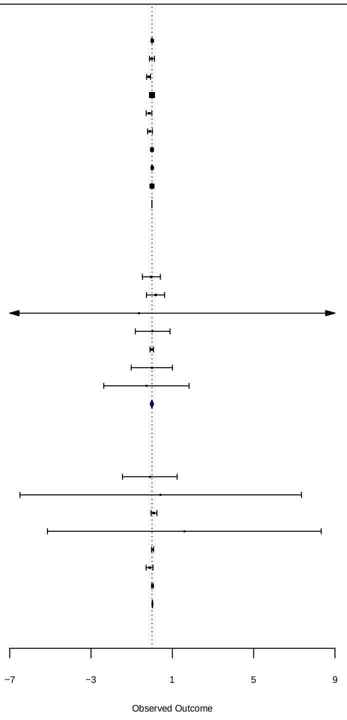

6 Moderation

Only the relationship of purpose to self-rated health intercepts and slopes showed significant variability across samples. Therefore, we will examine moderators of these two effects and no others.

First, we must add additional information to the study level data frame.

```
study.level$year[study.level$study == "acl"] = 2002
study.level$year[study.level$study == "alsa"] = 1994
study.level$year[study.level$study == "elsa"] = 2004
study.level$year[study.level$study == "hrs"] = 2006
study.level$year[study.level$study == "kgss"] = 2009
study.level$year[study.level$study == "midja"] = 2008
study.level$year[study.level$study == "midus"] = 1994
study.level$year[study.level$study == "swan"] = 1996
study.level$year[study.level$study == "wls"] = 1992

study.level$american = ifelse(study.level$study %in%
                                c("alsa","elsa","kgss","midja"),0,1)
```

6.1 Self-rated health intercept

```
#moderated by age
srh.intcp_age <- srh.meta.data %>%
  full_join(study.level) %>%
  filter(term == "purpose") %>%
  rma(yi = purpose_estimate,
      sei = purpose_std.error,
      ni = n,
      method = "REML",
      slab = study,
      mods = age,
      data = .)

## Joining with `by = join_by(study)`

srh.intcp_age

##
## Mixed-Effects Model (k = 9; tau^2 estimator: REML)
##
## tau^2 (estimated amount of residual heterogeneity):     0.0023 (SE = 0.0055)
## tau (square root of estimated tau^2 value):             0.0477
## I^2 (residual heterogeneity / unaccounted variability): 20.13%
## H^2 (unaccounted variability / sampling variability):   1.25
## R^2 (amount of heterogeneity accounted for):            53.16%
##
## Test for Residual Heterogeneity:
## QE(df = 7) = 10.0585, p-val = 0.1853
##
## Test of Moderators (coefficient 2):
## QM(df = 1) = 2.0459, p-val = 0.1526
##
## Model Results:
##
##          estimate      se     zval    pval    ci.lb   ci.ub
```

```
## intrcpt   -0.1515  0.2414  -0.6277  0.5302  -0.6246  0.3216
## mods       0.0055  0.0039   1.4303  0.1526  -0.0020  0.0131
##
## ---
## Signif. codes:  0 '***' 0.001 '**' 0.01 '*' 0.05 '.' 0.1 ' ' 1
```

```
#moderated by country
srh.intcp_american <- srh.meta.data %>%
  full_join(study.level) %>%
  filter(term == "purpose") %>%
  rma(yi = purpose_estimate,
      sei = purpose_std.error,
      ni = n,
      method = "REML",
      slab = study,
      mods = american,
      data = .)
```

```
## Joining with `by = join_by(study)`
```

```
srh.intcp_american
```

```
##
## Mixed-Effects Model (k = 9; tau^2 estimator: REML)
##
## tau^2 (estimated amount of residual heterogeneity):     0.0050 (SE = 0.0068)
## tau (square root of estimated tau^2 value):             0.0709
## I^2 (residual heterogeneity / unaccounted variability): 43.71%
## H^2 (unaccounted variability / sampling variability):   1.78
## R^2 (amount of heterogeneity accounted for):            0.00%
##
## Test for Residual Heterogeneity:
## QE(df = 7) = 14.9831, p-val = 0.0362
##
## Test of Moderators (coefficient 2):
## QM(df = 1) = 0.5313, p-val = 0.4660
##
## Model Results:
##
##          estimate      se    zval    pval    ci.lb   ci.ub
## intrcpt    0.1096  0.0961  1.1415  0.2537  -0.0786  0.2979
## mods       0.0772  0.1059  0.7289  0.4660  -0.1304  0.2848
##
## ---
## Signif. codes:  0 '***' 0.001 '**' 0.01 '*' 0.05 '.' 0.1 ' ' 1
```

```
#moderated by number of purpose items
srh.intcp_items <- srh.meta.data %>%
  full_join(study.level) %>%
  filter(term == "purpose") %>%
  rma(yi = purpose_estimate,
      sei = purpose_std.error,
      ni = n,
      method = "REML",
      slab = study,
      mods = number_items,
      data = .)
```

```
## Joining with `by = join_by(study)`

srh.intcp_items

##
## Mixed-Effects Model (k = 9; tau^2 estimator: REML)
##
## tau^2 (estimated amount of residual heterogeneity):     0.0041 (SE = 0.0069)
## tau (square root of estimated tau^2 value):             0.0638
## I^2 (residual heterogeneity / unaccounted variability): 32.05%
## H^2 (unaccounted variability / sampling variability):   1.47
## R^2 (amount of heterogeneity accounted for):            15.96%
##
## Test for Residual Heterogeneity:
## QE(df = 7) = 11.9898, p-val = 0.1009
##
## Test of Moderators (coefficient 2):
## QM(df = 1) = 0.5783, p-val = 0.4470
##
## Model Results:
##
##          estimate      se    zval    pval    ci.lb   ci.ub
## intrcpt    0.1146  0.0910  1.2594  0.2079  -0.0638  0.2930
## mods       0.0127  0.0166  0.7605  0.4470  -0.0200  0.0453
##
## ---
## Signif. codes:  0 '***' 0.001 '**' 0.01 '*' 0.05 '.' 0.1 ' ' 1

#moderated by year at baseline
srh.intcp_year <- srh.meta.data %>%
  full_join(study.level) %>%
  filter(term == "purpose") %>%
  rma(yi = purpose_estimate,
      sei = purpose_std.error,
      ni = n,
      method = "REML",
      slab = study,
      mods = year,
      data = .)

## Joining with `by = join_by(study)`

srh.intcp_year

##
## Mixed-Effects Model (k = 9; tau^2 estimator: REML)
##
## tau^2 (estimated amount of residual heterogeneity):     0.0062 (SE = 0.0089)
## tau (square root of estimated tau^2 value):             0.0785
## I^2 (residual heterogeneity / unaccounted variability): 37.21%
## H^2 (unaccounted variability / sampling variability):   1.59
## R^2 (amount of heterogeneity accounted for):            0.00%
##
## Test for Residual Heterogeneity:
## QE(df = 7) = 12.3453, p-val = 0.0898
##
## Test of Moderators (coefficient 2):
```

```
## QM(df = 1) = 0.0019, p-val = 0.9655
##
## Model Results:
##
##          estimate       se     zval    pval     ci.lb    ci.ub
## intrcpt   -0.4341  13.9640  -0.0311  0.9752  -27.8030  26.9349
## mods       0.0003   0.0070   0.0432  0.9655   -0.0134   0.0140
##
## ---
## Signif. codes:  0 '***' 0.001 '**' 0.01 '*' 0.05 '.' 0.1 ' ' 1
```

```
#moderated by maximum number of years at follow up
srh.intcp_follow <- srh.meta.data %>%
  full_join(study.level) %>%
  filter(term == "purpose") %>%
  rma(yi = purpose_estimate,
      sei = purpose_std.error,
      ni = n,
      method = "REML",
      slab = study,
      mods = maxwaves,
      data = .)
```

## Joining with 'by = join_by(study)'

```
srh.intcp_follow
```

```
##
## Mixed-Effects Model (k = 9; tau^2 estimator: REML)
##
## tau^2 (estimated amount of residual heterogeneity):     0.0055 (SE = 0.0074)
## tau (square root of estimated tau^2 value):             0.0741
## I^2 (residual heterogeneity / unaccounted variability): 44.03%
## H^2 (unaccounted variability / sampling variability):   1.79
## R^2 (amount of heterogeneity accounted for):            0.00%
##
## Test for Residual Heterogeneity:
## QE(df = 7) = 15.4742, p-val = 0.0304
##
## Test of Moderators (coefficient 2):
## QM(df = 1) = 0.0423, p-val = 0.8371
##
## Model Results:
##
##          estimate      se     zval    pval    ci.lb   ci.ub
## intrcpt    0.1892  0.0963   1.9644  0.0495   0.0004  0.3781  *
## mods      -0.0016  0.0078  -0.2056  0.8371  -0.0169  0.0137
##
## ---
## Signif. codes:  0 '***' 0.001 '**' 0.01 '*' 0.05 '.' 0.1 ' ' 1
```

```
#moderated by all
srh.intcp_all <- srh.meta.data %>%
  full_join(study.level) %>%
  filter(term == "purpose") %>%
  rma(yi = purpose_estimate,
      sei = purpose_std.error,
```

```
      ni = n,
      method = "REML",
      slab = study,
      mods = age + american + number_items + year + maxwaves,
      data = .)

## Joining with `by = join_by(study)`

srh.intcp_all

##
## Mixed-Effects Model (k = 9; tau^2 estimator: REML)
##
## tau^2 (estimated amount of residual heterogeneity):     0.0008 (SE = 0.0054)
## tau (square root of estimated tau^2 value):             0.0290
## I^2 (residual heterogeneity / unaccounted variability): 6.92%
## H^2 (unaccounted variability / sampling variability):   1.07
## R^2 (amount of heterogeneity accounted for):            82.67%
##
## Test for Residual Heterogeneity:
## QE(df = 7) = 9.2028, p-val = 0.2384
##
## Test of Moderators (coefficient 2):
## QM(df = 1) = 4.3305, p-val = 0.0374
##
## Model Results:
##
##          estimate      se     zval    pval     ci.lb    ci.ub
## intrcpt   -9.7708  4.7980  -2.0364  0.0417  -19.1747  -0.3669  *
## mods       0.0048  0.0023   2.0810  0.0374    0.0003   0.0093  *
##
## ---
## Signif. codes:  0 '***' 0.001 '**' 0.01 '*' 0.05 '.' 0.1 ' ' 1
```

6.2 Self-rated health slope

```
#moderated by age
srh.slope_age <- srh.meta.data %>%
  full_join(study.level) %>%
  filter(term == "time:purpose") %>%
  rma(yi = purpose_estimate,
      sei = purpose_std.error,
      ni = n,
      method = "REML",
      slab = study,
      mods = age,
      data = .)

## Joining with `by = join_by(study)`

srh.slope_age

##
## Mixed-Effects Model (k = 9; tau^2 estimator: REML)
##
```

```
## tau^2 (estimated amount of residual heterogeneity):     0.0000 (SE = 0.0000)
## tau (square root of estimated tau^2 value):             0.0013
## I^2 (residual heterogeneity / unaccounted variability): 1.91%
## H^2 (unaccounted variability / sampling variability):   1.02
## R^2 (amount of heterogeneity accounted for):            0.00%
##
## Test for Residual Heterogeneity:
## QE(df = 7) = 19.6928, p-val = 0.0063
##
## Test of Moderators (coefficient 2):
## QM(df = 1) = 0.0204, p-val = 0.8865
##
## Model Results:
##
##          estimate      se     zval    pval    ci.lb   ci.ub
## intrcpt   -0.0016  0.0180  -0.0873  0.9305  -0.0369  0.0337
## mods      -0.0000  0.0003  -0.1428  0.8865  -0.0006  0.0005
##
## ---
## Signif. codes:  0 '***' 0.001 '**' 0.01 '*' 0.05 '.' 0.1 ' ' 1
```

```
#moderated by country
srh.slope_american <- srh.meta.data %>%
  full_join(study.level) %>%
  filter(term == "time:purpose") %>%
  rma(yi = purpose_estimate,
      sei = purpose_std.error,
      ni = n,
      method = "REML",
      slab = study,
      mods = american,
      data = .)
```

```
## Joining with `by = join_by(study)`
```

```
srh.slope_american
```

```
##
## Mixed-Effects Model (k = 9; tau^2 estimator: REML)
##
## tau^2 (estimated amount of residual heterogeneity):     0.0000 (SE = 0.0000)
## tau (square root of estimated tau^2 value):             0.0005
## I^2 (residual heterogeneity / unaccounted variability): 0.54%
## H^2 (unaccounted variability / sampling variability):   1.01
## R^2 (amount of heterogeneity accounted for):            0.00%
##
## Test for Residual Heterogeneity:
## QE(df = 7) = 6.6353, p-val = 0.4678
##
## Test of Moderators (coefficient 2):
## QM(df = 1) = 13.0625, p-val = 0.0003
##
## Model Results:
##
##          estimate      se     zval    pval    ci.lb    ci.ub
## intrcpt   -0.1102  0.0294  -3.7455  0.0002  -0.1678  -0.0525  ***
## mods       0.1066  0.0295   3.6142  0.0003   0.0488   0.1644  ***
```

```
##
## ---
## Signif. codes:  0 '***' 0.001 '**' 0.01 '*' 0.05 '.' 0.1 ' ' 1

#moderated by number of purpose items
srh.slope_items <- srh.meta.data %>%
  full_join(study.level) %>%
  filter(term == "time:purpose") %>%
  rma(yi = purpose_estimate,
      sei = purpose_std.error,
      ni = n,
      method = "REML",
      slab = study,
      mods = number_items,
      data = .)

## Joining with `by = join_by(study)`
## Warning:  Fisher scoring algorithm may have gotten stuck at a local maximum.
## Setting tau^2 = 0.  Check the profile likelihood plot with profile().

srh.slope_items

##
## Mixed-Effects Model (k = 9; tau^2 estimator: REML)
##
## tau^2 (estimated amount of residual heterogeneity):     0 (SE = 0.0000)
## tau (square root of estimated tau^2 value):             0
## I^2 (residual heterogeneity / unaccounted variability): 0.00%
## H^2 (unaccounted variability / sampling variability):   1.00
## R^2 (amount of heterogeneity accounted for):            100.00%
##
## Test for Residual Heterogeneity:
## QE(df = 7) = 18.5573, p-val = 0.0097
##
## Test of Moderators (coefficient 2):
## QM(df = 1) = 1.1411, p-val = 0.2854
##
## Model Results:
##
##          estimate      se     zval    pval    ci.lb   ci.ub
## intrcpt    0.0027  0.0067   0.4051  0.6854  -0.0104  0.0158
## mods      -0.0011  0.0011  -1.0682  0.2854  -0.0032  0.0009
##
## ---
## Signif. codes:  0 '***' 0.001 '**' 0.01 '*' 0.05 '.' 0.1 ' ' 1

#moderated by year at baseline
srh.slope_year <- srh.meta.data %>%
  full_join(study.level) %>%
  filter(term == "time:purpose") %>%
  rma(yi = purpose_estimate,
      sei = purpose_std.error,
      ni = n,
      method = "REML",
      slab = study,
      mods = year,
      data = .)
```

```
## Joining with `by = join_by(study)`
## Warning:  Fisher scoring algorithm may have gotten stuck at a local maximum.
## Setting tau^2 = 0.  Check the profile likelihood plot with profile().

srh.slope_year

##
## Mixed-Effects Model (k = 9; tau^2 estimator: REML)
##
## tau^2 (estimated amount of residual heterogeneity):     0 (SE = 0.0000)
## tau (square root of estimated tau^2 value):             0
## I^2 (residual heterogeneity / unaccounted variability): 0.00%
## H^2 (unaccounted variability / sampling variability):   1.00
## R^2 (amount of heterogeneity accounted for):            100.00%
##
## Test for Residual Heterogeneity:
## QE(df = 7) = 19.6454, p-val = 0.0064
##
## Test of Moderators (coefficient 2):
## QM(df = 1) = 0.0530, p-val = 0.8179
##
## Model Results:
##
##          estimate      se     zval    pval    ci.lb   ci.ub
## intrcpt   -0.1489  0.6285  -0.2368  0.8128  -1.3808  1.0830
## mods       0.0001  0.0003   0.2303  0.8179  -0.0005  0.0007
##
## ---
## Signif. codes:  0 '***' 0.001 '**' 0.01 '*' 0.05 '.' 0.1 ' ' 1

#moderated by maximum number of years at follow up
srh.slope_follow <- srh.meta.data %>%
  full_join(study.level) %>%
  filter(term == "time:purpose") %>%
  rma(yi = purpose_estimate,
      sei = purpose_std.error,
      ni = n,
      method = "REML",
      slab = study,
      mods = maxwaves,
      data = .)

## Joining with `by = join_by(study)`
## Warning:  Fisher scoring algorithm may have gotten stuck at a local maximum.
## Setting tau^2 = 0.  Check the profile likelihood plot with profile().

srh.slope_follow

##
## Mixed-Effects Model (k = 9; tau^2 estimator: REML)
##
## tau^2 (estimated amount of residual heterogeneity):     0 (SE = 0.0000)
## tau (square root of estimated tau^2 value):             0
## I^2 (residual heterogeneity / unaccounted variability): 0.00%
## H^2 (unaccounted variability / sampling variability):   1.00
## R^2 (amount of heterogeneity accounted for):            100.00%
##
```

```
## Test for Residual Heterogeneity:
## QE(df = 7) = 19.6475, p-val = 0.0064
##
## Test of Moderators (coefficient 2):
## QM(df = 1) = 0.0509, p-val = 0.8215
##
## Model Results:
##
##          estimate      se     zval    pval    ci.lb   ci.ub
## intrcpt   -0.0027  0.0065  -0.4124  0.6800  -0.0155  0.0101
## mods      -0.0001  0.0005  -0.2256  0.8215  -0.0010  0.0008
##
## ---
## Signif. codes:  0 '***' 0.001 '**' 0.01 '*' 0.05 '.' 0.1 ' ' 1
```

```
#moderated by all
srh.slope_all <- srh.meta.data %>%
  full_join(study.level) %>%
  filter(term == "time:purpose") %>%
  rma(yi = purpose_estimate,
      sei = purpose_std.error,
      ni = n,
      method = "REML",
      slab = study,
      mods = age + american + number_items + year + maxwaves,
      data = .)
```

```
## Joining with `by = join_by(study)`
## Warning:  Fisher scoring algorithm may have gotten stuck at a local maximum.
## Setting tau^2 = 0.  Check the profile likelihood plot with profile().
```

```
srh.slope_all
```

```
##
## Mixed-Effects Model (k = 9; tau^2 estimator: REML)
##
## tau^2 (estimated amount of residual heterogeneity):     0 (SE = 0.0000)
## tau (square root of estimated tau^2 value):             0
## I^2 (residual heterogeneity / unaccounted variability): 0.00%
## H^2 (unaccounted variability / sampling variability):   1.00
## R^2 (amount of heterogeneity accounted for):            100.00%
##
## Test for Residual Heterogeneity:
## QE(df = 7) = 19.6716, p-val = 0.0063
##
## Test of Moderators (coefficient 2):
## QM(df = 1) = 0.0268, p-val = 0.8699
##
## Model Results:
##
##          estimate      se     zval    pval    ci.lb   ci.ub
## intrcpt    0.0575  0.3760   0.1529  0.8785  -0.6794  0.7943
## mods      -0.0000  0.0002  -0.1638  0.8699  -0.0004  0.0003
##
## ---
## Signif. codes:  0 '***' 0.001 '**' 0.01 '*' 0.05 '.' 0.1 ' ' 1
```

```
save(srh.intcp_age, srh.intcp_american, srh.intcp_follow, srh.intcp_items, srh.intcp_year, srh.intcp_all,
     srh.slope_age, srh.slope_american, srh.slope_follow, srh.slope_items, srh.slope_year, srh.slope_all,
     file=here("Study 2/created data/study2mods.Rdata"))
```

7 Regression Models: Purpose influencing quadratic trajectories

Here, we analyze the same models with quadratic terms.

We identify which studies have with outcomes. We also removed the studies ALSA and ELSA from the smoking status analyses for having too few smokers after recommendation by peer reviewers.

```
study.outcomes = describe.df %>%
  group_by(study) %>%
  summarize(srh = ifelse("srh" %in% var, 1, 0),
            smoker = ifelse("smoker" %in% var, 1, 0),
            heart = ifelse("heart" %in% var, 1, 0))

srh.names = study.outcomes$study[which(study.outcomes$srh == 1)]
smoker.names = study.outcomes$study[which(study.outcomes$smoker == 1)]
smoker.names = smoker.names[!(smoker.names %in% c("cloc", "alsa", "elsa"))]
heart.names = study.outcomes$study[which(study.outcomes$heart == 1)]
```

7.1 Self-rated health

```
# gather regression estimates into a data frame
# each row is one study
# each column is one regression model
srh.data = data.frame(study = srh.names)
srh.data$standard = lapply(X = srh.names,
                     FUN = function(x) get(paste0(x,"_out"))$regression$purpose$srh)

srh.data.n = data.frame(study = srh.names)
srh.data.n$npeople = unlist(lapply(X = srh.names,
                     FUN = function(x)
                       length(unique(get(paste0(x,"_out"))$regression$purpose$srh@frame$id))))
srh.data.n$nobs = unlist(lapply(X = srh.names,
                     FUN = function(x)
                       nrow(get(paste0(x,"_out"))$regression$purpose$srh@frame)))

srh.data.n = srh.data.n %>%
  gather(-study, key = "term", value = "estimate")


srh.data = srh.data %>%
  # gather so all regression models are in one column
  gather(key = "model", value = "value", -study) %>%
  # tidy
  mutate(tidy = map(value, broom::tidy)) %>%
  # unnest the tidy (the coefficients become the data frame)
  unnest(tidy) %>%
  full_join(srh.data.n) %>%
  # pull the estimates and standard errors into a single column
  mutate(estimate = ifelse(!is.na(std.error),
                          paste0(printnum(estimate), " (", printnum(std.error), ")"),
                          printnum(estimate)),
         estimate =ifelse(term %in% c("nobs", "npeople"), printnum(estimate, format = "d"), estimate),
         statistic = ifelse(!is.na(statistic), printnum(statistic), NA)) %>%
  dplyr::select(-std.error) %>%
  gather(key = "key", value = "value",
         which(names(.) %in% c("estimate", "statistic"))) %>%
  # merge these two columns
  unite(key, key, study) %>%
  # spread back out. now each of our estimates and se's have their own columns
  spread(key = "key", value = "value") %>%
  mutate(term = factor(term,
                       levels = c("(Intercept)", "gender", "race",
                                  "c.age", "edu", "diabetes", "purpose",
                                  "purpose:diabetes",
                                  "time", "I(time^2)",
                                  "time:purpose",
                                  "purpose:I(time^2)",
                                  "sd__(Intercept)",
                                  "sd__time",
                                  "sd__I(time^2)",
                                  "cor__(Intercept).time",
                                  "cor__(Intercept).I(time^2)",
                                  "cor__time.I(time^2)",
```

```
                                   "sd__Observation", "nobs", "npeople" ))) %>%
  arrange(group, term) %>%
  mutate(term = as.character(term),
         term = gsub("\\(Intercept\\)", "Intercept", term),
         term = gsub("diabetes", "Diabetes", term),
         term = gsub("gender", "Gender", term),
         term = gsub("race", "Race", term),
         term = gsub("c.age", "Age", term),
         term = gsub("edu", "Education", term),
         term = gsub("purpose", "Purpose", term),
         #term = gsub("I(time^2)", "£\\\\textrm{time}\\\\^2£", term),
         term = gsub("time", "Time", term),
         term = gsub("I.+2.", "Time2", term),
         term = gsub("\\:", " x ", term),
         term = gsub("sd__(Intercept)", "$\\\\sigma_{\\\\textrm{Intercept}}$", term),
         term = gsub("sd__Time2", "$\\\\sigma_{\\\\textrm{Time2}}$", term),
         term = gsub("sd__Time", "$\\\\sigma_{\\\\textrm{Time}}$", term),
         term = gsub("cor__Time2", "$r_{\\\\textrm{Intercept},\\\\textrm{Time2}}$", term),
         term = gsub("cor__Intercept.Time", "$r_{\\\\textrm{Intercept},\\\\textrm{Time}}$", term),
         term = gsub("cor__Time.Time2", "$r_{\\\\textrm{Time},\\\\textrm{Time2}}$", term),
         term = gsub("sd__Observation", "$\\\\sigma_{\\\\textrm{Residual}}$", term),
         term = gsub("nobs", "$N_{\\\\textrm{obs}}$", term),
         term = gsub("npeople", "$N_{\\\\textrm{people}}$", term))

  fixef.srh = which(srh.data$effect == "fixed")
  rand.srh = which(srh.data$effect == "ran_pars")

srh.data %>%
  dplyr::select(term, paste0(c("estimate_", "statistic_"), rep(srh.names, each = 2))) %>%
  kable(., booktabs = T, caption = "Quadratic trajectories of self-rated health", escape = F,
        col.names = c("Term", rep(c("$b(SE)$", "$t$"), length(srh.names)))) %>%
  kable_styling(font_size = 7) %>%
  add_header_above(c(" ",
                     "ACL" = 2, "ALSA" = 2, "ELSA" = 2,
                     "HRS" = 2, "KGSS" = 2, "MIDJA" = 2,
                     "MIDUS" = 2, "SWAN" = 2, "WLS" = 2)) %>%
  landscape() %>%
  group_rows("Fixed Effects", min(fixef.srh), max(fixef.srh)) %>%
  group_rows("Random Effects", min(rand.srh), max(rand.srh)) %>%
  column_spec(c(2,4,6,8,10,12,14,16,18), width = "3em")
```

Term	ACL $b(SE)$	ACL t	ALSA $b(SE)$	ALSA t	ELSA $b(SE)$	ELSA t	HRS $b(SE)$	HRS t	KGSS $b(SE)$	KGSS t	MIDJA $b(SE)$	MIDJA t	MIDUS $b(SE)$	MIDUS t	SWAN $b(SE)$	SWAN t	WLS $b(SE)$	WLS t
**Random Effects**																		
σ_{Residual}	0.35		0.67		0.46		0.60		0.79		0.49		0.33		0.51		0.38	
$\sigma_{\text{Intercept}}$	1.04		0.77		0.80		0.77		0.73		0.79		0.62		0.75		0.65	
σ_{Time}	0.41		0.94		0.15		0.15		1.48		0.35		0.26		0.13		0.13	
σ_{Time2}	0.03		0.50		0.01		0.01		0.46		0.06		0.01		0.01		0.01	
$r_{\text{Intercept,Time}}$	0.41		-0.11		-0.38		0.34		0.79		0.22		0.03		0.29		0.22	
$r_{\text{Intercept,Time2}}$	0.34		-0.15		-0.74		0.21		0.73		-0.08		-0.02		0.12		0.03	
$r_{\text{Time,Time2}}$	0.99		1.00		0.90		0.96		0.99		0.88		0.98		0.86		0.96	
**Fixed Effects**																		
Intercept	1.18	1.91	1.31	2.07	4.24	2.67	0.72	8.21	2.55	2.98	2.51	3.20	1.50	3.77	1.57	3.66	2.37	7.39
	(0.62)		(0.63)		(1.59)		(0.09)		(0.86)		(0.78)		(0.40)		(0.43)		(0.32)	
Gender	-0.04	-0.26	-0.03	-0.21	0.62	1.36	-0.04	-1.64	0.41	1.45	-0.09	-0.37	-0.10	-1.09			0.15	2.46
	(0.17)		(0.15)		(0.45)		(0.02)		(0.28)		(0.24)		(0.10)				(0.06)	
Race	-0.17	-1.02					-0.16	-5.60					-0.20	-1.41	-0.34	-5.64		
	(0.16)						(0.03)						(0.14)		(0.06)			
Age	0.14	1.70	0.13	1.11	-0.04	-0.15	0.02	1.62	0.17	0.92	0.08	0.73	-0.02	-0.43	-0.07	-1.03	0.00	-0.01
	(0.08)		(0.12)		(0.23)		(0.01)		(0.19)		(0.10)		(0.04)		(0.07)		(0.06)	
Education	0.10	2.85	0.02	0.84	-0.04	-0.16	0.07	17.63	0.05	0.47	0.02	0.44	0.06	3.12	0.18	7.35	0.03	2.08
	(0.04)		(0.02)		(0.22)		(0.00)		(0.10)		(0.05)		(0.02)		(0.02)		(0.01)	
Purpose	0.08	0.85	0.21	1.76	-0.59	-1.43	0.28	14.81	0.02	0.10	0.05	0.23	0.22	2.30	0.13	1.49	0.14	2.14
	(0.09)		(0.12)		(0.41)		(0.02)		(0.17)		(0.23)		(0.09)		(0.09)		(0.07)	
Time	0.73	1.08	1.03	1.22	1.05	2.27	-0.09	-3.07	0.04	0.04	0.35	1.72	-0.04	-0.39	0.14	1.05	0.02	0.35
	(0.67)		(0.84)		(0.46)		(0.03)		(0.94)		(0.20)		(0.11)		(0.13)		(0.06)	
Time2	0.07	1.11	0.54	1.35	0.07	1.20	-0.01	-2.81	-0.27	-0.91			0.00	-0.25	0.01	1.22	0.00	0.08
	(0.07)		(0.40)		(0.06)		(0.00)		(0.30)				(0.01)		(0.01)		(0.00)	
Time x Purpose	-0.16	-1.06	-0.35	-1.47	-0.35	-2.52	0.01	0.85	-0.21	-0.72	-0.10	-1.69	0.01	0.54	-0.03	-1.17	0.00	-0.15
	(0.15)		(0.24)		(0.14)		(0.01)		(0.29)		(0.06)		(0.03)		(0.03)		(0.02)	
Purpose x Time2	-0.02	-1.07	-0.17	-1.49	-0.03	-1.52	0.00	1.42	-0.02	-0.23			0.00	0.49	0.00	-1.25	0.00	0.32
	(0.01)		(0.11)		(0.02)		(0.00)		(0.09)				(0.00)		(0.00)		(0.00)	
N_{obs}	264.00		350.00		55.00		15,954.00		88.00		107.00		579.00		2,102.00		904.00	
N_{people}	132.00		145.00		17.00		4,529.00		22.00		67.00		308.00		1,014.00		439.00	

Table 6: Quadratic trajectories of self-rated health

7.2 Smoking status

```
# gather regression estimates into a data frame
# each row is one study
# each column is one regression model
smoker.data = data.frame(study = smoker.names)
smoker.data$standard = lapply(X = smoker.names,
                     FUN = function(x) get(paste0(x,"_out"))$regression$purpose$smoker)

smoker.data.n = data.frame(study = smoker.names)
smoker.data.n$npeople = unlist(lapply(X = smoker.names,
                     FUN = function(x)
                       length(unique(get(paste0(x,"_out"))$regression$purpose$smoker@frame$id))))
smoker.data.n$nobs = unlist(lapply(X = smoker.names,
                     FUN = function(x)
                       nrow(get(paste0(x,"_out"))$regression$purpose$smoker@frame)))

smoker.data.n = smoker.data.n %>%
  gather(-study, key = "term", value = "estimate")


smoker.data = smoker.data %>%
  # gather so all regression models are in one column
  gather(key = "model", value = "value", -study) %>%
  # tidy
  mutate(tidy = map(value, broom::tidy)) %>%
  # unnest the tidy (the coefficients become the data frame)
  unnest(tidy) %>%
  full_join(smoker.data.n) %>%
  # pull the estimates and standard errors into a single column
  mutate(estimate = ifelse(!is.na(std.error),
                           paste0(printnum(estimate), " (", printnum(std.error), ")"),
                           printnum(estimate)),
         estimate =ifelse(term %in% c("nobs", "npeople"), printnum(estimate, format = "d"), estimate),
         statistic = ifelse(!is.na(statistic), printnum(statistic), NA)) %>%
  dplyr::select(-std.error, -p.value) %>%
  gather(key = "key", value = "value",
         which(names(.) %in% c("estimate", "statistic"))) %>%
  # merge these two columns
  unite(key, key, study) %>%
  # spread back out. now each of our estimates and se's have their own columns
  spread(key = "key", value = "value") %>%
  mutate(term = factor(term,
                       levels = c("(Intercept)", "gender", "race",
                                  "c.age", "edu", "diabetes", "purpose",
                                  "purpose:diabetes",
                                  "time", "I(time^2)",
                                  "time:purpose",
                                  "purpose:I(time^2)",
                                  "sd__(Intercept)",
                                  "sd__time",
                                  "sd__I(time^2)",
                                  "cor__(Intercept).time",
                                  "cor__(Intercept).I(time^2)",
                                  "cor__time.I(time^2)",
                                  "sd__Observation", "nobs", "npeople" ))) %>%
  arrange(group, term) %>%
  mutate(term = as.character(term),
         term = gsub("\\(Intercept\\)", "Intercept", term),
         term = gsub("diabetes", "Diabetes", term),
         term = gsub("gender", "Gender", term),
         term = gsub("race", "Race", term),
         term = gsub("c.age", "Age", term),
         term = gsub("edu", "Education", term),
         term = gsub("purpose", "Purpose", term),
         #term = gsub("I(time^2)", "£\\\\textrm{time}\\\\^2£", term),
         term = gsub("time", "Time", term),
         term = gsub("I.+2.", "Time2", term),
         term = gsub("\\:", " x ", term),
         term = gsub("sd__(Intercept)", "$\\\\sigma_{\\\\textrm{Intercept}}$", term),
         term = gsub("sd__Time2", "$\\\\sigma_{\\\\textrm{Time2}}$", term),
         term = gsub("sd__Time", "$\\\\sigma_{\\\\textrm{Time}}$", term),
         term = gsub("cor__Time2", "$r_{\\\\textrm{Intercept},\\\\textrm{Time2}}$", term),
         term = gsub("cor__Intercept.Time", "$r_{\\\\textrm{Intercept},\\\\textrm{Time}}$", term),
         term = gsub("cor__Time.Time2", "$r_{\\\\textrm{Time},\\\\textrm{Time2}}$", term),
         term = gsub("sd__Observation", "$\\\\sigma_{\\\\textrm{Residual}}$", term),
         term = gsub("nobs", "$N_{\\\\textrm{obs}}$", term),
```

```
        term = gsub("npeople", "$N_{\\\\textrm{people}}$", term))

  fixef.smoker = which(smoker.data$effect == "fixed")
  rand.smoker = which(smoker.data$effect == "ran_pars")

smoker.data %>%
  dplyr::select(term, paste0(c("estimate_", "statistic_"), rep(smoker.names, each = 2))) %>%
  kable(., booktabs = T, caption = "Quadratic trajectories of smoking status", escape = F,
        col.names = c("Term", rep(c("$b(SE)$", "$t$"), length(smoker.names)))) %>%
  kable_styling(font_size = 7) %>%
  add_header_above(c(" ",
                     "ACL" = 2,
                     "HRS" = 2, "KGSS" = 2, "MIDJA" = 2,
                     "MIDUS" = 2, "SWAN" = 2, "WLS" = 2)) %>%
  landscape() %>%
  group_rows("Fixed Effects", min(fixef.smoker), max(fixef.smoker)) %>%
  group_rows("Random Effects", min(rand.smoker), max(rand.smoker)) %>%
  column_spec(c(2,4,6,8,10,12,14), width = "3em")
```

	ACL		HRS		KGSS		MIDJA		MIDUS		SWAN		WLS	
Term	$b(SE)$	t	$b(SE)$	t	$b(SE)$	t	$b(SE)$	t	$b(SE)$	t	$b(SE)$	t	$b(SE)$	t
**Random Effects**														
$\sigma_{\text{Intercept}}$	1.40		2.66		23.39		2.47		2.08		2.93		1.32	
σ_{Time}	5.54		13.70		11.43		4.55		5.02		5.87		0.85	
σ_{Time2}	0.43		4.89		11.12		0.95		0.59		0.60		0.05	
$r_{\text{Intercept,Time}}$	-1.00		-0.96		-0.97		-0.98		0.89		-0.94		-1.00	
$r_{\text{Intercept,Time2}}$	-1.00		0.57		-0.99		-0.97		0.88		-0.94		-1.00	
$r_{\text{Time,Time2}}$	1.00		-0.32		0.99		1.00		1.00		1.00		1.00	
**Fixed Effects**														
Intercept	3.14	1.04	0.66	1.19	-7.00	-0.11	2.97	0.77	0.34	0.09	-1.46	-0.43	3.95	0.42
	(3.03)		(0.55)		(61.30)		(3.87)		(3.87)		(3.40)		(9.39)	
Gender	-0.80	-0.94	0.26	1.51	1.38	0.44	-3.58	-1.88	0.00	0.00			-0.50	-0.89
	(0.86)		(0.17)		(3.11)		(1.90)		(0.90)				(0.56)	
Race	-0.34	-0.38	-0.03	-0.18					-1.24	-0.69	-0.35	-1.86		
	(0.89)		(0.19)						(1.79)		(0.19)			
Age	-1.54	-2.37	-0.87	-8.97	-0.45	-0.22	-0.44	-0.60	-0.72	-1.74	-0.33	-1.62	-0.28	-0.70
	(0.65)		(0.10)		(2.08)		(0.74)		(0.41)		(0.20)		(0.40)	
Education	-0.16	-0.87	-0.07	-2.64	-2.69	-1.47	-0.28	-0.88	0.10	0.60	-0.34	-3.99	-0.29	-1.87
	(0.18)		(0.03)		(1.83)		(0.31)		(0.17)		(0.08)		(0.15)	
Purpose	-0.37	-0.84	-0.22	-1.96	2.69	0.15	-0.67	-0.54	-1.30	-1.20	-0.31	-0.42	-1.66	-0.62
	(0.44)		(0.11)		(18.33)		(1.24)		(1.08)		(0.73)		(2.66)	
Time	-9.39	-0.33	3.90	0.84	1.64	0.05	-0.45	-0.32	0.68	0.27	2.78	0.46	0.08	0.15
	(28.70)		(4.66)		(30.78)		(1.42)		(2.50)		(6.04)		(0.51)	
Time2	-0.98	-0.34	0.75	0.48					0.06	0.21	0.29	0.48		
	(2.89)		(1.58)						(0.27)		(0.61)			
Time x Purpose	1.94	0.31	0.29	0.23	-0.63	-0.07	0.03	0.08	-0.46	-0.69	-0.31	-0.25	-0.08	-0.56
	(6.23)		(1.24)		(9.11)		(0.44)		(0.67)		(1.27)		(0.15)	
Purpose x Time2	0.20	0.32	-0.15	-0.34					-0.04	-0.54	-0.03	-0.22		
	(0.63)		(0.42)						(0.07)		(0.13)			
N_{obs}	204.00		14,654.00		44.00		97.00		538.00		2,106.00		236.00	
N_{people}	132.00		4,349.00		22.00		63.00		308.00		1,013.00		171.00	

Table 7: Quadratic trajectories of smoking status

7.3 Heart Condition status

```
# gather regression estimates into a data frame
# each row is one study
# each column is one regression model
heart.data = data.frame(study = heart.names)
heart.data$standard = lapply(X = heart.names,
                     FUN = function(x) get(paste0(x,"_out"))$regression$purpose$heart)

heart.data.n = data.frame(study = heart.names)
heart.data.n$npeople = unlist(lapply(X = heart.names,
                     FUN = function(x)
                       length(unique(get(paste0(x,"_out"))$regression$purpose$heart@frame$id))))
heart.data.n$nobs = unlist(lapply(X = heart.names,
                     FUN = function(x)
                       nrow(get(paste0(x,"_out"))$regression$purpose$heart@frame)))

heart.data.n = heart.data.n %>%
  gather(-study, key = "term", value = "estimate")


heart.data = heart.data %>%
  # gather so all regression models are in one column
  gather(key = "model", value = "value", -study) %>%
  # tidy
  mutate(tidy = map(value, broom::tidy)) %>%
  # unnest the tidy (the coefficients become the data frame)
  unnest(tidy) %>%
  full_join(heart.data.n) %>%
  # pull the estimates and standard errors into a single column
  mutate(estimate = ifelse(!is.na(std.error),
                         paste0(printnum(estimate), " (", printnum(std.error), ")"),
                         printnum(estimate)),
         estimate =ifelse(term %in% c("nobs", "npeople"), printnum(estimate, format = "d"), estimate),
         statistic = ifelse(!is.na(statistic), printnum(statistic), NA)) %>%
  dplyr::select(-std.error, -p.value) %>%
  gather(key = "key", value = "value",
         which(names(.) %in% c("estimate", "statistic"))) %>%
  # merge these two columns
  unite(key, key, study) %>%
  # spread back out. now each of our estimates and se's have their own columns
  spread(key = "key", value = "value") %>%
  mutate(term = factor(term,
                       levels = c("(Intercept)", "gender", "race",
                                  "c.age", "edu", "diabetes", "purpose",
                                  "purpose:diabetes",
                                  "time", "I(time^2)",
                                  "time:purpose",
                                  "purpose:I(time^2)",
                                  "sd__(Intercept)",
                                  "sd__time",
                                  "sd__I(time^2)",
                                  "cor__(Intercept).time",
                                  "cor__(Intercept).I(time^2)",
                                  "cor__time.I(time^2)",
                                  "sd__Observation", "nobs", "npeople" ))) %>%
  arrange(group, term) %>%
  mutate(term = as.character(term),
         term = gsub("\\(Intercept\\)", "Intercept", term),
         term = gsub("diabetes", "Diabetes", term),
         term = gsub("gender", "Gender", term),
         term = gsub("race", "Race", term),
         term = gsub("c.age", "Age", term),
         term = gsub("edu", "Education", term),
         term = gsub("purpose", "Purpose", term),
         #term = gsub("I(time^2)", "£\\\\textrm{time}\\\\^2£", term),
         term = gsub("time", "Time", term),
         term = gsub("I.+2.", "Time2", term),
         term = gsub("\\:", " x ", term),
         term = gsub("sd__(Intercept)", "$\\\\sigma_{\\\\textrm{Intercept}}$", term),
         term = gsub("sd__Time2", "$\\\\sigma_{\\\\textrm{Time2}}$", term),
         term = gsub("sd__Time", "$\\\\sigma_{\\\\textrm{Time}}$", term),
         term = gsub("cor__Time2", "$r_{\\\\textrm{Intercept},\\\\textrm{Time2}}$", term),
         term = gsub("cor__Intercept.Time", "$r_{\\\\textrm{Intercept},\\\\textrm{Time}}$", term),
         term = gsub("cor__Time.Time2", "$r_{\\\\textrm{Time},\\\\textrm{Time2}}$", term),
         term = gsub("sd__Observation", "$\\\\sigma_{\\\\textrm{Residual}}$", term),
         term = gsub("nobs", "$N_{\\\\textrm{obs}}$", term),
```

```
        term = gsub("npeople", "$N_{\\\\textrm{people}}$", term))

  fixef.heart = which(heart.data$effect == "fixed")
  rand.heart = which(heart.data$effect == "ran_pars")

heart.data %>%
  dplyr::select(term, paste0(c("estimate_", "statistic_"), rep(heart.names, each = 2))) %>%
  kable(., booktabs = T, caption = "Quadratic trajectories of heart condition status", escape = F,
        col.names = c("Term", rep(c("$b(SE)$", "$t$"), length(heart.names)))) %>%
  kable_styling(font_size = 7) %>%
  add_header_above(c(" ",
                     "ACL" = 2, "ELSA" = 2,
                     "HRS" = 2, "MIDJA" = 2,
                     "MIDUS" = 2, "SWAN" = 2, "WLS" = 2)) %>%
  landscape() %>%
  group_rows("Fixed Effects", min(fixef.heart), max(fixef.heart)) %>%
  group_rows("Random Effects", min(rand.heart), max(rand.heart)) %>%
  column_spec(c(2,4,6,8,10,12,14), width = "3em")
```

	ACL		ELSA		HRS		MIDJA		MIDUS		SWAN		WLS	
Term	$b(SE)$	t	$b(SE)$	t	$b(SE)$	t	$b(SE)$	t	$b(SE)$	t	$b(SE)$	t	$b(SE)$	t
**Random Effects**														
$\sigma_{\text{Intercept}}$	0.78		3.25		0.99		24.65		1.36		6.94		0.99	
σ_{Time}	1.94		0.45		18.70		8.70		1.41		4.66		1.43	
σ_{Time2}	0.40		2.76		11.70		3.24		0.15		1.16		0.16	
$r_{\text{Intercept,Time}}$	0.82		-1.00		-0.97		-0.56		0.88		0.99		-0.56	
$r_{\text{Intercept,Time2}}$	0.98		1.00		0.99		-0.83		0.88		0.99		0.21	
$r_{\text{Time,Time2}}$	0.92		-1.00		-0.99		0.93		1.00		1.00		0.69	
**Fixed Effects**														
Intercept	-6.45	-2.36	-36.41	-1.33	0.07	0.30	-27.62	-0.63	-1.53	-0.98	1.31	0.32	2.21	1.85
	(2.74)		(27.34)		(0.23)		(44.12)		(1.56)		(4.05)		(1.20)	
Gender	-0.18	-0.27	11.62	1.33	-0.33	-4.67	-0.14	-0.17	-0.46	-1.39			-0.56	-1.84
	(0.68)		(8.76)		(0.07)		(0.79)		(0.33)				(0.30)	
Race	1.12	1.66			-0.34	-4.09			-0.78	-1.29	0.90	1.24		
	(0.67)				(0.08)				(0.61)		(0.73)			
Age	0.43	1.38	4.20	1.17	0.38	9.90	1.02	2.26	0.52	3.45	2.02	2.00	0.53	1.45
	(0.31)		(3.60)		(0.04)		(0.45)		(0.15)		(1.01)		(0.37)	
Education	0.27	1.75	3.86	1.33	-0.01	-0.59	0.03	0.17	-0.07	-1.15	-0.12	-0.49	-0.06	-0.93
	(0.15)		(2.89)		(0.01)		(0.16)		(0.06)		(0.25)		(0.07)	
Purpose	0.01	0.02	3.56	0.90	-0.17	-3.59	5.63	0.48	0.43	1.17	-0.85	-1.00	-0.33	-1.65
	(0.30)		(3.95)		(0.05)		(11.75)		(0.37)		(0.85)		(0.20)	
Time	30.46	0.66	-6.87	-0.26	-4.15	-0.65	-6.17	-0.56	-0.35	-0.50	0.06	0.02	0.26	0.38
	(45.81)		(26.54)		(6.40)		(10.95)		(0.70)		(2.65)		(0.68)	
Time2	2.95	0.66	-0.75	-0.08	-4.30	-1.95			-0.02	-0.32	-0.25	-0.39	0.02	0.33
	(4.47)		(9.05)		(2.21)				(0.07)		(0.63)		(0.07)	
Time x Purpose	-6.57	-0.67	2.58	0.31	0.78	0.47	1.39	0.48	-0.08	-0.44	-0.19	-0.34	-0.06	-0.33
	(9.82)		(8.23)		(1.65)		(2.91)		(0.18)		(0.57)		(0.17)	
Purpose x Time2	-0.65	-0.68	0.49	0.18	-0.35	-0.61			-0.01	-0.72	0.01	0.08	-0.01	-0.68
	(0.96)		(2.71)		(0.57)				(0.02)		(0.13)		(0.02)	
N_{obs}	255.00		73.00		15,951.00		102.00		582.00		2,147.00		974.00	
N_{people}	132.00		17.00		4,530.00		64.00		308.00		1,014.00		439.00	

Table 8: Quadratic trajectories of heart condition status

8 Session Information

The analyses displayed here were run on a computer with the following settings:

```
## setting  value
## version  R version 4.2.3 (2023-03-15)
## os       macOS Big Sur ... 10.16
## system   x86_64, darwin17.0
## ui       X11
## language (EN)
## collate  en_US.UTF-8
## ctype    en_US.UTF-8
## tz       America/Los_Angeles
## date     2023-04-24
## pandoc   2.19.2 @ /Applications/RStudio.app/Contents/Resources/app/quarto/bin/tools/ (via rmarkdown)
```

The following packages were used:

	package	loadedversion	date
apaTables	apaTables	2.0.8	2021-01-04
assertthat	assertthat	0.2.1	2019-03-21
backports	backports	1.4.1	2021-12-13
bayestestR	bayestestR	0.13.1	2023-04-07
boot	boot	1.3-28.1	2022-11-22
broom	broom	1.0.4	2023-03-11
broom.helpers	broom.helpers	1.9.0	2022-09-23
broom.mixed	broom.mixed	0.2.9.4	2022-04-17
cachem	cachem	1.0.6	2021-08-19
callr	callr	3.7.3	2022-11-02
cellranger	cellranger	1.1.0	2016-07-27
cli	cli	3.6.1	2023-03-23
coda	coda	0.19-4	2020-09-30
codetools	codetools	0.2-19	2023-02-01
colorspace	colorspace	2.1-0	2023-01-23
crayon	crayon	1.5.2	2022-09-29
datawizard	datawizard	0.7.1	2023-04-03
DBI	DBI	1.1.3	2022-06-18
dbplyr	dbplyr	2.2.1	2022-06-27
devtools	devtools	2.4.5	2022-10-11
digest	digest	0.6.31	2022-12-11
dplyr	dplyr	1.1.1	2023-03-22
effectsize	effectsize	0.8.3	2023-01-28
effsize	effsize	0.8.1	2020-10-05
ellipsis	ellipsis	0.3.2	2021-04-29
emmeans	emmeans	1.8.5	2023-03-08
estimability	estimability	1.4.1	2022-08-05
evaluate	evaluate	0.20	2023-01-17
fansi	fansi	1.0.4	2023-01-22
fastmap	fastmap	1.1.0	2021-01-25
forcats	forcats	0.5.2	2022-08-19
fs	fs	1.6.1	2023-02-06
furrr	furrr	0.3.1	2022-08-15
future	future	1.27.0	2022-07-22
gargle	gargle	1.2.0	2021-07-02
generics	generics	0.1.3	2022-07-05

ggeffects	ggeffects	1.2.1	2023-04-02
ggplot2	ggplot2	3.4.2	2023-04-03
globals	globals	0.16.1	2022-08-28
glue	glue	1.6.2	2022-02-24
googledrive	googledrive	2.0.0	2021-07-08
googlesheets4	googlesheets4	1.0.1	2022-08-13
gt	gt	0.8.0	2022-11-16
gtable	gtable	0.3.3	2023-03-21
gtsummary	gtsummary	1.6.2	2022-09-30
haven	haven	2.5.1	2022-08-22
here	here	1.0.1	2020-12-13
highr	highr	0.10	2022-12-22
hms	hms	1.1.2	2022-08-19
htmltools	htmltools	0.5.3	2022-07-18
htmlwidgets	htmlwidgets	1.5.4	2021-09-08
httpuv	httpuv	1.6.6	2022-09-08
httr	httr	1.4.4	2022-08-17
insight	insight	0.19.1	2023-03-18
jsonlite	jsonlite	1.8.4	2022-12-06
kableExtra	kableExtra	1.3.4	2021-02-20
knitr	knitr	1.42	2023-01-25
later	later	1.3.0	2021-08-18
lattice	lattice	0.20-45	2021-09-22
lifecycle	lifecycle	1.0.3	2022-10-07
listenv	listenv	0.8.0	2019-12-05
lme4	lme4	1.1-32	2023-03-14
lubridate	lubridate	1.8.0	2021-10-07
magrittr	magrittr	2.0.3	2022-03-30
MASS	MASS	7.3-58.2	2023-01-23
mathjaxr	mathjaxr	1.6-0	2022-02-28
Matrix	Matrix	1.5-3	2022-11-11
memoise	memoise	2.0.1	2021-11-26
metadat	metadat	1.2-0	2022-04-06
metafor	metafor	4.0-0	2023-03-19
mime	mime	0.12	2021-09-28
miniUI	miniUI	0.1.1.1	2018-05-18
minqa	minqa	1.2.5	2022-10-19
mnormt	mnormt	2.1.0	2022-06-07
modelr	modelr	0.1.11	2023-03-22
multcomp	multcomp	1.4-20	2022-08-07
munsell	munsell	0.5.0	2018-06-12
mvtnorm	mvtnorm	1.1-3	2021-10-08
nlme	nlme	3.1-162	2023-01-31
nloptr	nloptr	2.0.3	2022-05-26
numDeriv	numDeriv	2016.8-1.1	2019-06-06
papaja	papaja	0.1.1	2022-07-05
parallelly	parallelly	1.32.1	2022-07-21
parameters	parameters	0.21.0	2023-04-19
performance	performance	0.10.3	2023-04-07
pillar	pillar	1.9.0	2023-03-22
pkgbuild	pkgbuild	1.4.0	2022-11-27
pkgconfig	pkgconfig	2.0.3	2019-09-22

pkgload	pkgload	1.3.2	2022-11-16
prettyunits	prettyunits	1.1.1	2020-01-24
processx	processx	3.8.1	2023-04-18
profvis	profvis	0.3.7	2020-11-02
promises	promises	1.2.0.1	2021-02-11
ps	ps	1.7.5	2023-04-18
psych	psych	2.2.5	2022-05-10
purrr	purrr	1.0.1	2023-01-10
R6	R6	2.5.1	2021-08-19
Rcpp	Rcpp	1.0.10	2023-01-22
readr	readr	2.1.2	2022-01-30
readxl	readxl	1.4.1	2022-08-17
remotes	remotes	2.4.2	2021-11-30
reprex	reprex	2.0.2	2022-08-17
rlang	rlang	1.1.0	2023-03-14
rmarkdown	rmarkdown	2.16	2022-08-24
rprojroot	rprojroot	2.0.3	2022-04-02
rstudioapi	rstudioapi	0.14	2022-08-22
rvest	rvest	1.0.3	2022-08-19
sandwich	sandwich	3.0-2	2022-06-15
SAScii	SAScii	1.0.1	2022-04-27
scales	scales	1.2.1	2022-08-20
sessioninfo	sessioninfo	1.2.2	2021-12-06
shiny	shiny	1.7.2	2022-07-19
sjlabelled	sjlabelled	1.2.0	2022-04-10
sjmisc	sjmisc	2.8.9	2021-12-03
sjPlot	sjPlot	2.8.14	2023-04-02
sjstats	sjstats	0.18.2	2022-11-19
stringi	stringi	1.7.12	2023-01-11
stringr	stringr	1.5.0	2022-12-02
survival	survival	3.5-3	2023-02-12
svglite	svglite	2.1.0	2022-02-03
systemfonts	systemfonts	1.0.4	2022-02-11
TH.data	TH.data	1.1-1	2022-04-26
tibble	tibble	3.2.1	2023-03-20
tidyr	tidyr	1.3.0	2023-01-24
tidyselect	tidyselect	1.2.0	2022-10-10
tidyverse	tidyverse	1.3.2	2022-07-18
tinylabels	tinylabels	0.2.3	2022-02-06
tzdb	tzdb	0.3.0	2022-03-28
urlchecker	urlchecker	1.0.1	2021-11-30
usethis	usethis	2.1.6	2022-05-25
utf8	utf8	1.2.3	2023-01-31
vctrs	vctrs	0.6.1	2023-03-22
viridisLite	viridisLite	0.4.1	2022-08-22
webshot	webshot	0.5.3	2022-04-14
withr	withr	2.5.0	2022-03-03
xfun	xfun	0.38	2023-03-24
xml2	xml2	1.3.3	2021-11-30
xtable	xtable	1.8-4	2019-04-21
zoo	zoo	1.8-10	2022-04-15
